# Deferiprone: Pan-selective Histone Lysine Demethylase Inhibition Activity and Structure Activity Relationship Study

**DOI:** 10.1038/s41598-019-39214-1

**Published:** 2019-03-18

**Authors:** Verjine Khodaverdian, Subhasish Tapadar, Ian A. MacDonald, Yuan Xu, Po-Yi Ho, Allison Bridges, Pragya Rajpurohit, Bhakti A. Sanghani, Yuhong Fan, Muthusamy Thangaraju, Nathaniel A. Hathaway, Adegboyega K. Oyelere

**Affiliations:** 1 School of Chemistry and Biochemistry, Georgia Institute of Technology, Atlanta, GA 30332-0400 USA; 2 The University of North Carolina Eshelman School of Pharmacy, Chapel Hill, NC 27599 USA; 3 School of Biological Sciences, Georgia Institute of Technology, Atlanta, GA 30332-0400 USA; 4 Medical College of Georgia, Augusta University, Augusta, GA 30912 USA; 5 Parker H. Petit Institute for Bioengineering and Bioscience, Georgia Institute of Technology, Atlanta, GA 30332-0400 USA

## Abstract

Deferiprone (DFP) is a hydroxypyridinone-derived iron chelator currently in clinical use for iron chelation therapy. DFP has also been known to elicit antiproliferative activities, yet the mechanism of this effect has remained elusive. We herein report that DFP chelates the Fe^2+^ ion at the active sites of selected iron-dependent histone lysine demethylases (KDMs), resulting in pan inhibition of a subfamily of KDMs. Specifically, DFP inhibits the demethylase activities of six KDMs - 2A, 2B, 5C, 6A, 7A and 7B - with low micromolar IC_50_s while considerably less active or inactive against eleven KDMs - 1A, 3A, 3B, 4A-E, 5A, 5B and 6B. The KDM that is most sensitive to DFP, KDM6A, has an IC_50_ that is between 7- and 70-fold lower than the iron binding equivalence concentrations at which DFP inhibits ribonucleotide reductase (RNR) activities and/or reduces the labile intracellular zinc ion pool. In breast cancer cell lines, DFP potently inhibits the demethylation of H3K4me3 and H3K27me3, two chromatin posttranslational marks that are subject to removal by several KDM subfamilies which are inhibited by DFP in cell-free assay. These data strongly suggest that DFP derives its anti-proliferative activity largely from the inhibition of a sub-set of KDMs. The docked poses adopted by DFP at the KDM active sites enabled identification of new DFP-based KDM inhibitors which are more cytotoxic to cancer cell lines. We also found that a cohort of these agents inhibited HP1-mediated gene silencing and one lead compound potently inhibited breast tumor growth in murine xenograft models. Overall, this study identified a new chemical scaffold capable of inhibiting KDM enzymes, globally changing histone modification profiles, and with specific anti-tumor activities.

## Introduction

**Figure 1:**
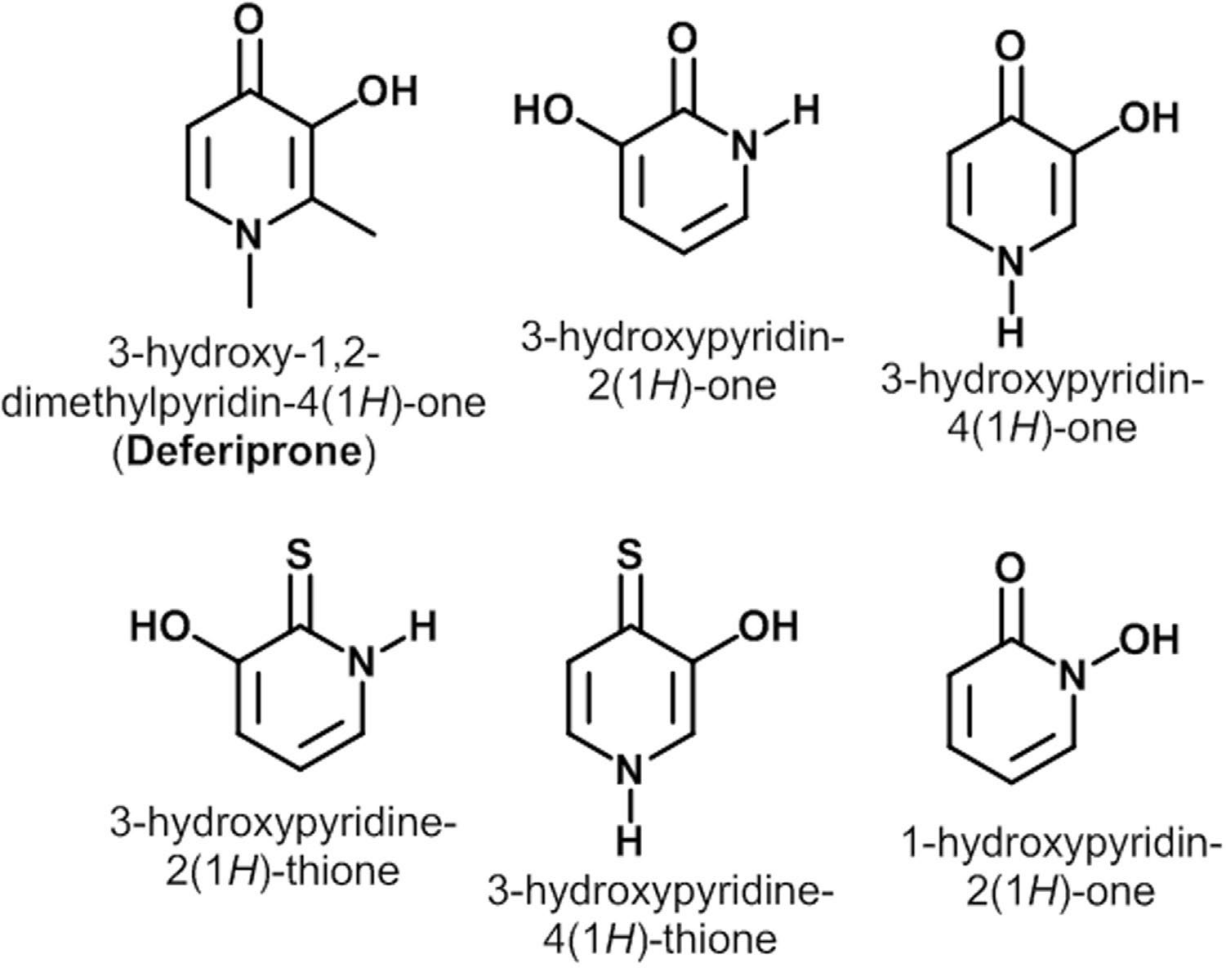
Representative examples of hydroxypyridinone bidentate metal ion chelators.

Deferiprone (DFP) is a bidentate iron chelator approved for the treatment of iron-overloaded patients with thalassemia^1,2^. DFP is a type of hydroxypyridinone (Fig. 1) which preferentially binds free iron in ferric state (Fe^3+^) in a 3:1 ratio. Unlike desferrioxamine (DFO), the first line agent for the treatment of transfusional iron overload, DFP is orally active. The concomitant effect of iron chelation by DFP is the reversal of oxidative stress related tissue damage in iron overload^1^. DFP and other iron chelators were further shown to elicit antiproliferative activity against various tumor cell lines and lymphocytes^3–7^.

On the account that high levels of iron are essential for tumor cell growth, the antiproliferative effect of DFP has been largely attributed to its iron chelation activity which results in the depletion of free intracellular iron and removal of iron from the active sites of key iron-dependent enzymes. Specifically, it has been shown that DFP could remove iron from mammalian ribonucleotide reductase (RNR) in leukemia K562 cells^8,9^, leading to the inactivation of RNR, inhibition of DNA synthesis, cell cycle arrest and cell growth inhibition^3,4,8^. However, DFP is not an iron-specific chelator. Like other hydroxypyridinone, DFP also binds biological divalent metal ions Cu^2+^ and Zn^2+^ with high affinity and other metal ions such as Ca^2+^, Mg^2+^, Na^+^, and K^+^ with low affinity^10–13^. In fact, depletion of intracellular Zn^2+^ pool has been suggested to be a major contributing factor to the DFP-induced apoptosis in thymocyte and other proliferating T lymphocytes^6,14^. The small flat aromatic structure of DFP could fit into active sites of several intracellular metalloenzymes and the inhibition of these metalloenzymes could in principle contribute to the anti-proliferative activity of DFP. Therefore, DFP could derive its cell growth inhibition from convergence of several mechanisms the details of which are poorly understood.

Using a fragment-based molecular docking approach, we have interrogated in previous studies the interaction of a small library of bidentate zinc/iron chelators derived from hydroxypyridinones with a subset of histone deacetylase (HDAC) isoforms. We identified 3-hydroxypyridin-2-thione as a zinc binding group that chelates Zn^2+^ ion at the active site of HDAC6 and HDAC8, resulting in robust inhibition of the activities of these HDAC isoforms^15,16^. HDACs are a class of zinc-dependent epigenetic modifiers^17^. For those HDAC isoforms that have been subject to structural characterization, the architecture of the enzymes’ active sites is nearly identical, consisting of Zn^2+^ ion bound to the base of the active site pocket that is in turn exposed to the enzyme surface through a short channel lined with hydrophobic residues. Another class of epigenetic modifiers whose active sites architecture resemble HDACs’ is 2-oxoglutarate- and Fe^2+^-dependent histone lysine demethylases (KDMs) that remove specific histone methylation posttranslational marks^18–22^. In this study, we adopted a similar molecular docking analysis from our previous studies to evaluate the possibility that a library of hydroxypyridinone-derived bidentate zinc/iron chelators, including DFP, interacts with representative KDMs. We observed that DFP chelates the active site Fe^2+^ ion. A subsequent cell-free assay revealed that DFP possesses pan-selective inhibition activity against a subfamily of KDMs. Specifically, DFP inhibits the demethylase activities of six KDMs - 2A, 2B, 5C, 6A, 7A and 7B - at low micromolar IC_50_s. DFP is considerably less active or inactive against eleven KDMs - 1A, 3A, 3B, 4A-E, 5A, 5B and 6B. The KDM that is most sensitive to DFP, KDM6A, has an IC_50_ that is between 7- and 70-fold lower than the iron binding equivalence concentrations at which DFP inhibits RNR activities and/or reduces labile intracellular zinc ion pool^8,9,14^. Moreover, in MCF-7 and MDA-MB-231 cells, DFP reduces levels of H3K4me3 and H3K27me3, indicating potential action as a KDM inhibitor. These two chromatin posttranslational marks are subject to demethylation by several KDM subfamilies which are inhibited by DFP in our cell-free assay.

Encouraged by the foregoing data, we conducted a comprehensive structure activity relationship study on DFP. This study furnished several novel DFP-based KDM inhibitors which alter the velocity of HP1-mediated heterochromatin gene repression. Additionally, these compounds displayed tumor-selective cytotoxicity against the breast cancer (BCa) cell lines tested, with potency enhancement as high as 65-fold relative to DFP. These compounds are preferentially more cytotoxic to the triple negative breast cancer (TNBC) cell line MDA-MB-231 and a representative compound significantly reduce tumor growth in xenograft mouse models of ER(+) (T47D) and ER(−) (MDA-MB-231) BCas. Collectively, we disclose herein that clinically approved DFP derives its anti-proliferative activity largely from the inhibition of a sub-set of KDMs. Excitingly, our findings demonstrate that DFP can serve as a novel template for the discovery of tumor-selective KDM inhibitors.

## Results

### DFP binds to Fe^2+^ ion at the active site of KDM6A

**Figure 2:**
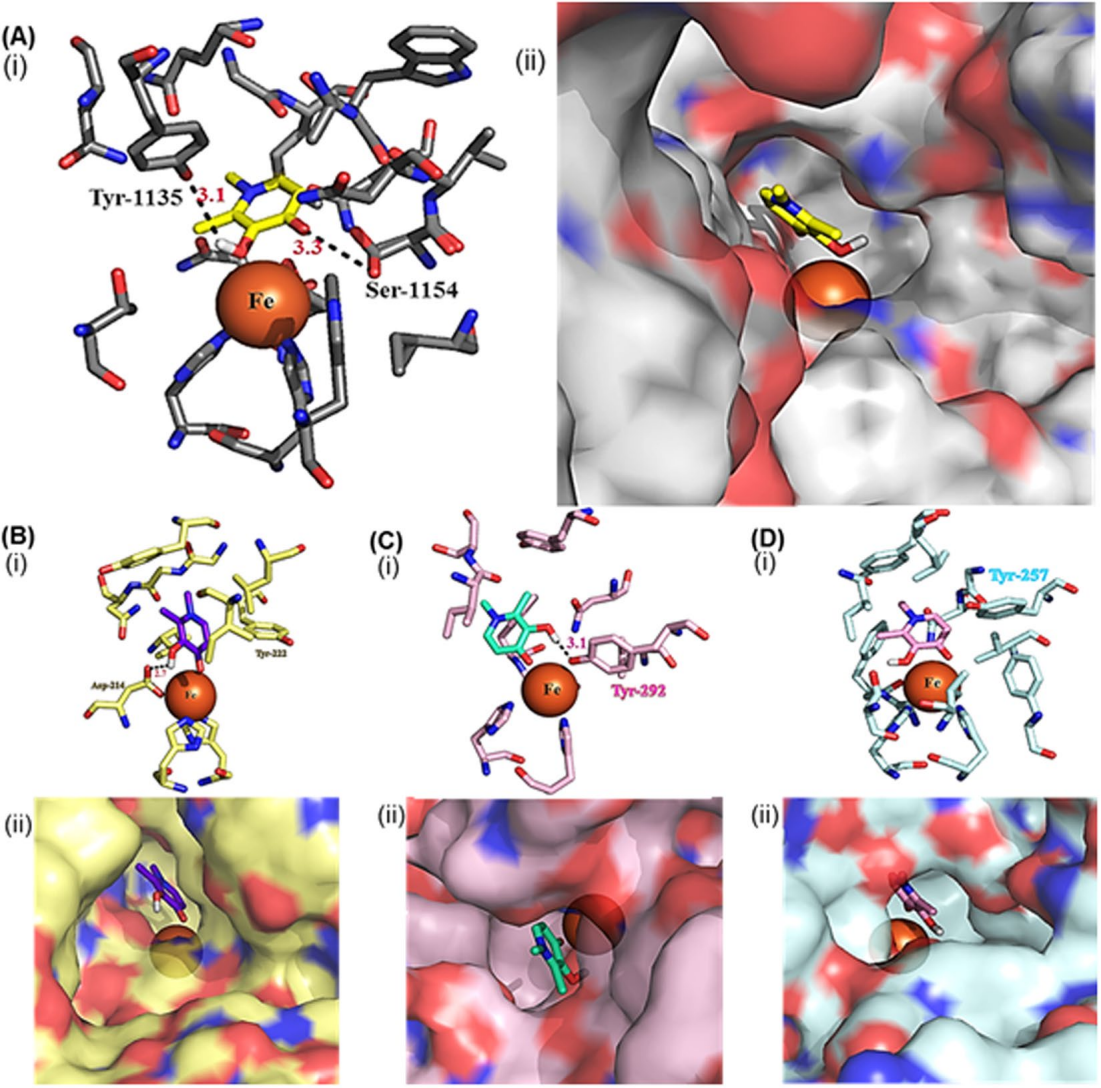
Docked poses of DFP on representative KDMs. (**A**) DFP binds to the active site of KDM6A (PDB: 3AVR, gray)^18^, (i) through chelation to the active site Fe^2+^ and H-bonding interaction with the phenolic group of Tyr 1135 and OH group of Ser 1154, (ii) adopting a docked pose having its N-1 moiety oriented toward the exit channel of the active site. (**B**) DFP interacts with KDM2A (PDB: 4QXB, yellow)^19^ (i) through Fe^2+^ chelation and H-bonding interactions to Tyr 222 and Asp 214, and (ii) with the N-1 moiety oriented away from the exit channel, (**C**) DFP interacts with KDM7A (PDB: 3KV5, pink)^20^ (i) through Fe^2+^chelation and H-bonding to Try 292, and (ii) the N-1 moiety adopts a similar orientation as in KDM6A, (**D**) DFP interacts with KDM7B (PDB: 3KV4, blue)^20^ docked position with (i) through Fe^2+^chelation and (ii) the N-1 group is buried within the active site.

We used molecular docking to interrogate the interaction of DFP with KDM6A, a representative KDM which regulates gene expression programs associated with BCa proliferation and invasion^23,24^. This docking analysis built on our previous studies where we used the molecular docking program AutoDock 4.2^25^ to successfully identify from this library 3-hydroxypyridin-2-thione as a non-hydroxamate zinc binding group that is compatible with HDAC inhibition^15,16^. Analyses of the molecular docking outputs revealed that DFP adopts docked poses in which it strongly chelates Fe^2+^ at the active site of KDM6A. In addition to iron chelation, the interaction is further stabilized by H-bonding between the oxygen moieties of DFP and two key residues within the enzymes active sites: phenolic group of Tyr 1135 and OH group of Ser 1154. The N-1 position of the DFP is oriented toward the exit channel of the active site (Fig. 2A), an orientation that should permit modifications which may enhance KDM binding affinity. Subsequent molecular dockings with KDMs 2A, 7A, and 7B revealed that DFP adopted docked poses and maintained similar interaction as the ones in KDM6A (Fig. 2B–D) while devoid of interaction with KDM5A (Fig. S1) and Flavin-dependent KDM1A.

### DFP inhibits a sub-set of KDMs in *in vitro* assay

**Figure 3:**
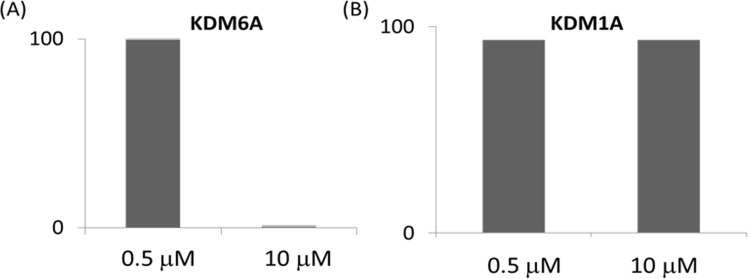
DFP inhibits the activity of Fe^2+^-dependent KDM6A (**A**) while inactive against Flavin-dependent KDM1A (**B**). Y-axis shows the % residual enzyme activity.

To test the validity of our *in silico* predictions, we first investigated the effects of DFP on activities of KDM1A and KDM6A at two concentrations − 0.5 and 10 μM. We observed that DFP showed a concentration dependent inhibition of KDM6A, resulting in >95% reduction in the enzyme activity at 10 μM. In contrast, DFP has no effect on the activity of KDM1A at both concentrations (Fig. 3). Subsequently, we determined the effects of DFP on the enzymatic activities of seventeen recombinant human KDMs using a cell-free enzymatic assay. We observed that DFP inhibits the demethylase activities of six KDMs - 2A, 2B, 5C, 6A, 7A and 7B - at low micromolar IC_50_s while considerably less active or inactive against eleven KDMs - 1A, 3A, 3B, 4A-E, 5A, 5B and 6B (Table 1, Fig. S2).

**Table 1:** *In vitro* inhibitory activities of DFP against seventeen recombinant human KDMs.

KDMs	IC_50_ (μM)
KDM1A (LSD1)	NI^a^
KDM2A (FBXL10)	6.8
KDM2B (FBXL11)	8.1
KDM3A (JMJD1A)	NI^b^
KDM3B (JMJD1B)	NI^b^
KDM4A (JMJD2A)	NI^a^
KDM4B (JMJD2B)	NI^b^
KDM4C (JMJD2C)	NI^b^
KDM4D (JMJD2D)	NI^b^
KDM4E (JMJD2E)	NI^b^
KDM5A (Jarid1A)	NI^b^
KDM5B (Jarid1B)	NI^b^
KDM5C (Jarid1C)	5.6
KDM6A (UTX)	4.2
KDM6B (JMJD3)	NI^b^
KDM7A (JHDM1D)	16.6
KDM7B (PHF8)	15.6

^a^No inhibition at maximum tested concentration of 25 μM.

^b^Less than 50% inhibition at maximum tested concentration of 25 μM.

### DFP potently inhibits H3K27me3 demethylation in MCF-7 and MDA-MB-231 cells

**Figure 4:**
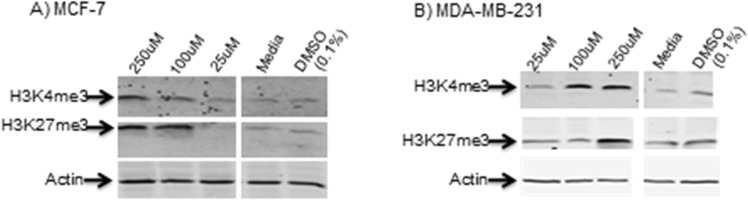
DFP elicits dose-dependent increase in the H3K4me3 and H3K27me3 levels in (**a**) MCF-7 and (**b**) MDA-MB-231 cells. Shown at the top of each gel is the concentration of DFP in μM. Western blotting with an anti-β-Actin antibody indicates equal loading of proteins in each lane (lower gel). The media and DMSO (0.1%) lanes showed basal levels of H3K4me3 and H3K27me3 and the effects of assay buffers on H3K4me3 and H3K27me3 levels, respectively. Shown here are grouping of gels cropped from different parts of the same gel and/or from different gels. Representative full-length gels are included in a Supplementary Information (Fig. S3).

To confirm if the KDM inhibition activities displayed by DFP in the cell-free assay translated to intracellular effects, we performed Western blot analyses on cell lysates from MCF-7 and MDA-MB-231 cells treated with various concentration of DFP. We probed for the levels of H3K4me3 and H3K27me3, two chromatin posttranslational marks that are subject to demethylation by several KDM subfamilies, including KDM 2B^26^, 5C^27^ and 6A^28^ which are inhibited by DFP in our cell-free assay. We observed that DFP induced a dose-dependent upregulation of H3K4me3 and H3K27me3 levels in these cells (Fig. 4). This observation strongly suggests that DFP potently inhibits H3K4me3 and H3K27me3 demethylation intracellularly.

### Structure Activity Relationship (SAR) Studies

**Figure 5:**
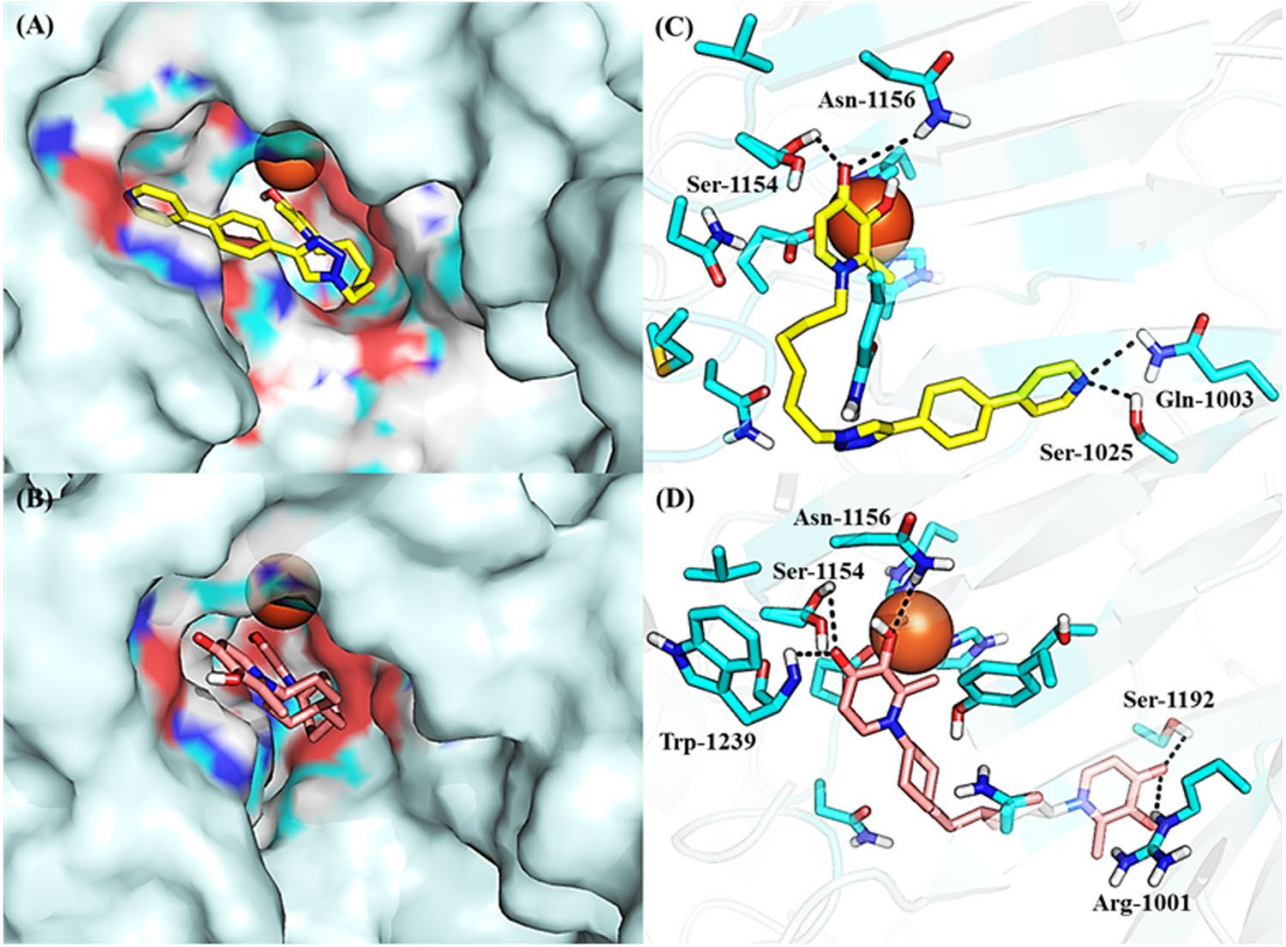
Docked poses of representative DFP-derivatives at active site of KDM6A. (**A**) **2** **u** (yellow), and (**B**) **3g** (peach) adopt orientation at the active site of KDM6A (PDB: 3AVR, light blue)^18^, that are similar to that of DFP with their N-1 moiety oriented toward the exit channel of the active site. In addition to the interaction with the active site Fe^2+^ (orange), (**C**) **2u** could form additional H-bonding interactions with Asn 1156, Ser 1154, Gln 1003 and Ser 1025 (**D**) while **3g** could form additional H-bonding interactions with Asn 1156, Ser 1154, and Trp 1239 near active site Fe^2+^, and guanidine of Arg 1001 and Ser 1192 near the exit tunnel (**D**).

**Figure 6:**
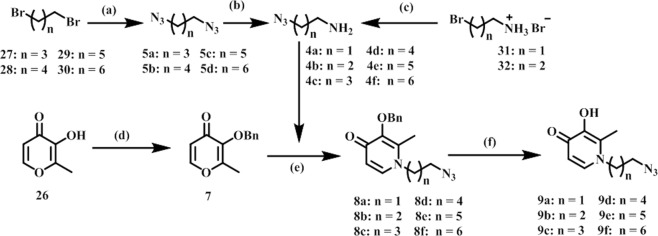
Synthesis of Azidoalkyl-3-hydroxypyridin-4-one. Reagents and conditions: (**a**) NaN_3_, DMF, 80 °C, 20 h; (**b**) PPh_3_, 5% HCl, EtOAc:Ether, 40 h; (**c**) (1) NaN_3_, H_2_O, 80 °C, 12 h; (2) aq KOH; (**d**) benzyl chloride, K_2_CO_3_, DMF, 110 °C, 3 h; (**e**) NaOH, EtOH:H_2_O, 110 °C under pressure, 72 h; (**f**) conc. HCl, 4–6 h.

**Figure 7:**
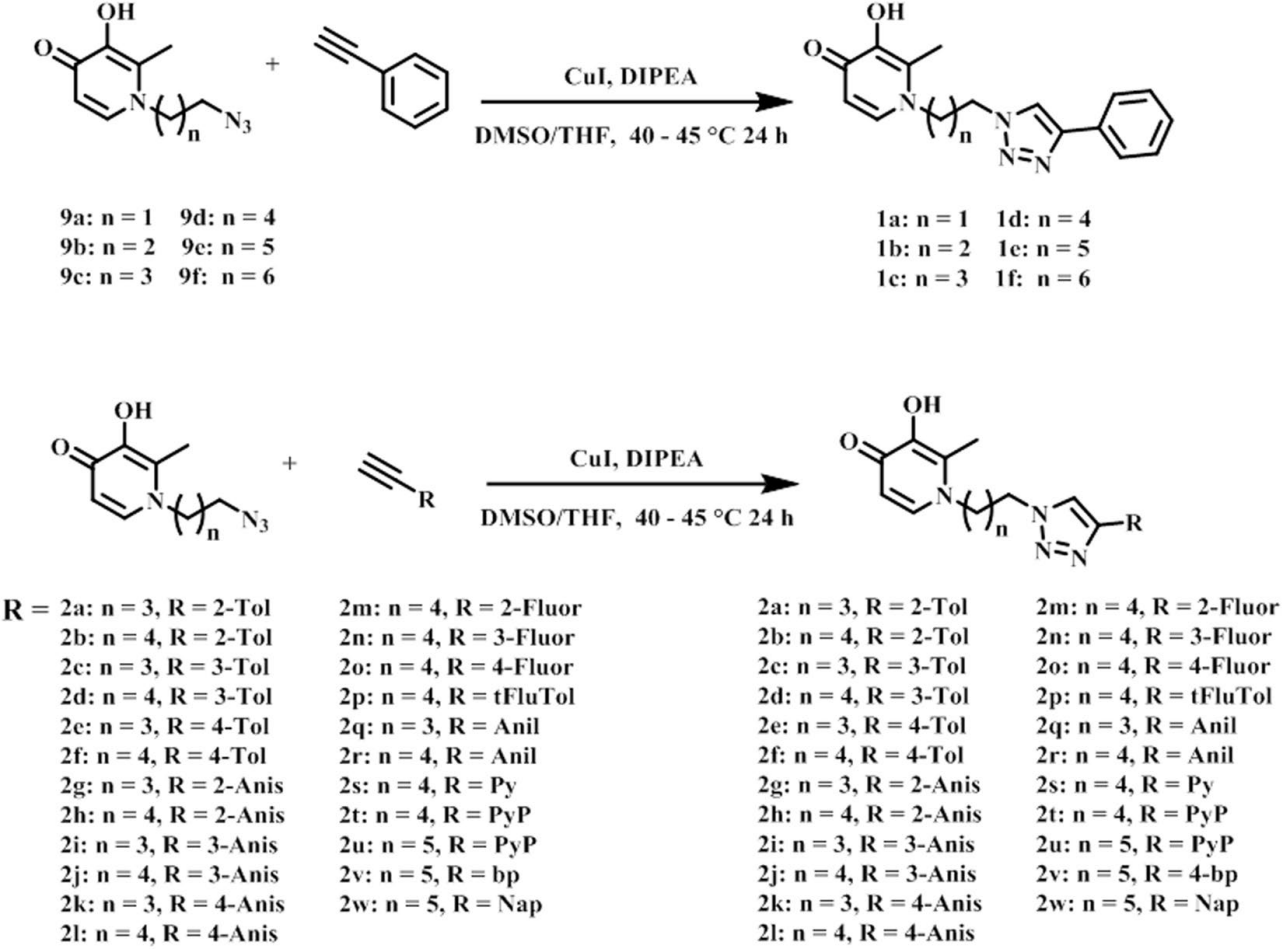
Synthesis of triazole containing KDM inhibitors (**1a–f** and **2a–w**). *Abbreviation*: 2-Tol, 2-tolyl; 3-Tol, 3-tolyl; 4-Tol, 4-tolyl; 2-Anis, 2-anisolyl; 3-Anis, 3-anisolyl; 4-Anis, 4-anisolyl; 2-Fluor, 2-fluoryl; 3-Fluor, 3-fluoryl; 4-Fluor, 4-fluoryl; tFluTol; α,α,α-trifluorotolyl; Anil, 4-anilyl; 4-Py, 4-pyridyl; PyP, 4-pyridylphenyl; 4-bp, 4-biphenyl; Nap, 6-methoxynapthyl.

**Figure 8:**
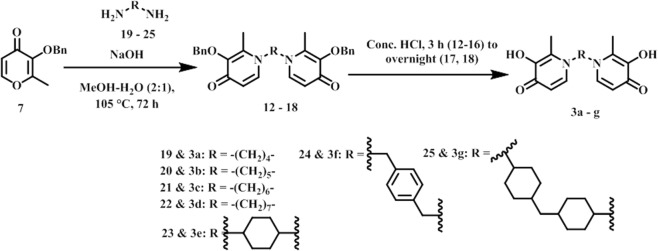
Synthesis of BisDFP KDM inhibitors (**3a–g**).

Our docking analysis revealed that the N-1 moiety of DFP is presented toward an exit channel, away from the active sites of several KDMs (Fig. 2). To explore if interaction within this channel will result in enhancement of potency, we designed three distinct structural classes of DFP analogs (Classes I (unsubstituted aryl **1a–f**), II (substituted aryl **2a–w**) and III (bisDFP **3a–g**), building from the N-1 position. Molecular docking analysis revealed that representatives of these compounds optimally occupied the channel while maintaining Fe^2+^ chelation (Fig. 5). The syntheses of compounds **1–3** were accomplished as shown in Figs 6–8. Compounds **9a–f**, the azido precursors of **1** and **2** were synthesized from benzyl-protected maltol **7** and appropriate azido amines **4a–f** through the intermediacy of **8a–f** (Fig. 6). Subsequently, CuI mediated Huigsen cyclization between **9a–f** and appropriate aryl alkynes furnished compounds **1a–f** and **2a–w** low to good yields (Figs 6 and 7). BisDFP compounds **3a–g** were synthesized in two steps starting from benzyl-protected maltol **7** and diamino compounds **19**–**25** (Fig. 8). A suspension of **7**, an appropriate diamino compound and sodium hydroxide in methanol-H_2_O (2:1) was heated at 105 °C for 72 h in a sealed tube. The protected bisDFP compounds **12**–18 were treated with concentrated HCl solution for 3 h to overnight to get the target compounds **3a–g** in low to moderate yields.

To preliminarily profile the bioactivity of these compounds, we adopted a streamlined approach which involved screening against KDM6A at two concentrations and evaluating antiproliferative activity against three representative cell lines – MCF-7 (ER(+) BCa), MDA-MB-231 (TNBC BCa) and Vero (a non-transformed cell line as control). Similar to DFP, these compounds caused concentration dependent inhibition of KDM6A, resulting in 96–100% reduction in the enzyme activity at 10 μM (Tables 2–3).

**Table 2:** KDM6A activity inhibition (%) of selected compounds **1** and **2** at 1 μM and 10 μM.


KDM6A (UTX) Inhibition (%)				
Compd	R	n	1 (μM)	(10 μM)
**1a**		1	12	96
**1b**		2	6	100
**1c**		3	8	100
**1d**		4	12	100
**1e**		5	12	98
**1f**		6	10	99
**2a**		3	6	100
**2c**		3	4	100
**2e**		3	13	100
**2h**		4	3	99
**2i**		3	7	100
**2j**		4	8	100
**2l**		4	4	100
**2m**		4	16	100
**2n**		4	5	100
**2o**		4	10	100
**2p**		4	15	99
**2s**		4	15	99
**2t**		4	8	99
**2u**		5	15	99
**2v**		5	12	49
**2w**		5	16	57

**Table 3:** KDM6A activity inhibition (%) of **3a–g** at 1 μM and 10 μM.


KDM6A (UTX) Inhibition (%)			
Compd	R	1 (μM)	(10 μM)
**3a**		4	100
**3b**		1	99
**3c**		2	100
**3d**		3	100
**3e**		5	100
**3f**		5	100
**3g**		9	100

**Figure 9:**
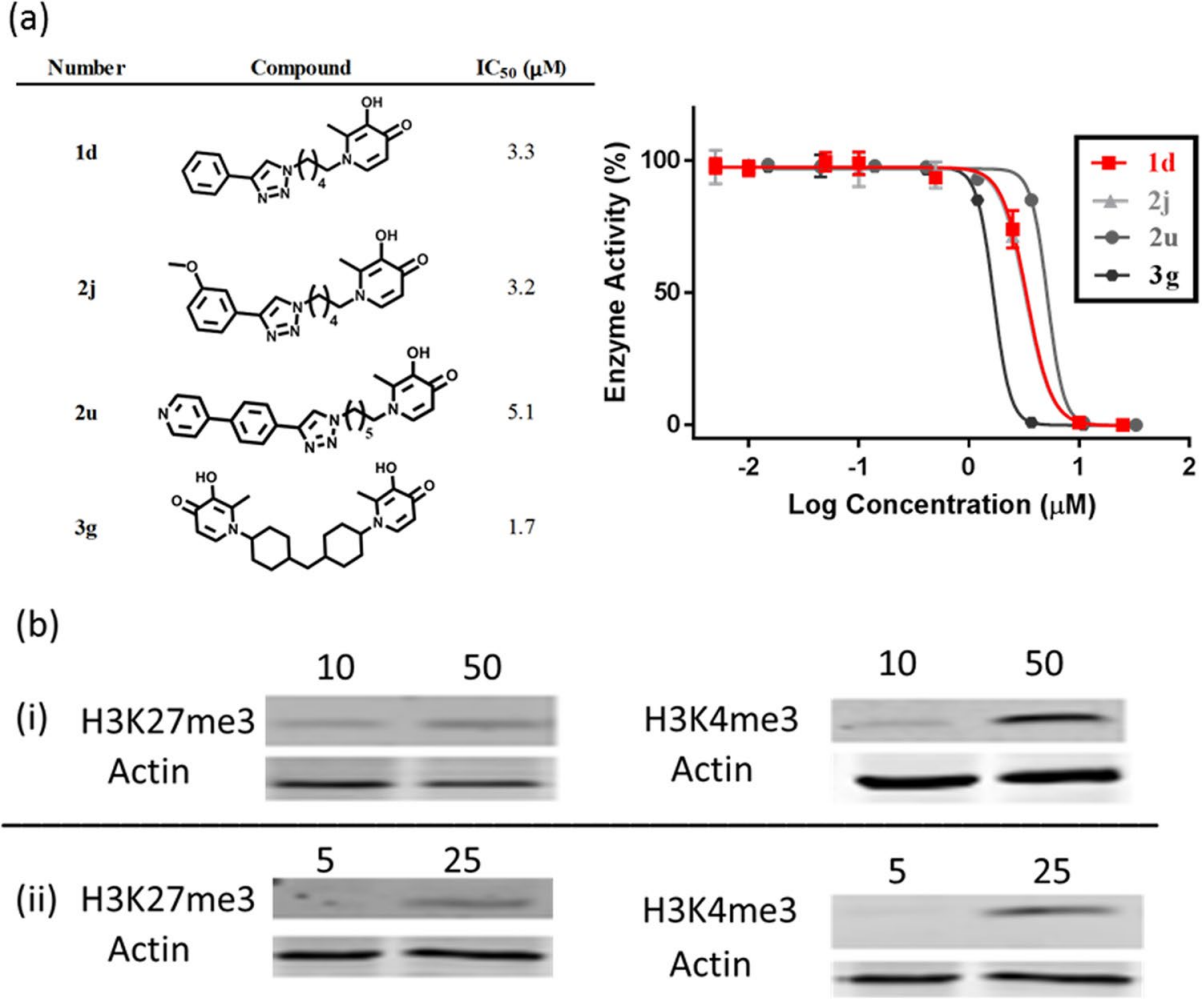
**(A)** Dose response curve of KDM6A inhibition by **1d** (red), **2j** (light gray), **2u** (gray), and **3g** (black). (**B**) Compound **1d** (i) and **2u** (ii) caused dose-dependent increase in the H3K4me3 and H3K27me3 levels in MDA-MD-231 cells (upper gels). Shown at the top of each gel is the concentration of each compound in μM. Western blotting with an anti-β-Actin antibody indicates equal loading of proteins in each lane (lower gels). Shown here are grouping of gels cropped from different parts of the same gel and/or from different gels.

Subsequently, we observed that a subset of compounds from each class displayed tumor-selective cytotoxicity against the BCa cell lines tested, with potency enhancement as high as 93-fold relative to DFP (Tables 4–6, Figs S4–S7). Intriguingly, these compounds are preferentially more cytotoxic to the TNBC cell line MDA-MB-231. With the exception of **2a**, **2c**, **2g**, **2i**, **2j**, **2t**, **3f** and **3g**, these compounds reduced the viability of MCF-7 cell line by less than 50% at the maximum tested concentrations under the same conditions used for the MDA-MB-231 and Vero cell lines (Supp Info Figs S5–S7). BCa cell lines have been shown to elicit distinct sensitivity to other KDM inhibitors, possibly due to disparities in the expression levels and roles of different KDM subfamilies in the viability of these cells^29–31^. Encouraged by these positive attributes, we determined the IC_50_ of representative potent members of each class – **1d**, **2j**, **2u** and **3g** against KDM6A. We found that they inhibited KDM6A with low micromolar IC_50_ (Fig. 9A). Relative to DFP however, the KDM6A inhibition potencies of these lead compounds are enhanced by 1.3 to 2.5-fold and this does not completely explain the observed ~31–93-fold enhancement of the cytotoxicity of the best compounds in this series to MDA-MB-231 (Tables 5–6). This may be due to several factors including reduced tendency of **1d**, **2j**, **2u** and **3g**, relative to DFP, to form tridentate complex with intracellularly free iron in ferric state (Fe^3+^)^32^, the difference in their preference for KDM subfamily, or their improved cell penetration. We then performed Western blot analyses on cell lysates from MDA-MB-231 cells treated with various concentrations of **1d** and **2u** to probe if the KDM inhibition activities in the cell-free assay translate to intracellular effect. We observed that **1d** and **2u** induced a dose-dependent increase in the H3K4me3 and H3K27me3 levels at concentrations that are 10-times lower than those of DFP (Fig. 9B). This observation may indicate that **1d** and **2u** have better cell penetration properties than DFP, an attribute which may partly explain the enhanced antiproliferative activities of these DFP-based KDM inhibitors.

**Table 4:** Antiproliferative activity of compounds **1a–f** and DFP.


Antiproliferative Activity IC_50_ (μM)				
Compd	n	MDA-MB-231	MCF-7	VERO
**1a**	1	>50	ND^a^	>100
**1b**	2	25.1	ND	>50
**1c**	3	10.5	ND	>50
**1d**	4	11.4	ND	>50
**1e**	5	12.5	ND	>35
**1f**	6	16.4	ND	17.6
**DFP**	—	111.9	134.1	85.9

^a^ND, not determinable; cell viability <50% at the maximum tested dose.

**Table 5:** Antiproliferative activity of compounds **2a–w**.


Antiproliferative Activity IC_50_ (μM)					
Compd	R	n	MDA-MB-231	MCF-7	VERO
**2a**		3	16.4	42.5^a^	11.6
**2b**		4	12.7	ND^b^	5.8
**2c**		3	10.9	62.7^a^	5.2
**2d**		4	11.8	ND	3.6
**2e**		3	7.4	ND	4.1
**2f**		4	15.7	ND	3.1
**2g**		3	9.6	66.1^a^	>50
**2h**		4	19.1	ND	>50
**2i**		3	7.5	54.8^a^	>50
**2j**		4	10.6	34.1^a^	>50
**2k**		3	8.1	ND	>50
**2l**		4	9.6	ND	>50
**2m**		4	18.3	ND	12.7
**2n**		4	6.8	ND	22.9
**2o**		4	3.6	ND	38.8
**2p**		4	18.8	ND	13.3
**2q**		3	9.5	ND	>50
**2r**		4	12.5	ND	>50
**2s**		4	NI^c^	ND	NI
**2t**		4	3.6	37.6^a^	29.2
**2u**		5	1.7	ND	66.9
**2v**		5	2.5	ND	4.9
**2w**		5	1.2	ND	3.9

^a^Values calculated by linear regression.

^b^ND, not determinable; cell viability <50% at the maximum tested dose.

^c^NI, no inhibition.

**Table 6:** Antiproliferative activity of compounds **3a–g**.


Antiproliferative Activity IC_50_ (μM)				
Compd	R	MDA-MB-231	MCF-7	VERO
**3a**		NI^a^	NI	NI
**3b**		NI	NI	NI
**3c**		NI	NI	NI
**3d**		NI	NI	NI
**3e**		36.9	ND^b^	NI
**3f**		25.0	378.4^c^	NI
**3g**		3.5	31.1^c^	>50

^a^NI, no inhibition.

^b^ND, not determinable; cell viability <50% at the maximum tested dose.

^c^Values calculated by linear regression.

### DFP-based KDM inhibitors slow HP1-stimulated heterochromatin gene repression

**Figure 10:**
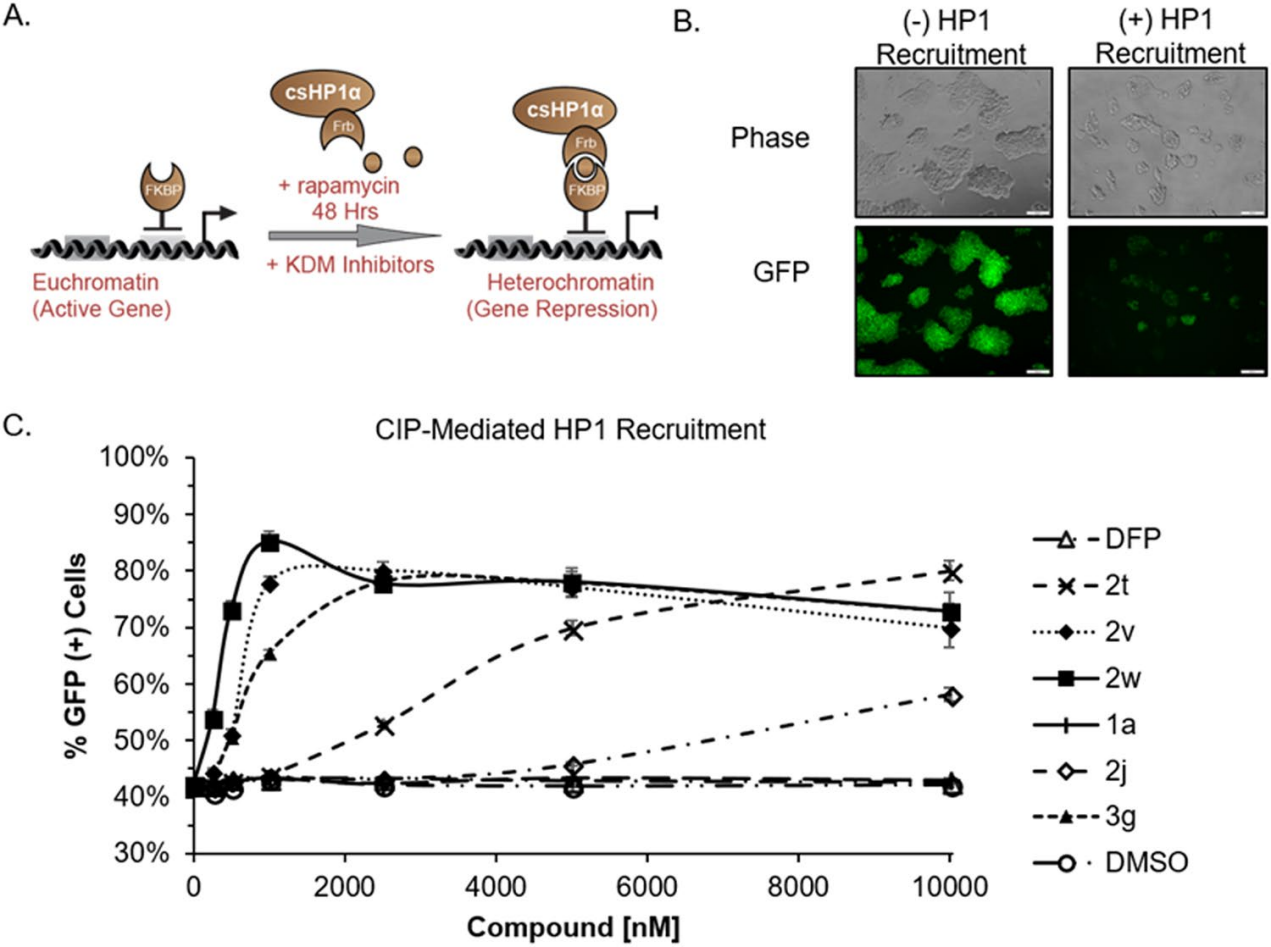
DFP Derivatives Inhibit HP1 Induced Heterochromatin Formation. (**A**) Cartoon schematic of the chromatin *in vivo* assay (CiA) and recruitment of HP1 by CIP-rapamycin addition leads to gene silencing. (**B**) Representative brightfield and GFP images demonstrate the reduction in GFP expression after HP1 recruitment. (**C**) Dose response curves of DFP derivatives after 48 h of HP1 recruitment at 10000, 5000, 2500, 1000, 500, 250, and 0 nM concentrations. Flow cytometry was used to determine the % GFP(+) cells following treatment. n = 6.

To elucidate the effect of DFP-based KDM inhibitors on chromatin dynamics, we profiled them in an intracellular assay which measures heterochromatin formation speed. The chromatin *in vivo* assay (CiA) leverages chemical induced proximity (CIP) to recruit heterochromatin protein 1 (HP1) to a modified *Oct4* locus. This reporter system is composed of tandem arrays of Gal4 and zinc finger DNA binding domains in the promoter of one allele of *Oct4*, where the gene was replaced by a nuclear eGFP reporter. Protein fusions of Gal4-FKBP and FRB-HP1 allow for direct chemical recruitment of HP1 to the *CiA:Oct4* reporter locus upon addition of the chemical inducer of proximity (CIP)-rapamycin. After CIP-rapamycin addition, HP1 is recruited to the reporter locus and brings histone methyltransferase enzymes to the chromatin reporter resulting in the repressive H3K9me3 mark being deposited (Fig. 10A). H3K9me3 deposition is the signature mark of heterochromatin and is coupled with gene silencing and loss of GFP expression (Fig. 10B)^33^. Others have shown that H3K4me3 marked nucleosomes impede H3K9 methyltransferase activity^34^. Thus, we postulate that the active H3K4me3 mark must be removed by KDM enzymes as part of the transformation from euchromatin to heterochromatin. Inhibition of KDMs, specifically those targeting H3K4me3, would result in a decrease in H3K9me3 accumulation, which would be measured in our assay as an increase in GFP positive cells.

To determine the effect of DFP-based KDM inhibitors on HP1-mediated gene repression, we treated *CiA:Oct4* embryonic stem (ES) cells with doses of representative compounds ranging from 10 µM–250 nM for 48 h with CIP-rapamycin recruitment of HP1 (Fig. 10C). For this experiment, we chose representative compounds which displayed potent tumor-selective cytotoxic from each class (**2j**, **2t**, **2v**, **2w** and **3g**), one representative compound which displayed considerably weaker cell cytotoxicity (**1a**) and DFP. The percentage of GFP(+) cells was determined by flow cytometry as indicated in representative histograms (Supplemental Fig. S8). We observed that compounds **2v** and **2w** were the most potent inhibitors of heterochromatin formation reaching max inhibition at 1 µM. Compound **3g** reached maximum inhibition at 2.5 µM while **2t** only reached maximum inhibition of gene repression at 10 µM. **2j** had a 20% increase in the number of GFP positive cells. Compound **1a** and DFP did not inhibit heterochromatin formation.

**Figure 11:**
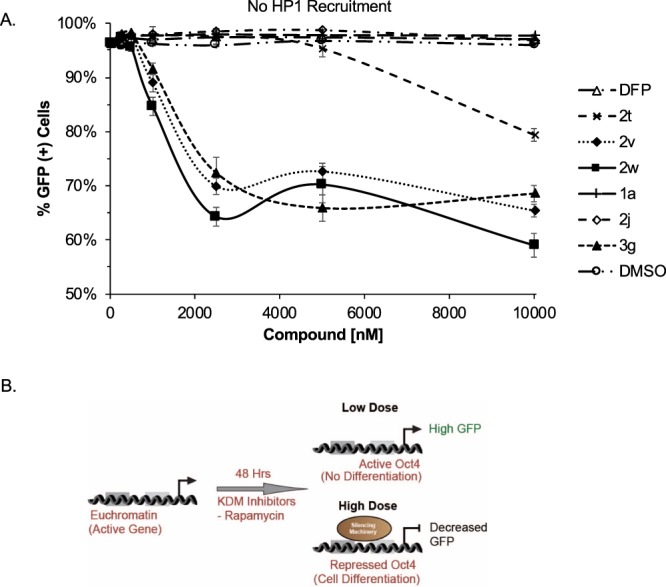
High Concentrations of DFP Derivatives Decrease GFP Expression at Oct4. (**A**) Dose response curves of DFP derivatives after 48 h without HP1 recruitment at 10000, 5000, 2500, 1000, 500, 250, and 0 nM concentrations. (**B**) Cartoon demonstrating the effect of compound concentration on GFP expression in the absence of HP1 recruitment. Flow cytometry was used to determine the % GFP(+) cells following treatment. n = 6.

Embryonic stem cells are sensitive to environmental stimuli which may trigger cell differentiation. During mammalian stem cell differentiation or exposure to toxic compounds, *Oct4* is known to be repressed as cells lose reinforcement of the pluripotency transcriptional activation network^35,36^. Therefore, the *CiA:Oct4* reporter without CIP-recruitment of HP1 functions is a valid system to determine how much cell differentiation or toxicity is occurring by measuring the GFP expression levels. To elucidate the effect of DFP-based KDM inhibitors on Oct4 GFP expression in the absence of HP1 recruitment, we performed dose response studies without addition of CIP-rapamycin (Fig. 11A). Compounds **2v**, **2w**, and **3g** caused decrease in the percentage of GFP(+) cells by ~30% at 2.5 µM while **2t** demonstrated a ~20% decrease in percentage GFP(+) cells only at the 10 µM dose. The remaining compounds showed no change in the percentage of GFP(+) cells. These data indicate that the compounds do not cause cell differentiation or toxicity at the doses tested (Fig. 11B).

### A representative DFP-based KDM inhibitor is efficacious in murine models of ER(+) (T47D) and ER(−) (MDA-MB-231) BCas

**Figure 12:**
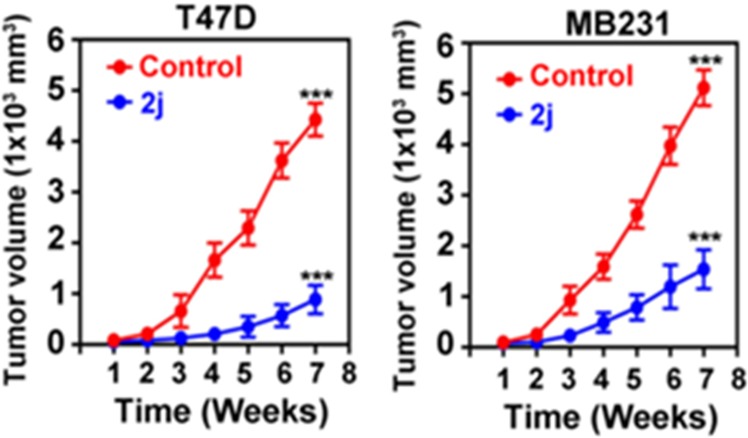
Compound **2j** is efficacious in xenograft mouse models of ER(+) (T47D) and ER(−) (MDA-MB-231) BCas. Mouse xenograft was established using human breast cancer cell lines (T47D and MDA-MB-231) by injecting 1 × 10^7^ cells in 100 μL serum-free medium into mammary fat pads of athymic nude mice and the growth of the tumor was monitored using a caliper. Once tumor size was reached ~250 mm^3^, mice were treated with **2j** (25 mg/kg body weight) for 8 weeks. Tumor volume was measured every week and calculated using the formula (width^2^ × length)/2. *p < 0.05; **p < 0.01 and ***p < 0.001 by two way Annova analysis.

To determine the antitumor potential of the DFP-based KDM inhibitors, we investigated the efficacy of a representative example, **2j**, in two murine xenograft models of BCa. The xenografts were established using two human BCa cell lines – T47D (ER(+)) and MDA-MB-231 (ER(−)). We chose **2j** because it is minimally toxic to normal cells (Table 5 and Fig. 11A) while demonstrating on-target effect (Fig. 10) and cytotoxic to both ER(+) and ER(−) BCa cells (Table 5). Because our in *vitro* study was conducted using MCF-7 as a model of ER(+) cell, we first tested the effect of **2j** on the viability of T47D cells at three concentrations. We observed that **2j** caused a dose-dependent reduction in the growth of T47D cells but to a lesser extent than its effects on the growth of MDA-MB-231 (Fig. S9). The effect of **2j** on T47D viability mirrored that of its effect on MCF-7, confirming preferential cytotoxicity of the DFP-based agents the MDA-MB-231 cells. Once we established that T47D cells are sensitive to **2j**, xenografts of T47D and MDA-MB-231 were established in female athymic nude mice (8-week old) by injection of cells (1 × 10^7^) into the mammary fat pad as described previously^37,38^. After the tumor volume reached ~250 mm^3^, mice were treated with **2j** (25 mg/kg body weight) for 8 weeks. As shown in Fig. 12, treatment with **2j** significantly reduced tumor growth. Relative to control, **2j** caused approx. 81% (4426 vs 884 mm^3^) and 70% (5116 vs 1536 mm^3^) reduction in the growth of T47D and MDA-MBMB-231 tumors, respectively. Overall these results provide clear evidence of the potential of **2j** as a therapeutic agent for BCa regardless of the tumor’s receptor expression status.

## Discussion

DFP, a hydroxypyridinone-derived iron chelator currently in clinical use for iron chelation therapy, elicits antiproliferative activities through mechanisms not clearly understood. The depletion of intracellular iron caused by DFP, the indirect inhibition of DNA synthesis through inactivation of RNR, and reduction of intracellular Zn^2+^ pool, have been suggested to be key contributing factors to the DFP-induced apoptosis^3–9,14^. Hydroxypyridinones, including DFP, form complexes having metal ions to ligand stoichiometry that is dependent on the identity of the chelated metal ion^32^. The diversity of DFP-metal ion complexes and its small, flat aromatic structure could allow DFP access to the active sites of several intracellular metalloenzymes. We have shown in our previous studies that the thiophilicity of zinc enhances the chelation of Zn^2+^ ion at the active site of HDAC6 and HDAC8 to 3-hydroxypyridin-2-thione relative to the closely related hydroxypyridin-2-one, resulting in inhibition of these HDAC isoforms^15,16^. Subfamily of Fe^2+^-dependent KDMs have active site architecture which resembles HDACs’ and may prefer chelation to DFP because of the low thiophilicity of iron.

In the first half of this study, we used a combination of *in silico* molecular docking, cell-free and cell based-assays to investigate the interaction of DFP with KDMs. As shown in Fig. 2, we found that DFP gained access to and formed a bidentate chelate with the Fe^2+^ at the active site of a subset of KDMs. The DFP-KDM interaction is further stabilized by H-bonding between the oxygen moieties of DFP and key residues within the enzymes active sites. This *in silico* observation implicates DFP as a potential inhibitor of the demethylase activity of these KDMs. Subsequent cell-free assay, monitoring KDM enzymatic activity, confirmed our *in silico* prediction. We observed that DFP inhibits six KDMs - 2A, 2B, 5C, 6A, 7A and 7B - at low micromolar IC_50_s. We also found DFP was considerably less active or inactive against eleven KDMs - 1A, 3A, 3B, 4A-E, 5A, 5B and 6B. Results from Western blot analysis (Fig. 4) revealed that DFP caused a dose dependent elevation of H3K4me3 and H3K27me3 levels in MCF-7 cells. This provided evidence for the intracellular KDM inhibition activities of DFP. It is plausible that DFP derives part of its antiproliferative properties from a convergence of KDM inhibition and metal ion chelation, resulting in the perturbation of some intracellular pathways vital to tumor cell growth. DFP elicits KDM inhibition activities at concentrations that are between 7- and 70-fold lower than the iron binding equivalence concentrations at which it inhibits RNR activities and/or reduces labile intracellular zinc ion pool^8,9,14^. Thus, it is highly likely that DFP derives its anti-proliferative activity largely from the inhibition of a sub-set of KDMs identified in this study.

The initial *in silico* docking study on DFP revealed structure-based insights for the optimization of DFP. These *in silico* observations provided a foundation for the SAR study that furnished several novel DFP-based KDM inhibitors which are preferentially more cytotoxic to the TNBC cell line MDA-MB-231. Several members of KDM subfamilies have been implicated in the epigenetic reprograming which sustains BCas regardless of hormone expression status^23,24,39–43^. Additionally, representative BCa cell lines, including MCF-7 and MDA-MB-231, express different levels of KDM subfamilies and elicit distinct sensitivity to other KDM inhibitors^29–31^. However, the basis for the enhanced sensitivity of the TNBC cell line to these DFP-based compounds is not completely clear from this study since we have only screened them against one representative KDM (KDM6A).

We further found that representative compounds **2j**, **2t**, **2v**, **2w** and **3g**, which displayed potent tumor-selective cytotoxic, have intracellular KDM inhibition activities in an orthogonal assay which measures heterochromatin gene repression velocity following HP1 recruitment to the *CiA:Oct4* locus. Here these compounds, significantly inhibited heterochromatin firing at a relevant mammalian locus. We hypothesize this occurs from pan KDM inhibition including H3K4 demethylation which impeded H3K9me3 deposition and gene silencing. Conversely, compound **1a** and the parent DFP compound did not show measurable inhibition activity in the heterochromatin formation assay even at 10 μM. These data are consistent with our findings from the Western blot analysis since increase in H3K4me3 and H3K27me3 levels was observed only at 10x the dose used to measure the heterochromatin formation speed.

Subsequent profiling of a representative compound, **2j**, revealed that the *in vitro* antiproliferative activities displayed by these DFP-based KDM inhibitors are likely going to translate into *in vivo* efficacy as **2j** potently inhibited breast tumor growth in murine xenograft models. Since it is efficacious in representative ER(+) (T47D) and ER(−) (MDA-MB-231) BCas models, **2j** has potential as a novel therapeutic agent for multiple forms of BCas.

In conclusion, our study has furnished a new insight into the mechanism of the anti-proliferative activities of DFP as we revealed that DFP is a pan-selective KDM inhibitor. The docked poses adopted by DFP at KDM active sites also permitted modifications which enabled identification of a cohort of new DFP-based anti-proliferative agents which displayed selective cytotoxicity against a TNBC cell line. Furthermore, this new class of molecules caused a delay in heterochromatin gene repression that was not seen in the parent DFP and a representative compound, **2j**, potently suppressed the growth of T47D and MDA-MBMB-231 in murine xenograft models. Taken together our study has identified a new chemical scaffold capable of inhibiting KDM enzymes, globally changing histone modification profiles, and with specific anti-tumor activities.

## Methods

### Material

Maltol and the phenylacetylene derivatives were purchased from Sigma Aldrich. Anhydrous solvents and other reagents were purchased either from Sigma or Acros and were used without further purification. Analtech silica gel plates (60 F254) were utilized for analytical TLC, and Analtech preparative TLC plates (UV254, 2000 μm) were used for purification. Silica gel (200–400 mesh) was used in column chromatography. TLC plates were visualized using UV light, anisaldehyde, and/or iodine stains. High performance liquid chromatography (HPLC) was performed on an Agilent Technologies 1260 Infinity HPLC system coupled to a Bruker amaZon SL ion trap mass spectrometer operating in positive mode. The system was run using 0.1% formic acid in both water (solvent A) and acetonitrile (solvent B), starting with 5% B for 4 minutes, followed by a gradient increase of 5% to 100% of B over 30 min with flow rate of 0.7 mL/min. All compounds have ≥95% purity as determined by HPLC, except for **3a**–**3f** whose purity ranged from 89–94% (shown for each compound in the protocol). NMR spectra were obtained on a Varian-Gemini 400 MHz and Bruker Ascend™ 500 and 700 MHz magnetic resonance spectrometer. ^1^H NMR spectra were recorded in parts per million (ppm) relative to the residual peaks of CHCl_3_ (7.24 ppm) in CDCl_3_ or CHD_2_OD (4.78 ppm) in CD_3_OD or DMSO-*d*_5_ (2.49 ppm) in DMSO-*d*_6_. MestReNova (version 11.0) was used to process the original “fid” files. High-resolution mass spectra were gathered with the assistance of the Georgia Institute of Technology mass spectrometry facility (Atlanta, GA). Bisazidoalkanes, benzylmaltol, and azidopropyl-hydroxy-methylpyridin-4(1*H*)-one were synthesized adapting literature protocols^44,45^.

### Molecular Docking Analysis

Prior to docking analysis with the appropriate macromolecule (KDM structure) in PyRx, energy minimization of the ligand and merging of non-polar hydrogens were performed in Chem3D 15.1 and Autodock Vina^25^, respectively. After the removal of the heteroatoms, preparations of the macromolecules were performed directly in PyRx where the docking runs were achieved within a 25 Å cube surrounding the active site. Upon the completion of the runs, the results were reported as nine outputs, ranked in accordance to their binding affinity. For molecular interaction analysis, the macromolecule, along with the best fit outputs from PyRx were loaded onto PyMOL where the docked orientations along with molecular interactions were assessed.

### KDM Cell-free Assay

This experiment was done through a contractual agreement with BPS Bioscience, San Diego, CA. Detailed protocol is included in the Supplementary Information.

### Western Blot Analysis for Histone H3 Methylation

Cell lysates were prepared in the RIPA lysis buffer (10 mM Tris-Cl (pH 8.0), 1 mM EDTA, 1% Triton X-100, 0.1% sodium deoxycholate, 0.1% SDS, 140 mM NaCl, and 1 mM PMSF). The images of the Western blots were scanned by LI-COR’s Odyssey^®^ (LI-COR) and the signal intensities were quantified using Image Studio^™^ software (LI-COR) (Fig. S10). More details are provided in the Supplementary Information.

### Cell Viability Assay

The *In vitro* proliferation assay was performed as reported previously^15,16^. Detailed description of the protocol used is included in the Supplementary Information.

### HP1-Recruitment Assay Dose Curve

Day 0, *CiA:Oct4* N118/163 cells were grown in ES media and seeded at a density of 10,000 cells per well in 100 μL media (100,000 cells mL^−1^) on gelatin coated 96 well plates. Day 1, culture media was aspirated and replaced with 100 µL ES media containing +/− 6 nM rapamycin. 10 µM, 5 µM, 2.5 µM, 1 µM, 500 nM, and 250 nM concentrations of compound or DMSO were added using a TTP Labtech Mosquito HTS liquid handler. Days 2, fresh ES media +/− rapamycin and compound were added as in Day 1. Day 3, cells were washed with PBS and trypsinized using 0.25% trypsin-EDTA. Trypsin was quenched with serum. Cells were resuspended by pipetting in preparation for flow cytometry analysis.

### Flow Cytometry and Analysis

Flow cytometry data was acquired using the Intellicyt iQue Screener and analyzed with FlowJo software. More details are provided in the Supplementary Information.

### Mouse Xenograft Assay

Female athymic nude mice (8-week old) were purchased from Jackson Laboratories and housed in standard conditions. T47D and MDA-MBMB231 cells were injected subcutaneously into the mammary fat pad (1 × 10^7^ cells in 100 μL of serum-free medium) as described previously^37,38^. Tumor size was measured periodically by caliper, and tumor volume was calculated using the formula (width^2^ × length)/^2^. Once the tumor volume reached ~250 mm^3^, mice were treated with **2j** (25 mg/kg body weight) for 8 weeks. The control and the treatment groups comprised of 5 mice per group and the experiment was repeated 2 times (n = 10). During the course of the experiment, tumor volume was measured every week and values are expressed as mean ± SD. All animal experiments were approved by the Augusta University Institutional Animal Care and Use Committee. All protocols were conducted in accordance with the Augusta University Institutional Animal Care and Use Committee.

## Supplementary information

Supplementary Information
