# Supplementary material for: Deferiprone: Pan-selective Histone Lysine Demethylase Inhibition Activity and Structure Activity Relationship Study
Source: Sci Rep. 2019 Mar 18;9:4802. doi: 10.1038/s41598-019-39214-1 (PMC6423038; doi:10.1038/s41598-019-39214-1)

**Deferiprone: Pan-selective Histone Lysine Demethylase Inhibition Activity and Structure Activity Relationship Study**

Verjine Khodaverdian^1^, Subhasish Tapadar^1†^, Ian A. MacDonald^2†^, Yuan Xu^1^, Po-Yi Ho^3^, Allison Bridges^4^, Pragya Rajpurohit^4^, Bhakti A Sanghani^3^, Yuhong Fan^3, 5^, Muthusamy Thangaraju^4^, Nathaniel A. Hathaway^2^ and Adegboyega K. Oyelere ^1, 5^

*School of Chemistry and Biochemistry, School of Biological Sciences, Parker H. Petit Institute for Bioengineering and Bioscience, Georgia Institute of Technology, Atlanta, GA 30332-0400 USA; Department of Biochemistry and Molecular Biology, Medical College of Georgia, Augusta University, Augusta, GA 30912, USA; and The University of North Carolina Eshelman School of Pharmacy, Chapel Hill, NC 27599 USA*

^1^School of Chemistry and Biochemistry, Georgia Institute of Technology

^2^The University of North Carolina Eshelman School of Pharmacy

^3^School of Biological Sciences, Georgia Institute of Technology

^4^Medical College of Georgia, Augusta University

^5^Parker H. Petit Institute for Bioengineering and Bioscience, Georgia Institute of Technology

^†^These Authors contributed equally to the manuscript

**Correspondence to:**

Adegboyega K. Oyelere, **E-mail:** [aoyelere@gatech.edu](mailto:aoyelere@gatech.edu)

Nathaniel A. Hathaway, **E-mail:** natehat@email.unc.edu

**Keywords:** Deferiprone, bidentate iron chelator, histone lysine demethylases, molecular docking, H3K4me3, H3K27me3

**Table of Contents**

[Supplementary Figures S2](#_Toc510108204)-S10

[Chemistry S10](#_Toc510108205)-S37

Supplementary Experimental ……………………………………………………………..S38-S42

[^1^H and ^13^C Spectra of 1a – f, 2a – w, and 3a – g S42-S76](#_Toc510108206)

Supplementary Figures


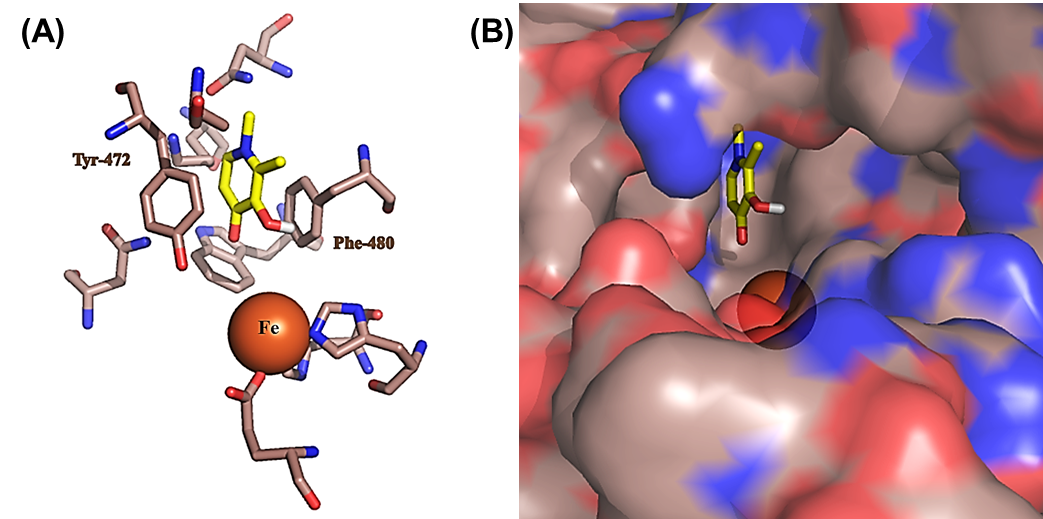


**Fig S1: The low energy docked orientation of DFP at the active site of KDM5A**  (PDB: 5CEH) [1] – ball and stick (a) and surface presentation (b) models – revealed that DFP is too far from the Fe^2+^ for a strong chelation although it is stabilized by a π-π interaction with Phe 480.


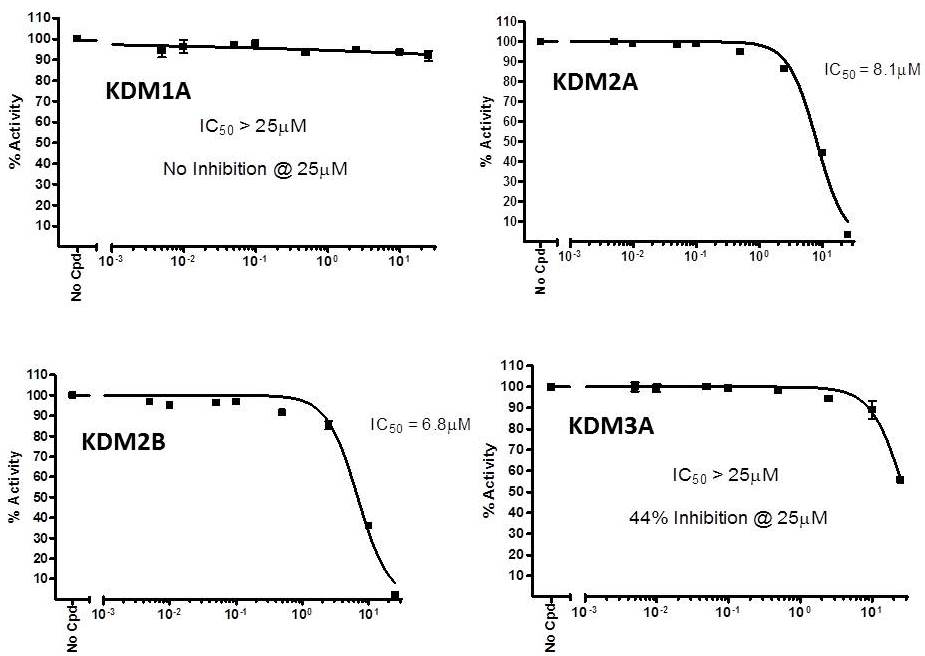


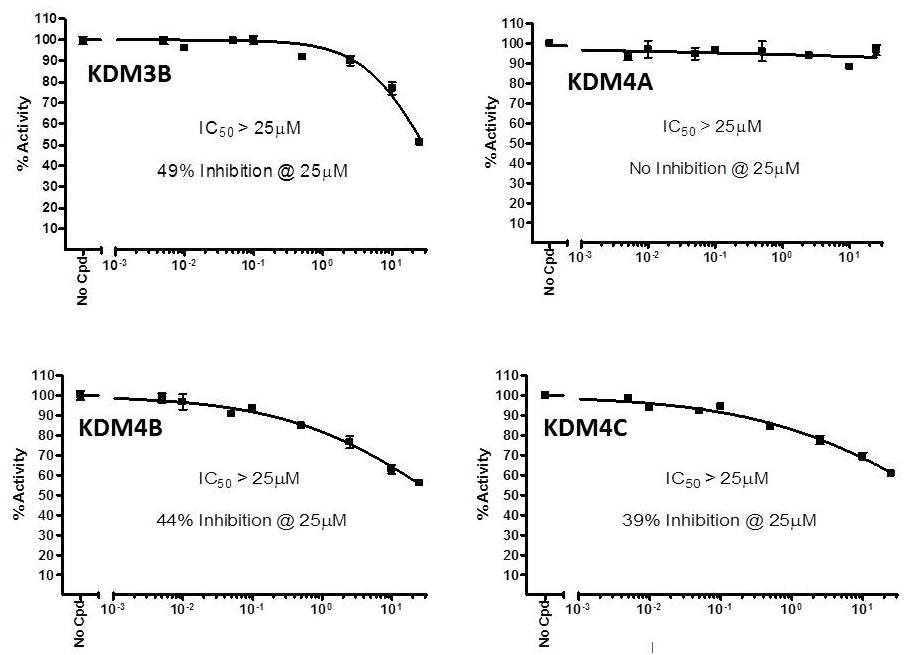


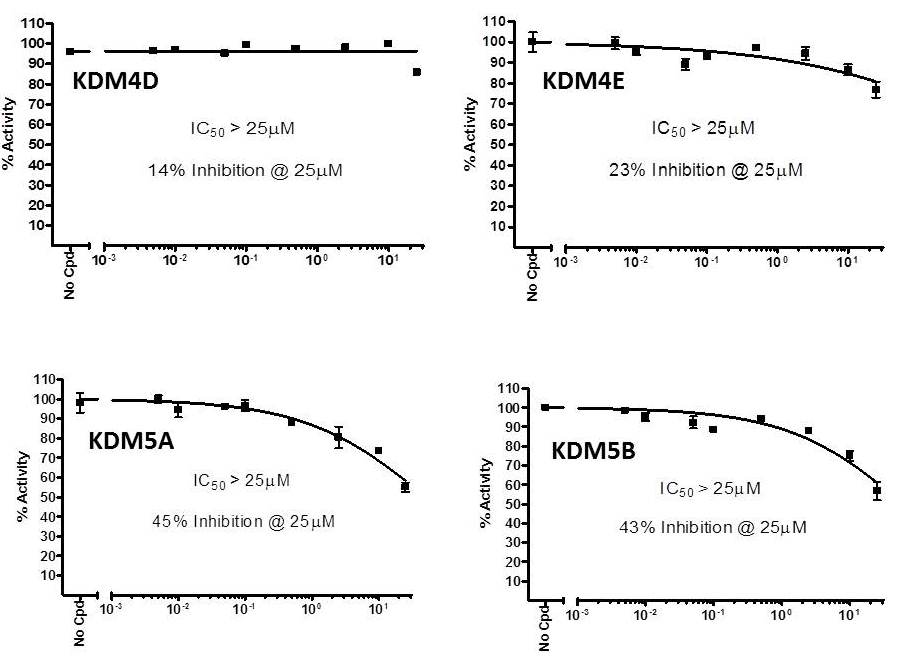


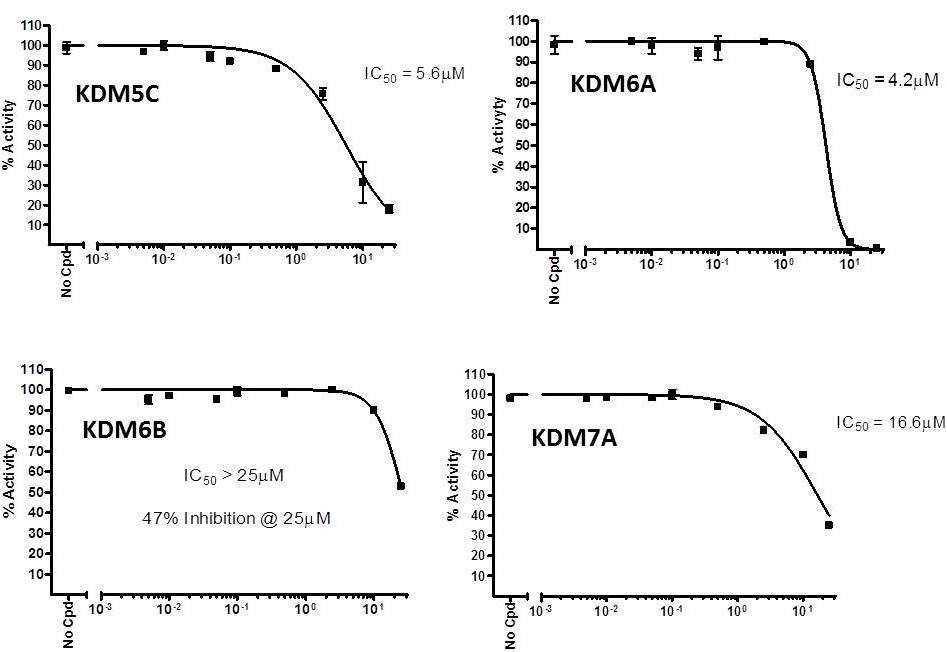


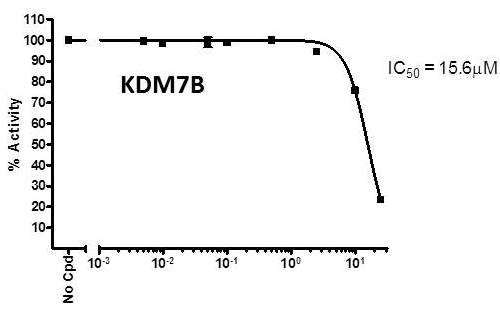


**Fig S2: Dose response curve of KDMs inhibition by DFP.**

**
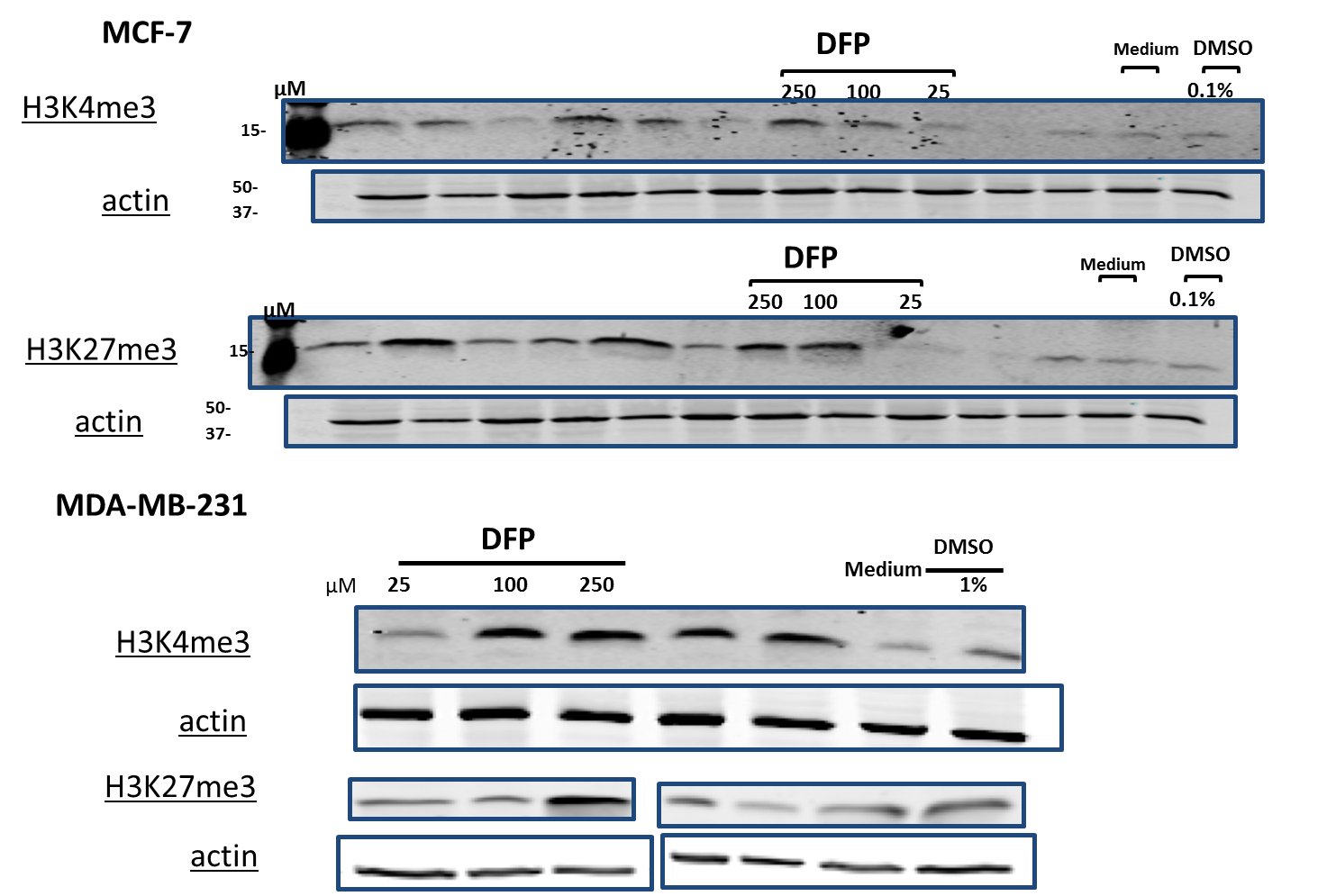
**

**Fig S3: Representative full-length gels of the data gels presented in Fig 4.** Blotting for the histone methylation and actin expression status were performed on the same gel.

**Fig S4: Dose response curve of cell proliferation inhibition by DFP on four cell lines.** MDA-MB-231 (red), MCF-7 (light gray), VERO (gray), and DU-145 (black).

**
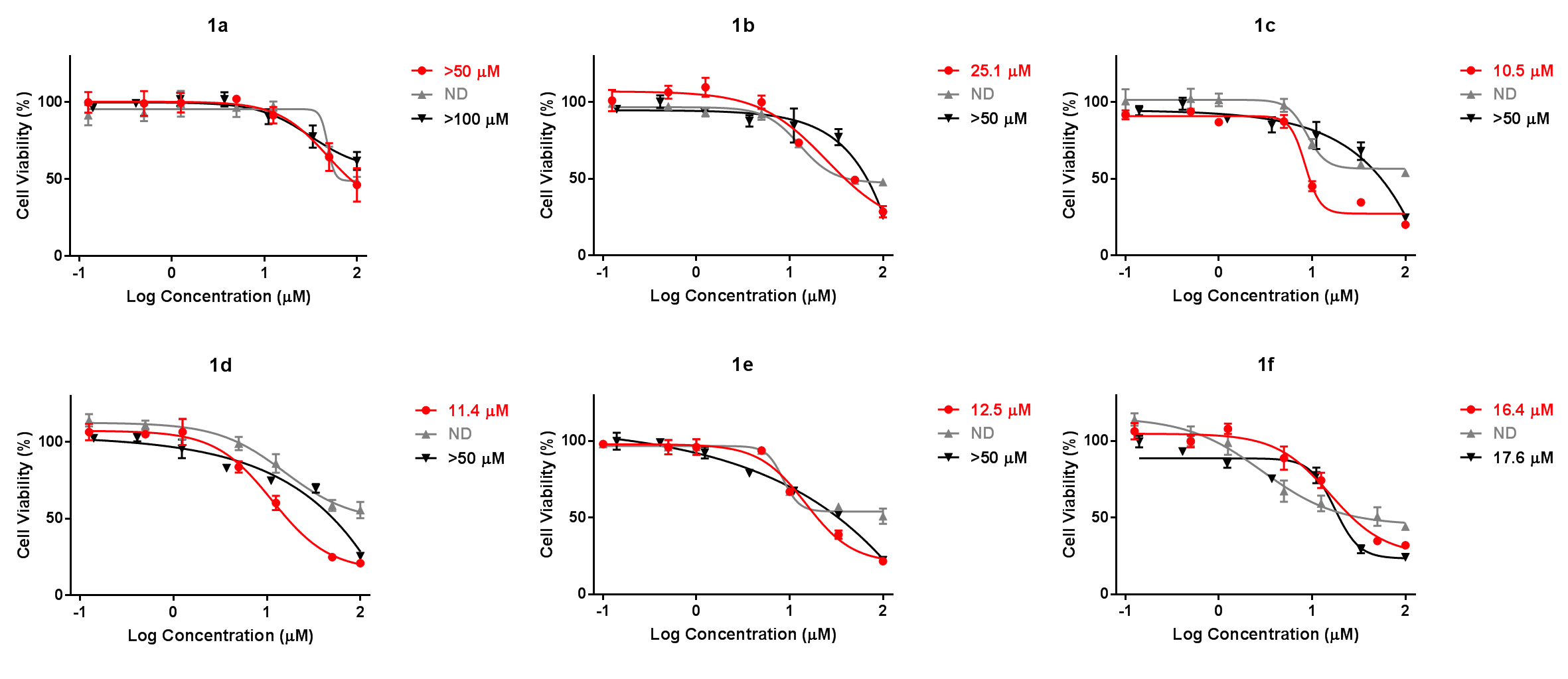
**

**Fig S5: Dose response curve of cell proliferation inhibition by 1a – f on three cell lines.** MDA-MB-231 (red), MCF-7 (gray), and VERO (black).


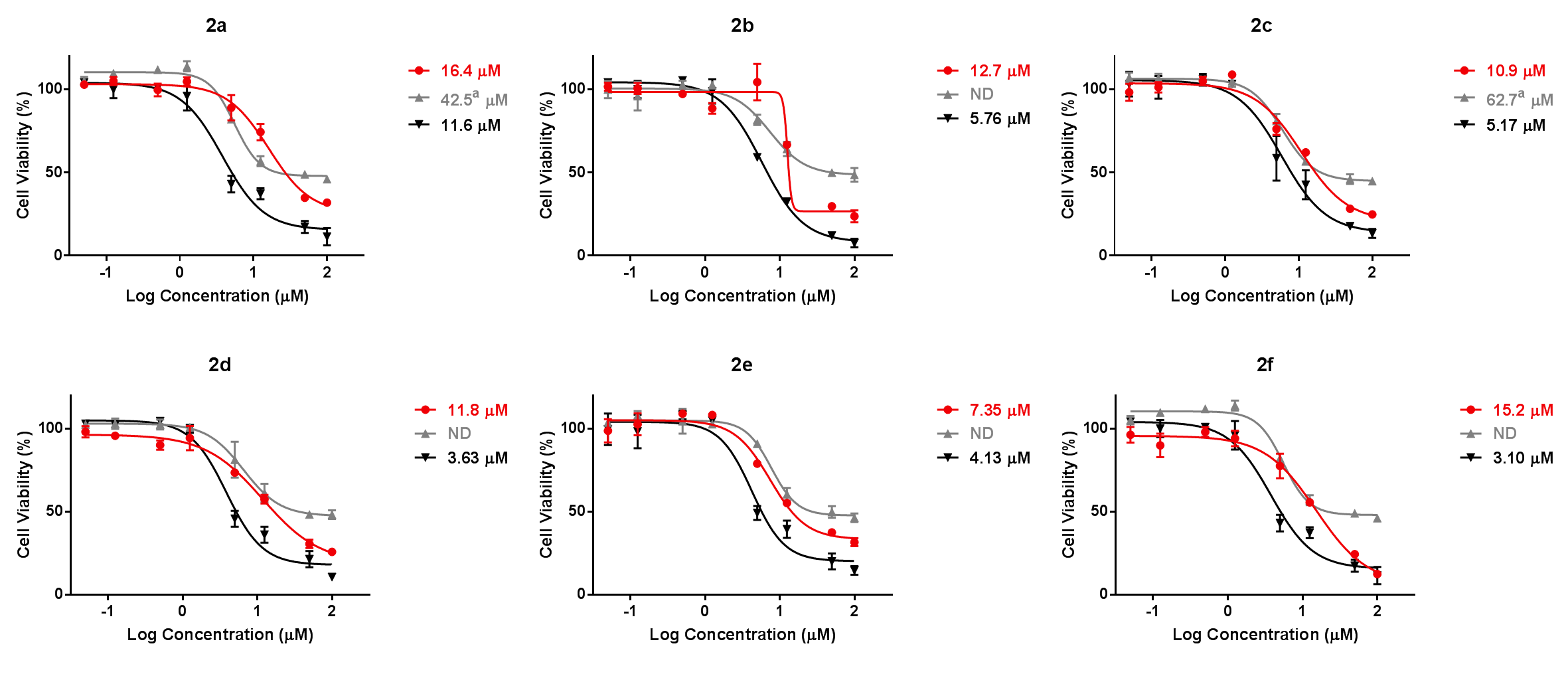

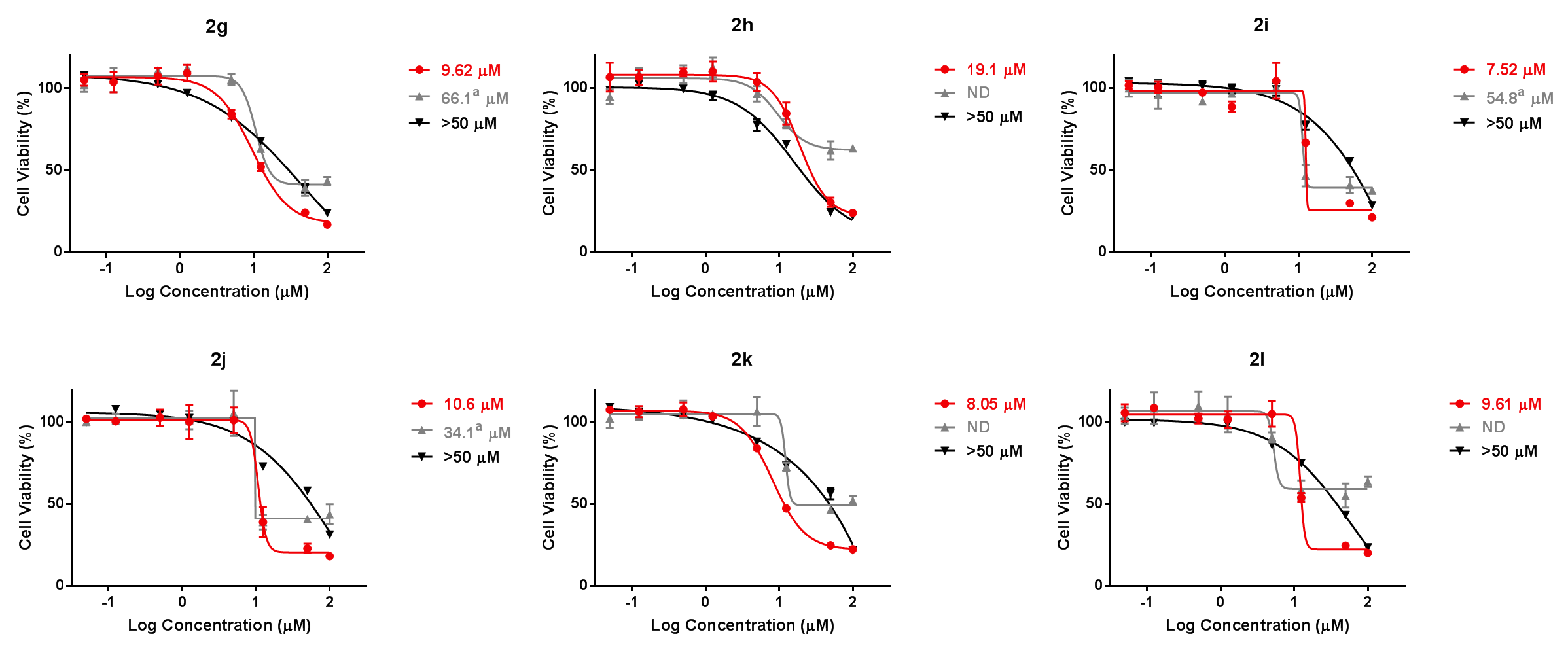


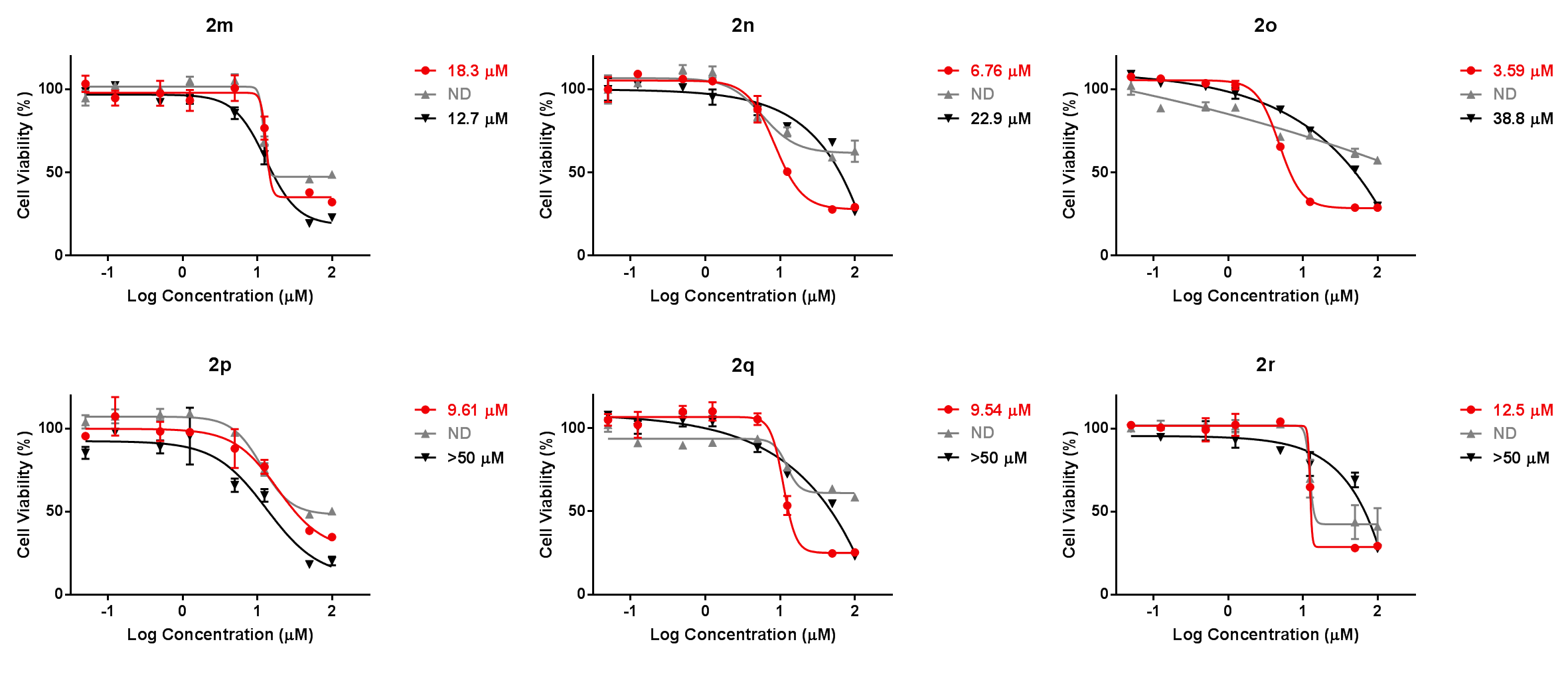


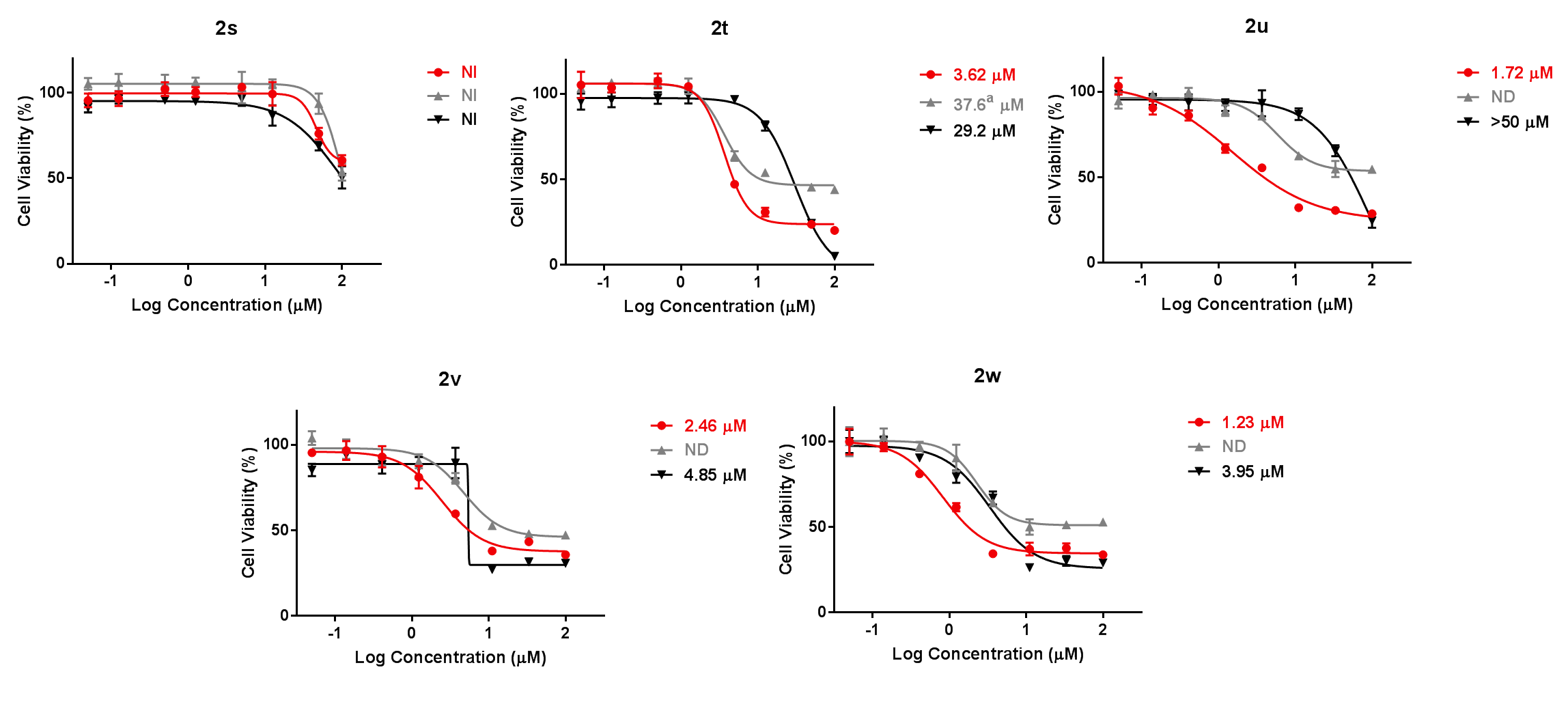


**Fig S6: Dose response curve of cell proliferation inhibition by 2a – w on three cell lines.** MDA-MB-231 (red), MCF-7 (gray), and VERO (black). ^a^ The reported values for MCF-7 cell line was calculated by linear regression.


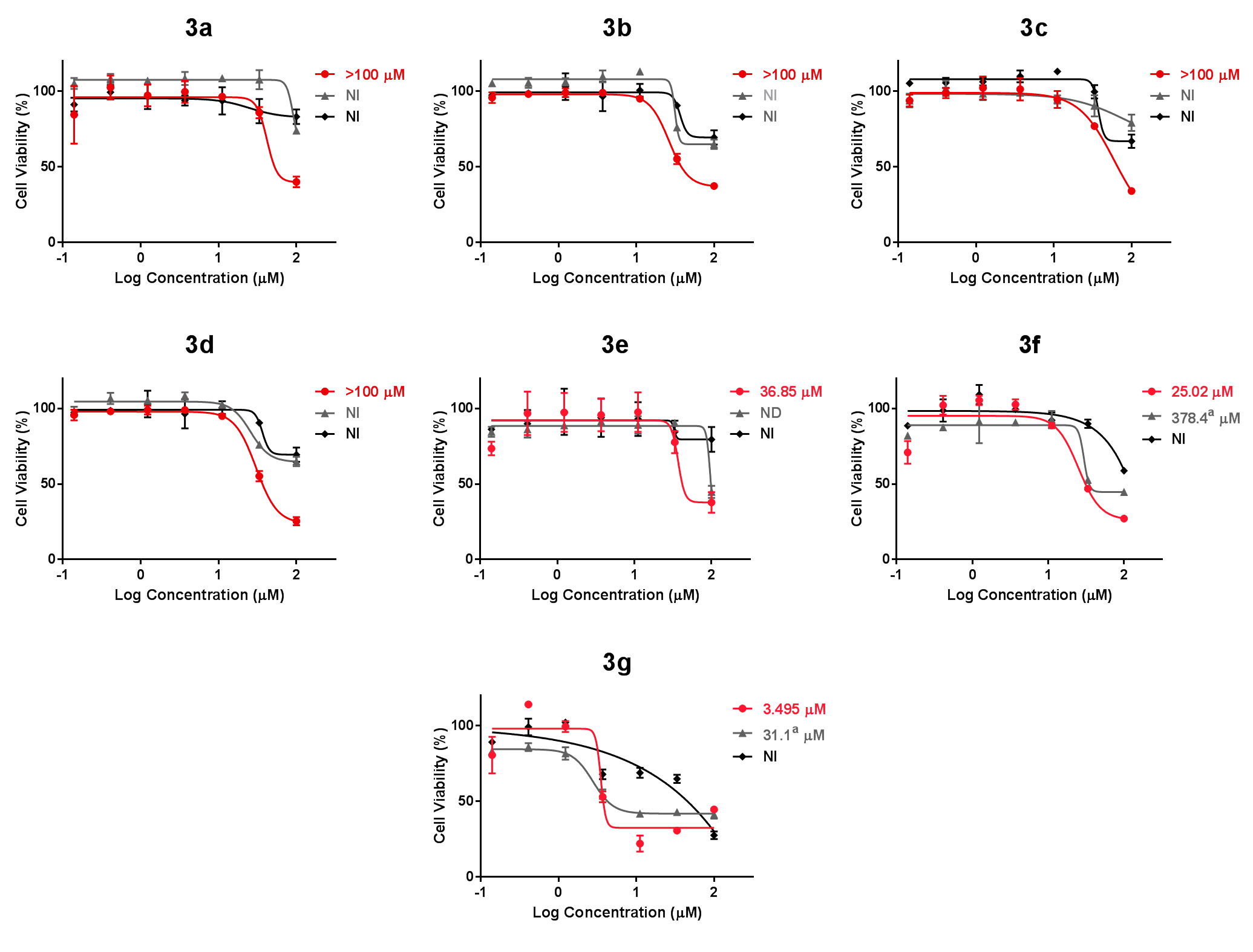


**Fig S7: Dose response curve of cell proliferation inhibition by 3a – g on three cell lines.** MDA-MB-231 (red), MCF-7 (gray), and VERO (black). ^a^ The reported values for MCF-7 cell line was calculated by linear regression.


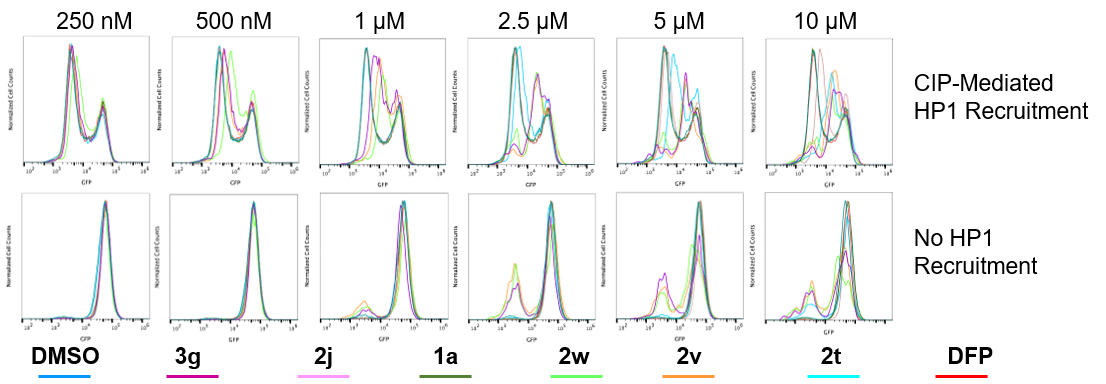


**Fig S8: Flow Cytometry Histograms Demonstrate DFP Derivatives Inhibit HP1 Induced Heterochromatin Formation in a Dose Dependent Manner.** Top, Histograms of cells analyzed for GFP expression, where HP1 was directed to the *CiA:Oct4* allele for 2 days by addition of CIP-rapamycin at the same time as with the indicated small molecule at the indicated concentration. Bottom, control experiment histogram of cells analyzed for GFP expression in the absence of HP1 recruitment with the indicated small molecule and concentration.


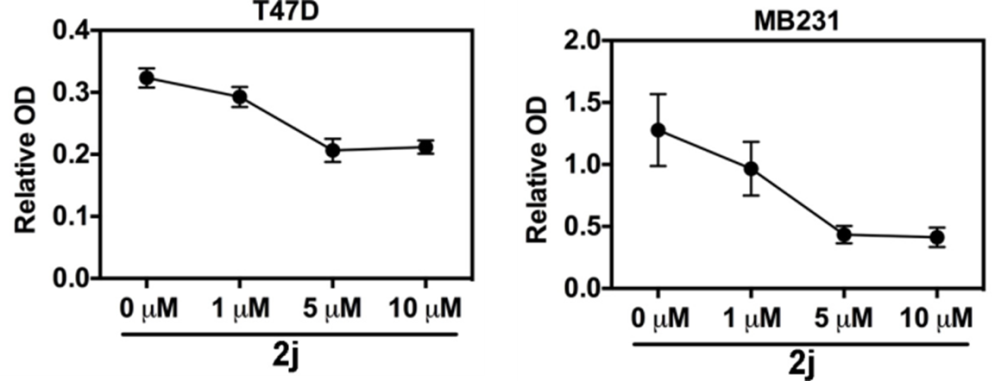


**Fig S9: Compound 2j caused a dose-dependent inhibition of T47D cells growth but to a lesser extent than its effects on the growth of MDA-MB-231.**


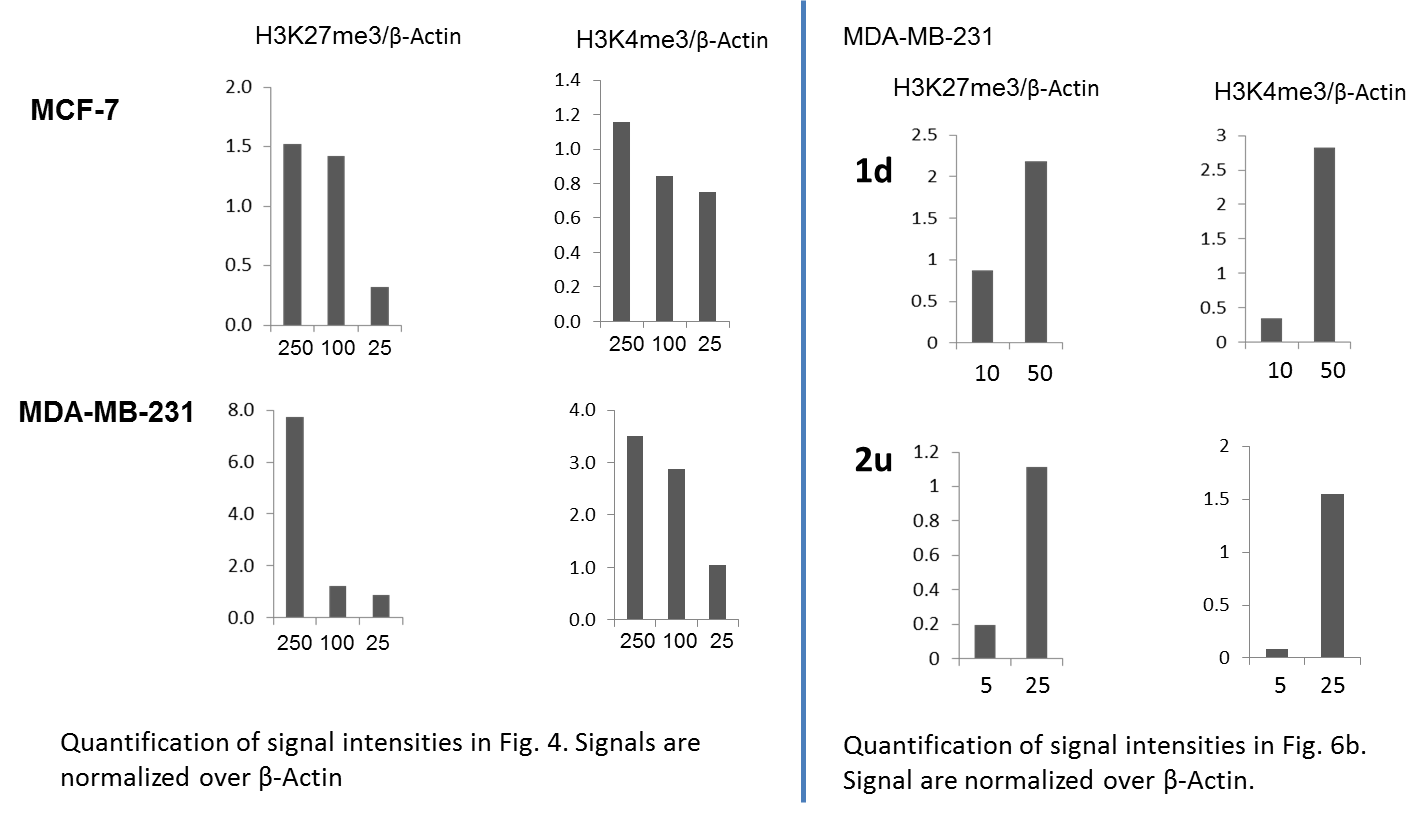


**Fig S10: Quantified Western blots signal intensities using Image Studio™ software (LI-COR).**

# Chemistry

General: Bisbromoalkane and sodium azide in dimethylformamide (DMF) were reacted at 80 ˚C for 24 h to obtain compounds **5a – d**. Molecules **4a – f** were synthesized using two different protocols. For shorter alkane chains (**4a** and **b;** n = 1 and 2), bromoalkylamine hydrobromide and sodium azide in water were heated at 80 ˚C for 12 – 18 h in which KOH was added and extracted with ether. Compound **4c – f** were synthesized using the triphenylphosphine (PPh_3_) reduction of compound **5a – d** in 5% HCl and 1:1 ethyl acetate (EtOAc):ether at 0 ˚C – room temperature for 24 h.

2-Azidoethan-1-amine (**4a**). Mixture of bromoethylamine hydrobromide **31** (1.5 g, 7.3 mmol) and sodium azide (1.63 g, 24.2 mmol) in water (20 mL) was refluxed at 80 ˚C for 12 – 18 h in which KOH and ether (30 mL) were added to the reaction mixture on an ice bath. The organic layer was separated and the aqueous layer was extracted with ether (2 x 30 mL). The combined organic layers were dried over K_2_CO_3_ and concentrated *in vacuo* to result a clear colorless oil (384 mg, 61%). ^1^H NMR (400 MHz, CDCl_3_) *δ* 3.34 (t, *J* = 6.7 Hz, 2H), 2.85 (t, *J* = 6.9 Hz 2H), 1.30 (s, 2H).

3-Azidopropan-1-amine (**4b**). Bromopropylamine hydrobromide **32** (3.31 g, 15.1 mmol) and sodium azide (2.9 g, 45. mmol) in water (35 mL) were subjected to the conditions described for compound **4a** to obtain clear colorless oil (1.2 g , 82% ). ^1^H NMR (400 MHz, CDCl_3_) *δ* 3.33 (t, *J* = 6.7 Hz, 2H), 2.76 (td, *J* = 6.9, 1.8 Hz, 2H), 1.74 – 1.63 (m, 2H), 1.50 (s, 2H).

4-Azidobutan-1-amine (**4c**). To a stirred solution of 1,4-diazidobutane **5a** (1.50 g, 10.7 mmol) in a mixture of EtOAc (10 mL), Et_2_O (10 mL) and 5% HCl solution (20 mL), PPh_3_ (2.67 g, 10.2 mmol) were added in sequence at 0 ˚C for 1 h. The reaction mixture was then stirred at room temperature for another 24 h. After that aqueous layer was separated and was washed with dichloromethane (DCM) (3 x 30 mL). Aqueous layer was made basic with concentrated NaOH solution and extracted with DCM (3 x 30 mL), dried over Na_2_SO_4_, and concentrated to afford **4c** as light yellow oil (0.85 g, 70%). ^1^H NMR (400 MHz, CDCl_3_) *δ* 3.25 (t, *J* = 6.8 Hz, 2H), 2.68 (t, *J* = 7.0 Hz, 2H), 1.66 – 1.55 (m, 2H), 1.53 – 1.44 (m, 2H), 1.21 (s, 2H).

4-Azidopentan-1-amine (**4d**). Reaction of 1,5-diazidopentane **5b** (2.47 g, 16.2 mmol) and PPh_3_ (4.06 g, 15.4 mmol), using the same reaction condition described for the synthesis of **4c**, furnished **4d** (1.24 g, 61%) as yellow oil. ^1^H NMR (400 MHz, CDCl_3_) *δ* 3.25 (t, *J* = 6.9 Hz, 2H), 2.68 (t, *J* = 6.7 Hz, 2H), 1.59 (dt, *J* = 14.2, 7.0 Hz, 2H), 1.50 – 1.35 (m, 4H), 1.18 (s, 2H).

6-Azidohexan-1-amine (**4e**). Reaction of 1,6-diazidohexane **5c** (1.97 g, 11.8 mmol) and PPh_3_ (3.01 g, 11.4 mmol), using the same reaction condition described for the synthesis of **4c**, furnished **4e** (1.24 g, 61%) as yellow oil(1.35g, 70%). ^1^H NMR (400 MHz, CDCl_3_) *δ* 3.20 (q, *J* = 7.0 Hz, 2H), 2.66 (s, 2H), 1.60 – 1.51 (m, 2H), 1.49 – 1.39 (m, 2H), 1.39 – 1.24 (m, 4H).

7-Azidoheptan-1-amine (**4f**). Reaction of 1,7-diazidoheptane **5d** (1.0 g, 5.3 mmol) and PPh_3_ (1.3 g, 5.1 mmol), using the same reaction condition described for the synthesis of **4c**, furnished **4f** (0.63g, 73%). as yellow oil. ^1^H NMR (400 MHz, CDCl_3_) *δ* 3.20 (t, *J* = 6.9 Hz, 2H), 2.63 (t, *J* = 7.0 Hz, 2H), 1.60 – 1.49 (m, 2H), 1.43 – 1.24 (m, 8H), 1.19 (s, 2H).

1,4-Diazidobutane (**5a**). To the suspension of NaN_3_ (1.89 g, 28.9 mmol) in DMF (20 mL) was added 1,4-dibromobutane (2.52 g, 11.5 mmol), and the mixture was heated to 80 ˚C for 12 – 18 h. Upon cooling the reaction, water (80 mL) was added and the product was extracted with ether (3 x 30 mL). The combined organic layer was washed with adequate water to ensure removal of DMF, and it was dried over K_2_CO_3_ and concentrated to furnish compound **5a** as colorless oil (1.9 g, 91%). ^1^H NMR (400 MHz, CDCl_3_) *δ* 3.35 – 3.27 (m, 4H), 1.69 – 1.64 (m, 4H).

1,5-Diazidopentane (**5b**). Reaction of 1,5-dibromopentane (5.91 g, 25.7 mmol) and NaN_3_ (4.22 g, 64. 2 mmol), using the same reaction condition described for the synthesis of **5a**, gave **5b** (3.7 g, 93%) as colorless oil. ^1^H NMR (400 MHz, CDCl_3_) *δ* 3.28 (t, *J* = 6.8 Hz, 2H), 1.62 (ddd, *J* = 12.0, 10.5, 7.2 Hz, 2H), 1.51 – 1.42 (m, 1H).

1,6-Diazidohexane (**5c**). Reaction of 1,6-dibromohexane (2.53 g, 10.2 mmol) and NaN_3_ (1.67 g, 25.6 mmol) using the same reaction condition described for the synthesis of **5a**, gave **5c** (1.6 g, 95%) as colorless oil. ^1^H NMR (400 MHz, CDCl_3_) *δ* 3.26 (t, *J* = 6.9 Hz, 4H), 1.65 – 1.52 (m, 4H), 1.44 – 1.33 (m, 4H).

1,7-Diazidoheptane (**5d**). Reaction of 1,7-dibromoheptane (1.5 g, 5.8 mmol) and NaN_3_ (0.95 g, 14.5 mmol) using the same reaction condition described for the synthesis of **5a**, gave **5d** (1.0 g, 92%). ^1^H NMR (400 MHz, CDCl_3_) δ 3.25 (t, *J* = 6.9 Hz, 4H), 1.63 – 1.55 (m, 4H), 1.44 – 1.31 (m, 6H).

3-(Benzyloxy)-2-methyl-4*H*-pyran-4-one (benzylmaltol; **7**). Benzyl chloride (4.11 mL, 35.7 mmol) was added dropwise at room temperature to a stirring suspension of 3-hydroxy-2-methylpyranone (maltol; 2.25 g, 17.8 mmol) and anhydride K_2_CO_3_ (4.39 g, 35.7 mmol) in DMF (14 mL), and the mixture was heated at 75 ˚C overnight. After addition of cold water (80 mL) to the reaction mixture, it was extracted with DCM (3 x 80 mL). The combined organic layer was washed with water (2 x 50 mL), dried over Na_2_SO_4_, and concentrated *in vacuo*. The crude was purified by column chromatography (EtOAc: hexane 4:3) to afford the title compound **7** (3.4 g, 89%) as bright yellow oil. ^1^H NMR (400 MHz, CDCl_3_) *δ* 7.60 (d, *J* = 5.6 Hz, 1H), 7.42 – 7.28 (m, 6H), 6.40 (m, *J* = 5.6 Hz, 1H), 5.14 (s, 2H), 2.09 – 2.04 (m, 3H).

1-(2-Azidoethyl)-3-(benzyloxy)-2-methylpyridin-4(1*H*)-one (**8a**). 2-azidoethan-1-amine **4a** (67.7 mg, 0.786 mmol) was added to the solution of benzylmaltol **7** (204 mg, 0.943 mmol) in mixture of ethanol:water (3:2) followed by addition of catalytic amount of NaOH (15.1 mg, 0.377 mmol) in sealed tube, and the mixture was heated at 110 ˚C for 72 h. Water was added to the mixture and extracted with DCM (3 x 30 mL), dried over Na_2_SO_4_, and concentrated. Purification with column chromatography (10% MeOH in CHCl_3_) yielded title compound **8a** as brown oil (124 mg, 55.6%). ^1^H NMR (400 MHz, CD3OD) *δ* 8.31 (d, *J* = 7.2 Hz, 1H), 7.47 – 7.33 (m, 5H), 7.21 (d, *J* = 7.2 Hz, 1H), 5.20 (s, 2H), 4.51 – 4.45 (m, 2H), 3.87 – 3.79 (m, 2H), 2.49 (s, 3H).

1-(3-Azidopropyl)-3-(benzyloxy)-2-methylpyridin-4(1*H*)-one (**8b**). Reaction of 3-azidopropan-1-amine **4b** (123 mg, 1.23 mmol) and benzylmaltol (210 mg, 0.97 mmol), using the same reaction condition described for the synthesis of **8a**, gave **8b** (200 mg, 60%) as brown oil. ^1^H NMR (400 MHz, MeOH-*d*_4_) *δ* 8.37 (dd, *J* = 10.1, 9.1 Hz, 1H), 7.45 – 7.31 (m, 5H), 7.26 (d, *J* = 7.1 Hz, 1H), 5.18 (d, *J* = 10.5 Hz, 2H), 4.41 (dd, *J* = 17.0, 9.5 Hz, 2H), 3.45 (t, *J* = 9.5 Hz ,2H), 2.50 (s, 3H), 2.07 – 1.98 (m, 2H).

1-(4-Azidobutyl)-3-(benzyloxy)-2-methylpyridin-4(1*H*)-one (**8c**). Reaction of 4-azidobutan-1-amine **4c** (831 mg, 7.31 mmol) and benzylmaltol (1.90 g, 8.76 mmol), using the same reaction condition described for the synthesis of **8a**, gave **8c** (1.3 g, 56%) as brown oil. ^1^H NMR (400 MHz, CDCl_3_) *δ* 7.40 – 7.35 (m, 2H), 7.33 – 7.27 (m, 3H), 7.21 (d, *J* = 7.5 Hz, 1H), 6.41 (d, *J* = 7.5 Hz, 1H), 5.19 (s, 2H), 3.80 – 3.74 (t, *J* = 7.6 Hz, 2H), 3.30 (t, *J* = 6.5 Hz, 2H), 2.06 (s, 3H), 1.69 (ddd, *J* = 15.2, 11.3, 7.5 Hz, 2H), 1.57 – 1.47 (m, 2H).

1-(5-Azidopentyl)-3-(benzyloxy)-2-methylpyridin-4(1*H*)-one (**8d**). Reaction of 5-azidopentan-1-amine **4d** (818 mg, 6.38 mmol) and benzylmaltol (1.66 g, 7.66 mmol), using the same reaction condition described for the synthesis of **8a**, gave **8d** (1.3 g, 61%) as brown oil. ^1^H NMR (400 MHz, CDCl_3_) *δ* 7.38 (ddd, *J* = 8.4, 4.7, 3.0 Hz, 2H), 7.37 – 7.27 (m, 3H), 7.16 (d, *J* = 8.2, 1H), 6.40 (d, *J* = 7.5 Hz, 1H), 5.21 (s, 2H), 3.72 (t, *J* = 7.4, 2H), 3.28 (t, *J* = 6.6 Hz, 2H), 2.06 (s, 3H), 1.61 (ddt, *J* = 21.6, 14.7, 7.2 Hz, 4H), 1.35 (tt, *J* = 10.5, 6.2 Hz, 2H).

1-(6-Azidohexyl)-3-(benzyloxy)-2-methylpyridin-4(1*H*)-one (**8e**). Reaction of 5-azidohexan-1-amine **4e** (379 mg, 2.67 mmol) and benzylmaltol (980. mg, 4.53 mmol), using the same reaction condition described for the synthesis of **8a**, gave **8e** (0.48 g, 53%) as brown oil. ^1^H NMR (400 MHz, CDCl_3_) *δ* 7.36 (ddd, *J* = 12.3, 7.3, 1.8 Hz, 5H), 7.17 (d, *J* = 7.3 Hz, 1H), 6.44 (d, *J* = 7.5 Hz, 1H), 5.23 (s, 2H), 3.78 – 3.68 (t, *J* = 6.9 Hz , 2H), 3.27 (t, *J* = 6.7 Hz, 2H), 2.08 (s, 3H), 1.60 (ddd, *J* = 21.4, 14.7, 7.1 Hz, 4H), 1.35 (dd, *J* = 29.9, 7.6 Hz, 4H).

1-(2-Azidoethyl)-3-(hydroxy)-2-methylpyridin-4(1*H*)-one (**9a**). Compound **8a** (124 mg, 0.436 mmol) was suspended in concentrated HCl (6 mL) at room temperature and the suspension was stirred for another 6 h during which the reaction mixture became homogeneous. The HCl was evaporated off under vacuum after a near consumption of **8a** was observed by TLC (10% MeOH in CHCl_3_) to afford compound **9a** light brown solid (76 mg, 90%). ^1^H NMR (400 MHz, MeOH-*d*_4_) *δ* 8.15 (d, *J* = 7.0 Hz, 1H), 7.14 (d, *J* = 7.0 Hz, 1H), 4.60 – 4.53 (m, 2H), 3.93 – 3.87 (m, 2H), 2.66 (s, 3H).

1-(3-Azidopropyl)-3-(hydroxy)-2-methylpyridin-4(1*H*)-one (**9b**). Compound **8b** (200 mg, 0.664 mmol) was subjected to the same reaction condition as described for the synthesis of **9a** to give **9b** (130 mg, 92%) as brown solid. ^1^H NMR (400 MHz, MeOH-*d*_4_) *δ* 8.20 (d, *J* = 7.0 Hz, 1H), 7.17 (d, *J* = 7.0 Hz, 1H), 4.50 (t, *J* = 7.5 Hz, 2H), 3.51 (t, *J* = 6.3 Hz, 2H), 2.65 (s, 3H), 2.18 – 2.04 (m, 2H).

1-(4-Azidobutyl)-3-(hydroxy)-2-methylpyridin-4(1*H*)-one (**9c**). Compound **8c** (13 g, 4.1 mmol) was subjected to the same reaction condition as described for the synthesis of **9a** to give **9c** (861 mg, 95%) as brown solid. ^1^H NMR (400 MHz, MeOH-*d*_4_) *δ* 8.18 (d, *J* = 7.0 Hz, 1H), 7.13 (d, *J* = 6.9 Hz, 1H), 4.41 (t, *J* = 6.9 Hz, 2H), 3.41 (t, *J* = 6.6 Hz, 2H), 2.64 (s, 3H), 1.98 – 1.87 (m, 2H), 1.72 – 1.63 (m, 3H).

1-(5-Azidopentyl)-3-(hydroxy)-2-methylpyridin-4(1*H*)-one (**9d**). Compound **8d** (1.3 g, 3.9 mmol) was subjected to the same reaction condition as described for the synthesis of **9a** to give **9d** (850 mg, 93%) as brown solid. ^1^H NMR (400 MHz, MeOH-*d*_4_) *δ* 8.20 (d, *J* = 6.7 Hz, 1H), 7.15 (d, *J* = 6.6 Hz, 1H), 4.45 – 4.29 (m, 2H), 3.37 – 3.25 (m, 2H), 2.64 (s, 3H), 1.98 – 1.81 (m, 2H), 1.72 – 1.59 (m, 2H), 1.48 (dt, *J* = 9.9, 7.7 Hz, 2H).

1-(6-Azidohexyl)-3-(hydroxy)-2-methylpyridin-4(1*H*)-one (**9e**). Compound **8b** (480 mg, 1.4 mmol) was subjected to the same reaction condition as described for the synthesis of **9a** to give **9e** (330 mg, 94%) as brown solid.^1^H NMR (400 MHz, CDCl_3_) *δ* 7.73 (d, *J* = 7.0 Hz, 1H), 7.16 (d, *J* = 7.0 Hz, 1H), 4.28 (t, *J* = 7.2 Hz, 2H), 3.30 (t, *J* = 6.6 Hz, 2H), 2.62 (s, 3H), 1.92 – 1.83 (m, 2H), 1.61 (dd, *J* = 13.8, 6.8 Hz, 3H), 1.44 (dd, *J* = 12.4, 8.8 Hz, 4H).

1-(7-Azidoheptyl)-3-(hydroxy)-2-methylpyridin-4(1*H*)-one (**9f**). Compound **8f** (261 mg, 0.736 mmol) was subjected to the same reaction condition as described for the synthesis of **9a** to give **9f** (235 mg, 90%) as brown solid. ^1^H NMR (400 MHz, MeOH-*d*_4_) *δ* 8.17 (d, *J* = 7.0 Hz, 1H), 7.12 (d, *J* = 6.9, 1H), 4.38 (t, *J* = 6.3, 2H), 3.28 (t, *J* = 6.8, 2H), 2.64 (s, *J* = 1.6 Hz, 3H), 1.92 – 1.84 (m, 2H), 1.63 – 1.55 (m, 2H), 1.49 – 1.37 (m, 8H).

3-Hydroxy-2-methyl-1-(2-(4-phenyl-1*H*-1,2,3-triazol-1-yl)propyl)pyridin-4(1*H*)-one (**1b**). Diisopropylethylamine (DIPEA; 39 µL, 0.22 mmol) was added to a stirring solution of compound **9b** (18 mg, 0.088 mmol) and Phenylacetylene (19 µL, 0.18 mmol) in mixture of dimethylsulfoxide (2 mL) and tetrahydrofuran (2 mL). Argon was bubbled through the solution for 15 min before and after the addition of Copper (I) iodide (CuI) (15 mg, 0.75 mmol). The reaction mixture was heated at 45 ˚C overnight. The reaction mixture was cooled to room temperature and was diluted with DCM (15 mL) and was sequentially washed with 4:1 mixture of NH_4_Cl:NH_4_OH soln. (3x15 mL), water (3x15 mL), and brine (10 mL), dried over anhydrous Na_2_SO_4_, filtered and concentrated *in vacuo*. The crude was purified by trituration with DCM-Et_2_O (1: 10) mixture to get the target compound **1b** (8.7 mg, 32%) as off-white solid. HPLC retention time 13.1 min. ^1^H NMR (400 MHz, MeOH-*d*_4_) *δ* 8.38 (s, 1H), 7.81 (d, *J* = 7.4 Hz, 2H), 7.63 (d, *J* = 7.0 Hz, 1H), 7.44 (t, *J* = 7.5 Hz, 2H), 7.35 (t, *J* = 7.3 Hz, 1H), 6.39 (d, *J* = 7.0 Hz, 1H), 4.56 (t, *J* = 6.4 Hz, 2H), 4.19 – 4.07 (m, 2H), 3.33 – 3.28 (m, 3H), 2.49 – 2.41 (m, 2H). ^13^C NMR (176 MHz, MeOD) δ 169.3, 147.8, 146.1, 137.5, 131.1, 130.3, 128.6, 128.0, 125.3, 121.0, 111.4, 50.9, 46.9, 30.41, 29.28, 10.4. HRMS (ESI) m/z Calcd. for C_17_H_19_O_2_N_4_ [M+H]^+^: 311.1503, found 311.1502.

3-Hydroxy-2-methyl-1-(2-(4-phenyl-1*H*-1,2,3-triazol-1-yl)butyl)pyridin-4(1*H*)-one (**1c**). Phenylacetylene (25 µL, 0.22 mmol), compound **9c** (25 mg, 0.11 mmol), DIPEA (36 µL, 0.28 mmol), and CuI (16 mg, 0.084 mmol) was reacted in 1:1 DMSO:THF (2 mL) as described for 1b to obtain **1c** as off-white solid (14 mg, 39%). HPLC retention time 13.3 min. ^1^H NMR (700 MHz, MeOH-*d*_4_) *δ* 8.26 (s, 1H), 7.79 (d, *J* = 7.6 Hz, 2H), 7.69 (dd, *J* = 19.3, 16.0 Hz, 1H), 7.41 (t, *J* = 7.5 Hz, 2H), 7.36 – 7.29 (m, 1H), 6.48 (s, 1H), 4.51 – 4.44 (m, 2H), 4.11 (s, 2H), 2.48 – 2.35 (m, 3H), 2.06 – 1.97 (m, 2H), 1.81 (s, 2H). ^13^C NMR (176 MHz, MeOH-*d*_4_) δ 167.9, 154.1, 147.7, 137.5, 132.7, 130.3, 128.6, 128.0, 125.3, 120.8, 111.5, 49.3, 27.2, 26.6, 10.6. HRMS (ESI) m/z Calcd. for C_18_H_21_O_2_N_4_ [M+H]^+^: 325.1659, found 325.11657.

3-Hydroxy-2-methyl-1-(2-(4-phenyl-1*H*-1,2,3-triazol-1-yl)pentyl)pyridin-4(1*H*)-one (**1d**). Phenylacetylene (17 µL, 0.16 mmol), compound **9d** (25 mg, 0.11 mmol), DIPEA (46 µL, 0.26 mmol), and CuI (15 mg, 0.080 mmol) was reacted in 1:1 DMSO:THF (2 mL) as described for 1b to obtain **1d** as off-white solid (18 mg, 50%). HPLC retention time 13.7 min. ^1^H NMR (400 MHz, CDCl_3_/10% MeOH-*d*_4_) *δ* 7.79 (s, 1H), 7.72 (d, *J* = 7.5 Hz, 2H), 7.44 – 7.27 (m, 5H), 6.45 (d, *J* = 7.5 Hz, 1H), 4.36 (t, *J* = 6.8 Hz, 2H), 3.88 (t, *J* = 6.8 Hz, 2H), 2.34 (s, 3H), 1.99 – 1.85 (m, 2H), 1.74 – 1.70 (m, 2H), 1.36 – 1.31 (m, 2H). ^13^C NMR (176 MHz, MeOH-*d*_4_) δ 162.7, 147.6, 137.5, 130.3, 128.6, 128.2, 127.9, 127.3, 125.3, 120.8, 106.3, 49.7, 48.1, 29.5, 29.2, 22.8, 12.9. HRMS (ESI) m/z Calcd. for C_19_H_23_O_2_N_4_ [M+H]^+^: 339.1816, found 339.1810.

3-Hydroxy-2-methyl-1-(2-(4-phenyl-1*H*-1,2,3-rtiazol-1-yl)hexyl)pyridin-4(1*H*)-one (**1e**). Phenylacetylene (16 µL, 0.15 mmol), compound **9e** (25 mg, 0.10 mmol), DIPEA (44 µL, 0.25 mmol), and CuI (14 mg, 0.075 mmol) was reacted in 1:1 DMSO:THF (2 mL) as described for 1b to obtain **1e** as off-white solid (15 mg, 41%). HPLC retention time 14.2 min. ^1^H NMR (400 MHz, MeOH-*d*_4_) *δ* 8.48 (s, 1H), 8.13 (d, *J* = 6.7 Hz, 1H), 7.81 (d, *J* = 8.3 Hz, 3H), 7.46 (t, *J* = 7.5 Hz, 3H), 7.39 (t, *J* = 7.2 Hz, 1H), 7.08 (d, *J* = 6.7 Hz, 1H), 4.51 (t, *J* = 6.8 Hz, 2H), 4.35 (t, *J* = 7.1 Hz, 2H), 2.61 (s, 2H), 2.04 – 2.00 (m, 2H), 1.88 – 1.83 (m, 2H), 1.49 – 1.39 (m, 4H). ^13^C NMR (176 MHz, MeOH-*d*_4_) δ 158.1, 146.9, 143.7, 141.9, 137.7, 129.2, 128.7, 128.4, 125.4, 121.5, 110.3, 56.4, 50.4, 29.7, 29.3, 25.5, 25.2, 11.3. HRMS (ESI) m/z Calcd. for C_20_H_25_O_2_N_4_ [M+H]^+^: 353.1972, found 353.1968.

3-Hydroxy-2-methyl-1-(2-(4-phenyl-1*H*-1,2,3-triazol-1-yl)heptyl)pyridin-4(1*H*)-one (**1f**). Phenylacetylene (16 µL, 0.14 mmol), compound **9f** (25 mg, 0.095 mmol), DIPEA (41 µL, 0.24 mmol), and CuI (14 mg, 0.071 mmol) was reacted in 1:1 DMSO:THF (2 mL) as described for 1b to obtain **1e** as off-white solid (11 mg, 32%). HPLC retention time 14.8 min. ^1^H NMR (700 MHz, MeOH-*d*_4_) *δ* 8.27 (s, 1H), 7.79 (d, *J* = 7.5 Hz, 2H), 7.42 (t, *J* = 7.4 Hz, 2H), 7.33 (t, *J* = 7.2 Hz, 1H), 6.68 (s, 2H), 4.44 (t, *J* = 6.7 Hz, 2H), 4.14 (s, 2H), 2.48 (s, 2H), 1.98 – 1.91 (m, 2H), 1.77 (s, 2H), 1.47 – 1.32 (m, 6H). ^13^C NMR (176 MHz, MeOH-*d*_4_) δ 165.0, 161.8, 147.5, 144.9, 137.5, 130.4, 128.5, 127.9, 125.2, 120.7, 110.8, 49.9, 49.0, 29.9, 29.6, 27.9, 25.7, 10.8. HRMS (ESI) m/z Calcd. for C_21_H_27_O_2_N_4_ [M+H]^+^: 367.2129, found 367.2125.

3-Hydroxy-2-methyl-1-(4-(4-(*o*-tolyl)-1*H*-1,2,3-triazol-1-yl)butyl)pyridin-4(1*H*)-one (**2a**). 2-ethynyltoluene (43 µL, 0.34 mmol), compound **9c** (50 mg, 0.22 mmol), DIPEA (98 µL, 0.56 mmol), and CuI (32 mg, 0.17 mmol) was reacted in 1:1 DMSO:THF (4 mL) as described for 1b to obtain **2a** as off-white solid (39 mg, 52%). HPLC retention time 13.9 min. ^1^H NMR (400 MHz, CDCl_3_/10% MeOH-*d*_4_) *δ* 7.71 – 7.56 (m, 2H), 7.25 – 7.22 (m, 1H), 7.20 (d, *J* = 2.6 Hz, 3H), 6.32 (d, *J* = 6.8 Hz, 1H), 4.40 (t, *J* = 6.1 Hz, 2H), 3.89 (t, *J* = 7.0 Hz, 2H), 2.36 (s, 3H), 2.31 (s, 3H), 1.98 – 1.94 (m, 2H), 1.75 – 1.70 (m, 2H). ^13^C NMR (101 MHz, CDCl_3_) δ 169.4, 147.4, 146.2, 136.6, 135.5, 130.8, 129.6, 128.8, 128.2, 125.9, 121.8, 111.3, 52.9, 49.2, 29.5, 27.7, 26.9, 21.0, 11.6. HRMS (ESI) m/z Calcd. for C_19_H_23_O_2_N_4_ [M+H]^+^: 339.1816, found 339.1813.

3-Hydroxy-2-methyl-1-(5-(4-(*o*-tolyl)-1*H*-1,2,3-triazol-1-yl) pentyl)pyridin-4(1*H*)-one (**2b**). 2-ethynyltoluene (40. µL, 0.32 mmol), compound **9d** (50 mg, 0.21 mmol), DIPEA (92 µL, 0.53 mmol), and CuI (30. mg, 0.16 mmol) was reacted in 1:1 DMSO:THF (4 mL) as described for **1b** to obtain **2b** as off-white solid (41 mg, 54%). HPLC retention time 14.5 min. ^1^H NMR (700 MHz, DMSO-*d*_6_) *δ* 8.34 (s, 1H), 8.22 (d, *J* = 7.0 Hz, 1H), 7.69 (dd, *J* = 7.1, 1.9 Hz, 1H), 7.31 (d, *J* = 6.9 Hz, 1H), 7.29 – 7.23 (m, 3H), 4.41 (t, *J* = 7.0 Hz, 2H), 4.31 (t, *J* = 7.5 Hz, 2H), 2.52 (s, 3H), 2.42 (s, 3H), 1.96 – 1.90 (m, 2H), 1.78 (dt, *J* = 15.4, 7.7 Hz, 2H), 1.31 (dt, *J* = 15.3, 7.7 Hz, 2H). ^13^C NMR (176 MHz, CDCl_3_/10% MeOH-*d*_4_) δ 158.8, 146.0, 143.4, 142.1, 138.5, 135.3, 131.2, 130.6, 128.7, 128.1, 126.4, 123.6, 111.2, 56.2, 49.5, 29.5, 29.3, 23.0, 21.5, 12.9. HRMS (ESI) m/z Calcd. for C_20_H_25_O_2_N_4_ [M+H]^+^: 353.1972, found 353.1971.

3-Hydroxy-2-methyl-1-(4-(4-(*m*-tolyl)-1*H*-1,2,3-triazol-1-yl)butyl)pyridin-4(1*H*)-one (**2c**). 3-ethynyltoluene (44 µL, 0.34 mmol), compound **9c** (50 mg, 0.22 mmol), DIPEA (98 µL, 0.56 mmol), and CuI (32 mg, 0.17 mmol) was reacted in 1:1 DMSO:THF (4 mL) as described for **1b** to obtain **2c** as off-white solid (48 mg, 63%). HPLC retention time 14.2 min. ^1^H NMR (400 MHz, CDCl_3_/10% MeOH-*d*_4_) *δ* 7.76 (s, 1H), 7.56 (s, 1H), 7.49 (d, *J* = 7.5 Hz, 1H), 7.28 – 7.19 (m, 3H), 7.10 (d, *J* = 7.5 Hz, 1H), 6.32 (d, *J* = 6.7 Hz, 1H), 4.38 (t, *J* = 6.2 Hz, 2H), 3.88 (t, *J* = 7.1 Hz, 2H), 2.33 (s, 3H), 2.31 (s, 2H), 1.99 – 1.93 (m, 2H), 1.74 – 1.69 (m, 2H). ^13^C NMR (101 MHz, CDCl_3_/10% MeOH-*d*_4_) δ 169.2, 148.0, 146.2, 138.5, 136.7, 130.0, 129.0, 128.6, 126.2, 122.7, 119.9, 111.4, 53.0, 49.3, 29.5, 27.5, 26.8, 21.1, 11.6. HRMS (ESI) m/z Calcd. for C_19_H_23_O_2_N_4_ [M+H]^+^: 339.1816, found 339.1812.

3-Hydroxy-2-methyl-1-(5-(4-(*m*-tolyl)-1*H*-1,2,3-triazol-1-yl) pentyl)pyridin-4(1*H*)-one (**2d**). 3-ethynyltoluene (55 µL, 0.42 mmol), compound **9d** (50 mg, 0.21 mmol), DIPEA (92 µL, 0.53 mmol), and CuI (30. mg, 0.16 mmol) was reacted in 1:1 DMSO:THF (4 mL) as described for **1b** to obtain **2d** as off-white solid (32 mg, 43%). HPLC retention time 14.5 min. ^1^H NMR (400 MHz, CDCl_3_) *δ* 7.77 (s, 1H), 7.56 (s, 1H), 7.50 (d, *J* = 7.3 Hz, 1H), 7.34 (s, 1H), 7.23 (d, *J* = 7.5 Hz, 1H), 7.09 (d, *J* = 7.5 Hz, 1H), 6.46 (s, 1H), 4.35 (t, *J* = 6.4 Hz, 2H), 3.85 (t, *J* = 7.5 Hz, 2H), 2.32 (s, 6H), 1.95 – 1.89 (m, 2H), 1.73 – 1.66 (m, 2H), 1.35 – 1.31 (m, 2H). ^13^C NMR (176 MHz, DMSO-*d*_6_) *δ* 167.9, 146.8, 145.6, 138.5, 138.1, 131.3, 130.4, 129.2, 128.9, 126.2, 122.8, 121.6, 110.8, 53.3, 49.7, 29.9, 29.5, 23.2, 21.5, 11.9. HRMS (ESI) m/z Calcd. for C_20_H_25_O_2_N_4_ [M+H]^+^: 353.1972, found 353.1969.

3-Hydroxy-2-methyl-1-(4-(4-(*p*-tolyl)-1*H*-1,2,3-triazol-1-yl)butyl)pyridin-4(1*H*)-one (**2e**). 4-ethynyltoluene (44 µL, 0.34 mmol), compound **9c** (50 mg, 0.22 mmol), DIPEA (98 µL, 0.56 mmol), and CuI (32 mg, 0.17 mmol) was reacted in 1:1 DMSO:THF (4 mL) as described for **1b** to obtain **2e** as off-white solid (46 mg, 60%). HPLC retention time 14.1 min. ^1^H NMR (400 MHz, CDCl_3_) *δ* 7.73 (s, 1H), 7.60 (d, *J* = 8.0 Hz, 2H), 7.25 – 7.19 (m, 1H), 7.17 (d, *J* = 7.8 Hz, 2H), 6.32 (d, *J* = 7.1 Hz, 1H), 4.38 (t, *J* = 6.5 Hz, 2H), 3.88 (t, *J* = 7.4 Hz, 2H), 2.31 (s, 6H), 1.97 – 1.92 (m, 2H), 1.76 – 1.71 (m, 2H). ^13^C NMR (101 MHz, CDCl_3_) *δ* 169.3, 148.0, 146.2, 138.1, 136.7, 129.4, 128.7, 127.4, 125.5, 119.5, 111.3, 52.9, 49.3, 29.5, 27.6, 26.8, 20.9, 11.5. HRMS (ESI) m/z Calcd. for C_19_H_23_O_2_N_4_ [M+H]^+^: 339.1816, found 339.1813.

3-Hydroxy-2-methyl-1-(4-(4-(*p*-tolyl)-1*H*-1,2,3-triazol-1-yl) pentyl)pyridin-4(1*H*)-one (**2f**). 4-ethynyltoluene (55 µL, 0.42 mmol), compound **9d** (50 mg, 0.21 mmol), DIPEA (92 µL, 0.53 mmol), and CuI (30. mg, 0.16 mmol) was reacted in 1:1 DMSO:THF (4 mL) as described for **1b** to obtain **2f** as off-white solid (48 mg, 65%). HPLC retention time 14.5 min. ^1^H NMR (400 MHz, CDCl_3_/10% MeOH-*d*_4_) *δ* 7.82 (s, 1H), 7.68 (d, *J* = 7.8 Hz, 2H), 7.3 (s, 1H), 7.25 (d, *J* = 7.6 Hz, 2H), 6.39 (d, 1H), 4.43 (t, *J* = 6.4 Hz, 2H), 3.91 (t, *J* = 7.4 Hz, 2H), 2.38 (s, 6H), 2.05 – 1.92 (m, 2H), 1.82 – 1.74 (m, 2H), 1.42 – 1.35 (m, 2H). ^13^C NMR (101 MHz, CDCl_3_) δ 169.4, 147.9, 146.1, 138.1, 136.8, 129.4, 127.5, 125.5, 119.3, 111.4., 53.5, 49.7, 30.1, 29.5, 23.2, 21.0, 11.6. HRMS (ESI) m/z Calcd. for C_20_H_25_O_2_N_4_ [M+H]^+^: 353.1972, found 353.1970.

3-Hydroxy-1-(4-(4-(2-methoxyphenyl)-1*H*-1,2,3-triazol-1-yl)butyl)-2-methylpyridin-4(1*H*)-one (**2g**). 2-ethynylanisole (58 µL, 0.45 mmol), compound **9c** (50 mg, 0.22 mmol), DIPEA (98 µL, 0.56 mmol), and CuI (32 mg, 0.17 mmol) was reacted in 1:1 DMSO:THF (4 mL) as described for **1b** to obtain **2g** as off-white solid (67 mg, 84%). HPLC retention time 13.7 min. ^1^H NMR (700 MHz, DMSO-*d*_6_) *δ* 8.34 (s, 1H), 8.11 (dd, *J* = 7.6, 1.6 Hz, 1H), 7.57 (d, *J* = 6.7 Hz, 1H), 7.33 – 7.27 (m, 1H), 7.10 (d, *J* = 8.2 Hz, 1H), 7.03 (t, *J* = 7.4 Hz, 1H), 6.15 (d, *J* = 6.7 Hz, 1H), 4.44 (t, *J* = 6.9 Hz, 2H), 3.97 (t, *J* = 7.2 Hz, 2H), 3.89 (s, 3H), 2.27 (s, 3H), 1.93 – 1.83 (m, 2H), 1.73 – 1.59 (m, 2H). ^13^C NMR (176 MHz, DMSO-*d*_6_) *δ* 168.9, 155.9, 145.9, 142.3, 138.1, 129.3, 127.1, 124.3, 121.1, 119.7, 112.1, 111.0, 55.9, 52.8, 49.2, 27.6, 27.2, 11.8. HRMS (ESI) m/z Calcd. for C_19_H_23_O_3_N_4_ [M+H]^+^: 355.1765, found 355.1762.

3-Hydroxy-1-(5-(4-(2-methoxyphenyl)-1*H*-1,2,3-triazol-1-yl)pentyl)-2-methylpyridin-4(1*H*)-one (**2h**). 2-ethynylanisole (55 µL, 0.42 mmol), compound **9d** (50 mg, 0.21 mmol), DIPEA (92 µL, 0.53 mmol), and CuI (30. mg, 0.16 mmol) was reacted in 1:1 DMSO:THF (4 mL) as described for 1b to obtain **2h** as off-white solid (40. mg, 51%). HPLC retention time 14.1 min. ^1^H NMR (400 MHz, CDCl_3_/10%MeOH-*d*_4_) *δ* 8.16 (d, *J* = 7.7, 1H), 7.29 – 7.24 (m, 3H), 7.21 (d, *J* = 6.8 Hz, 1H), 7.04 – 6.97 (m, 1H), 6.93 (d, *J* = 8.3 Hz, 1H), 6.31 (d, *J* = 7.1 Hz, 1H), 4.36 (t, *J* = 6.8 Hz, 2H), 3.87 (s, 3H), 3.82 (t, *J* = 7.6 Hz, 2H), 2.31 (s, 3H), 1.99 – 1.89 (m, 2H), 1.70 (dt, *J* = 15.5, 7.9 Hz, 2H), 1.39 – 1.26 (m, 2H). ^13^C NMR (101 MHz, CDCl_3_/10%MeOH-*d*_4_) *δ* 169.1, 155.6, 146.1, 143.2, 136.7, 129.5, 129.1, 127.2, 123.1, 120.9, 118.7, 111.5, 110.8, 55.2, 53.7, 49.6, 30.1, 29.6, 29.5, 23.1, 11.7. HRMS (ESI) m/z Calcd. for C_20_H_25_O_3_N_4_ [M+H]^+^: 369.1921, found 369.1918.

3-Hydroxy-1-(4-(4-(3-methoxyphenyl)-1*H*-1,2,3-triazol-1-yl)butyl)-2-methylpyridin-4(1*H*)-one (**2i**). 3-ethynylanisole (57 µL, 0.45 mmol), compound **9c** (50 mg, 0.22 mmol), DIPEA (98 µL, 0.56 mmol), and CuI (32 mg, 0.17 mmol) was reacted in 1:1 DMSO:THF (4 mL) as described for **1b** to obtain **2i** as off-white solid (55 mg, 69%). HPLC retention time 13.6 min. ^1^H NMR (400 MHz, MeOH-*d*_4_) *δ* 8.19 – 8.13 (m, 1H), 7.12 (t, *J* = 5.7 Hz, 1H), 4.45 – 4.34 (m, 2H), 3.34 (dd, *J* = 8.6, 4.8 Hz, 2H), 2.64 (d, *J* = 3.8 Hz, 3H), 1.88 (dt, *J* = 15.7, 7.8 Hz, 2H), 1.66 (dt, *J* = 14.8, 6.9 Hz, 2H), 1.48 (ddd, *J* = 15.6, 9.1, 6.0 Hz, 2H). ^13^C NMR (101 MHz, CDCl_3_) δ 169.2, 160.0, 147.8, 146.3, 136.6, 131.5, 129.8, 129.0, 120.1, 118.0, 114.1, 111.3, 111.1, 55.2, 53.0, 49.3, 29.5, 27.6, 26.8, 11.6. HRMS (ESI) m/z Calcd. for C_19_H_23_O_3_N_4_ [M+H]^+^: 355.1765, found 355.1765.

3-Hydroxy-1-(5-(4-(3-methoxyphenyl)-1*H*-1,2,3-triazol-1-yl)pentyl)-2-methylpyridin-4(1*H*)-one (**2j**). 3-ethynylanisole (54 µL, 0.42 mmol), compound **9d** (50 mg, 0.21 mmol), DIPEA (92 µL, 0.53 mmol), and CuI (30. mg, 0.16 mmol) was reacted in 1:1 DMSO:THF (4 mL) as described for **1b** to obtain **2j** as off-white solid (50. mg, 64%). HPLC retention time 13.9 min. ^1^H NMR (400 MHz, CDCl_3_) *δ* 7.73 (s, 1H), 7.45 (s, 1H), 7.39 – 7.31 (m, 2H), 7.18 (t, *J* = 8.4 Hz, 1H), 6.93 – 6.84 (m, 1H), 6.36 (d, *J* = 7.1 Hz, 1H), 4.42 (t, *J* = 6.6 Hz, 2H), 3.87 (s, 3H), 3.83 (t, *J* = 6.6 Hz, 2H), 2.37 (s, 3H), 2.06 – 1.94 (m, 2H), 1.80 – 1.74 (m, 2H), 1.45 – 1.38 (m, 2H). ^13^C NMR (101 MHz, CDCl_3_) *δ* 168.4, 159.9, 147.6, 131.5, 129.9, 120.2, 117.9, 114.1, 110.8, 55.2, 53.8, 49.8, 29.6, 23.2, 11.8. HRMS (ESI) m/z Calcd. for C_20_H_25_O_3_N_4_ [M+H]^+^: 369.1921, found 369.1918.

3-Hydroxy-1-(4-(4-(4-methoxyphenyl)-1*H*-1,2,3-triazol-1-yl)butyl)-2-methylpyridin-4(1*H*)-one (**2k**). 4-ethynylanisole (58 µL, 0.45 mmol), compound **9c** (50 mg, 0.22 mmol), DIPEA (98 µL, 0.56 mmol), and CuI (32 mg, 0.17 mmol) was reacted in 1:1 DMSO:THF (4 mL) as described for **1b** to obtain **2k** as off-white solid (43 mg, 54%). HPLC retention time 13.6 min. ^1^H NMR (400 MHz, CDCl_3_) *δ* 7.68 (d, *J* = 16.0 Hz, 1H), 7.63 (d, *J* = 8.0 Hz, 2H), 7.25 – 7.16 (m, 1H), 6.89 (d, *J* = 7.9 Hz, 2H), 6.31 (d, *J* = 7.9 Hz, 1H), 4.36 (t, *J* = 6.9 Hz, 2H), 3.88 (t, *J* = 6.3 Hz, 2H), 3.77 (s, 3H), 2.30 (s, 3H), 1.96 – 1.90 (m, 2H), 1.77 – 1.68 (m, 2H). ^13^C NMR (101 MHz, CDCl_3_) *δ* 169.4, 159.6, 147.8, 146.2, 136.9, 129.8, 126.9, 122.7, 119.3, 114.3, 111.9, 77.5, 77.1, 76.8, 55.2, 53.1, 49.3, 29.5, 27.6, 26.8, 11.7. HRMS (ESI) m/z Calcd. for C_19_H_23_O_3_N_4_ [M+H]^+^: 355.1765, found 355.1762.

3-Hydroxy-1-(5-(4-(4-methoxyphenyl)-1*H*-1,2,3-triazol-1-yl)pentyl)-2-methylpyridin-4(1*H*)-one (**2l**). 4-ethynylanisole (55 µL, 0.42 mmol), compound **9d** (50 mg, 0.21 mmol), DIPEA (92 µL, 0.53 mmol), and CuI (30. mg, 0.16 mmol) was reacted in 1:1 DMSO:THF (4 mL) as described for **1b** to obtain **2l** as off-white solid (37 mg, 47%). HPLC retention time 13.7 min. ^1^H NMR (700 MHz, DMSO-*d*_6_) *δ* 8.39 (s, 1H), 7.74 (t, *J* = 17.6 Hz, 2H), 7.50 (d, *J* = 7.2 Hz, 1H), 6.99 (d, *J* = 8.5 Hz, 2H), 6.06 (d, *J* = 7.2 Hz, 1H), 4.36 (t, *J* = 6.9 Hz, 2H), 3.89 (t, *J* = 7.5 Hz, 2H), 3.77 (s, 3H), 2.26 (s, 3H), 1.92 – 1.84 (m, 2H), 1.66 (dt, *J* = 15.1, 7.7 Hz, 2H), 1.33 – 1.24 (m, 2H). ^13^C NMR (176 MHz, DMSO) *δ* 169.3, 159.5, 146.7, 137.9, 128.7, 127.0, 124.1, 120.8, 114.8, 110.7, 55.7, 53.1, 49.7, 30.1, 29.6, 23.2, 11.8. HRMS (ESI) m/z Calcd. for C_20_H_25_O_3_N_4_ [M+H]^+^: 369.1921, found 369.1918.

1-(5-(4-(2-Fluorophenyl)-1*H*-1,2,3-triazol-1-yl)pentyl)-3-hydroxy-2-methylpyridin-4(1*H*)-one (**2m**). 1-ethynyl-2-fluorobenzene (48 µL, 0.42 mmol), compound **9d** (50 mg, 0.21 mmol), DIPEA (92 µL, 0.53 mmol), and CuI (30. mg, 0.16 mmol) was reacted in 1:1 DMSO:THF (4 mL) as described for **1b** to obtain **2m** as off-white solid (39 mg, 51%). HPLC retention time 14.2 min. ^1^H NMR (400 MHz, MeOH-*d*_4_) *δ* 8.19 – 8.13 (m, 1H), 7.12 (t, *J* = 5.7 Hz, 1H), 4.45 – 4.34 (m, 2H), 3.34 (dd, *J* = 8.6, 4.8 Hz, 2H), 2.64 (d, *J* = 3.8 Hz, 3H), 1.88 (dt, *J* = 15.7, 7.8 Hz, 2H), 1.66 (dt, *J* = 14.8, 6.9 Hz, 2H), 1.48 (ddd, *J* = 15.6, 9.1, 6.0 Hz, 2H). ^13^C NMR (101 MHz, CDCl_3_) *δ* 169.2, 160.4, 157.9, 146.1, 141.3, 136.8, 129.5, 127.5, 124.6, 122.9, 118.0, 115.8, 111.5, 53.7, 49.8, 30.1, 29.6, 23.2, 11.7. HRMS (ESI) m/z Calcd. for C_19_H_22_O_2_N_4_F [M+H]^+^: 357.1721, found 357.1718.

1-(5-(4-(3-Fluorophenyl)-1*H*-1,2,3-triazol-1-yl)pentyl)-3-hydroxy-2-methylpyridin-4(1*H*)-one (**2n**). 1-ethynyl-2-fluorobenzene (38 µL, 0.32 mmol), compound **9d** (50 mg, 0.21 mmol), DIPEA (92 µL, 0.53 mmol), and CuI (30. mg, 0.16 mmol) was reacted in 1:1 DMSO:THF (4 mL) as described for **1b** to obtain **2n** as off-white solid (48 mg, 64%). HPLC retention time 14.2 min. ^1^H NMR (700 MHz, DMSO-*d*_6_) *δ* 8.61 (d, *J* = 15.2 Hz, 1H), 7.66 (t, *J* = 9.4 Hz, 1H), 7.64 – 7.54 (m, 2H), 7.48 (dt, *J* = 14.2, 7.1 Hz, 1H), 7.13 (td, *J* = 8.5, 2.4 Hz, 1H), 6.19 (d, *J* = 6.9 Hz, 1H), 4.39 (t, *J* = 7.0 Hz, 2H), 3.93 (t, *J* = 7.6 Hz, 2H), 2.29 (s, 3H), 1.95 – 1.84 (m, 2H), 1.72 – 1.60 (m, 2H), 1.34 – 1.25 (m, 2H). ^13^C NMR (176 MHz, DMSO-*d*_6_) *δ* 168.0, 163.8, 162.4, 145.7, 138.1, 133.8, 133.7, 131.5, 131.4, 130.2, 122.4, 121.6, 115.0, 114.8, 112.2, 112.1, 110.9, 53.3, 49.9, 30.0, 29.5, 23.2, 11.9. HRMS (ESI) m/z Calcd. for C_19_H_22_O_2_N_4_F [M+H]^+^: 357.1721, found 357.1718.

1-(5-(4-(4-Fluorophenyl)-1*H*-1,2,3-triazol-1-yl)pentyl)-3-hydroxy-2-methylpyridin-4(1*H*)-one (**2o**). 1-ethynyl-2-fluorobenzene (36 µL, 0.32 mmol), compound **9d** (50 mg, 0.21 mmol), DIPEA (92 µL, 0.53 mmol), and CuI (30. mg, 0.16 mmol) was reacted in 1:1 DMSO-THF (4 mL) as described for **1b** to obtain **2o** as off-white solid (60. mg, 80%). HPLC retention time 14.1 min.^1^H NMR (400 MHz, CDCl_3_) *δ* 7.74 (s, 1H), 7.73 (d, *J* = 9.4 Hz, 1H), 7.71 – 7.67 (m, 2H), 7.72 – 7.64 (m, 2H), 7.22 (d, *J* = 7.2 Hz, 1H), 7.04 (t, *J* = 8.7 Hz, 2H), 6.31 (d, *J* = 7.2 Hz, 1H), 4.34 (t, *J* = 6.9 Hz, 2H), 3.85 – 3.78 (m, 2H), 2.30 (s, 3H), 1.97 – 1.87 (m, 2H), 1.70 (dt, *J* = 15.5, 7.8 Hz, 2H), 1.31 (dq, *J* = 15.7, 7.9 Hz, 2H). ^13^C NMR (101 MHz, CDCl_3_) *δ* 169.1, 163.8, 161.3, 146.8, 146.0, 136.8, 129.2, 127.4, 127.3, 126.7, 126.6, 119.7, 115.7, 115.5, 111.3, 53.5, 49.7, 30.0, 29.5, 23.1, 11.5. HRMS (ESI) m/z Calcd. for C_19_H_22_O_2_N_4_F [M+H]^+^: 357.1721, found 357.1718.

3-Hydroxy-2-methyl -1-(5-(4-(2-(trifluoromethyl)phenyl)-1*H*-1,2,3-triazol-1-yl)pentyl)pyridin-4(1*H*)-one (**2p**). 2-ethynyl-α,α,α-trifluorotoluene (44 µL, 0.32 mmol), compound **9d** (50 mg, 0.21 mmol), DIPEA (92 µL, 0.53 mmol), and CuI (30. mg, 0.16 mmol) was reacted in 1:1 DMSO:THF (4 mL) as described for **1b** to obtain **2p** as off-white solid (70. mg, 82%). HPLC retention time 14.8 min. ^1^H NMR (700 MHz, DMSO-*d*_6_) *δ* 8.27 (s, 1H), 7.89 (t, *J* = 12.8 Hz, 1H), 7.83 (d, *J* = 7.8 Hz, 1H), 7.78 – 7.70 (m, 2H), 7.61 (ddd, *J* = 9.1, 6.4, 3.8 Hz, 1H), 6.73 (d, *J* = 6.5 Hz, 1H), 4.44 (t, *J* = 7.0 Hz, 2H), 4.16 – 4.08 (m, 2H), 2.41 (s, 3H), 1.95 – 1.85 (m, 2H), 1.73 (dt, *J* = 15.3, 7.7 Hz, 2H), 1.29 (dt, *J* = 15.3, 7.7 Hz, 2H). ^13^C NMR (176 MHz, DMSO-*d*_6_) *δ* 163.4, 144.5, 143.7, 143.7, 138.3, 136.2, 133.0, 132.2, 130.2, 130.2, 129.2, 129.2, 127.2, 127.0, 126.8, 126.7, 126.7, 126.7, 126.7, 125.3, 124.4, 124.4, 124.3, 124.3, 123.7, 122.2, 111.1, 54.8, 49.7, 29.6, 23.0, 12.4. HRMS (ESI) m/z Calcd. for C_20_H_22_O_2_N_4_F_3_ [M+H]^+^: 407.1689, found 407.1684.

1-(4-(4-(4-(Dimethylamino)phenyl)-1*H*-1,2,3-triazol-1-yl)butyl)-3-hydroxy-2-methylpyridin-4(1*H*)-one (**2q**). 4-ethynyl-N,N-dimethylaniline (49 mg, 0.34 mmol), compound **9c** (50 mg, 0.22 mmol), DIPEA (98 µL, 0.56 mmol), and CuI (32 mg, 0.17 mmol) was reacted in 1:1 DMSO:THF (4 mL) as described for **1b** to obtain **2q** as off-white solid (36 mg, 43%). HPLC retention time 11.4 min. ^1^H NMR (400 MHz, CDCl_3_/10% MeOH-*d*_4_) *δ* 7.71 (s, 1H), 7.66 (d, *J* = 8.4 Hz, 2H), 7.31 (d, *J* = 6.7 Hz, 1H), 6.79 (d, *J* = 8.3 Hz, 2H), 6.40 (d, *J* = 6.8 Hz, 1H), 4.35 (t, *J* = 6.9 Hz, 2H), 3.86 (t, *J* = 7.4 Hz, 2H), 3.00 (s, 6H), 2.41 (s, 3H), 2.03 – 1.99 (m, 2H), 1.81 – 1.76 (m, 2H), 1.42 – 1.38 (m, 2H). ^13^C NMR (176 MHz, DMSO-*d_6_*) *δ* 163.5, 150.5, 147.5, 144.6, 138.3, 136.3, 126.6, 120.0, 119.4, 112.9, 111.2, 54.4, 49.3, 40.5, 27.4, 26.9, 12.4. HRMS (ESI) m/z Calcd. for C_20_H_26_O_2_N_5_ [M+H]^+^: 368.2081, found 368.2079.

1-(5-(4-(4-(Dimethylamino)phenyl)-1*H*-1,2,3-triazol-1-yl)pentyl)-3-hydroxy-2-methylpyridin-4(1*H*)-one (**2r**). 4-ethynyl-N,N-dimethylaniline (34 mg, 0.23 mmol), compound **9d** (50 mg, 0.21 mmol), DIPEA (92 µL, 0.53 mmol), and CuI (30. mg, 0.16 mmol) was reacted in 1:1 DMSO:THF (4 mL) as described for **1b** to obtain **2r** as off-white solid (44 mg, 54%). HPLC retention time 11.4 min. ^1^H NMR (400 MHz, MeOH-*d*_4_) *δ* 8.19 – 8.13 (m, 1H), 7.12 (t, *J* = 5.7 Hz, 1H), 4.45 – 4.34 (m, 2H), 3.34 (dd, *J* = 8.6, 4.8 Hz, 2H), 2.64 (d, *J* = 3.8 Hz, 3H), 1.88 (dt, *J* = 15.7, 7.8 Hz, 2H), 1.66 (dt, *J* = 14.8, 6.9 Hz, 2H), 1.48 (ddd, *J* = 15.6, 9.1, 6.0 Hz, 2H). ^13^C NMR (176 MHz, DMSO-*d*_6_) *δ* 159.0, 145.5, 143.5, 142.0, 138.5, 127.6, 127.4, 126.6, 125.0, 121.6, 121.3, 111.3, 56.2, 49.7, 49.6, 43.7, 29.5, 29.3, 23.1, 12.9. HRMS (ESI) m/z Calcd. for C_21_H_28_O_2_N_5_ [M+H]^+^: 382.2238, found 382.2234.

3-Hydroxy-2-methyl-1-(5-(4-(pyridin-2-yl)-1*H*-1,2,3-triazol-1-yl)pentyl)pyridin-4(1*H*)-one (**2s**). 2-ethynylpyridine (32 µL, 0.32 mmol), compound **9d** (50 mg, 0.21 mmol), DIPEA (92 µL, 0.53 mmol), and CuI (30. mg, 0.16 mmol) was reacted in 1:1 DMSO:THF (4 mL) as described for **1b** to obtain **2s** as brown solid (47 mg, 65%). HPLC retention time 11.3 min. ^1^H NMR (400 MHz, MeOH-*d*_4_) *δ* 8.19 – 8.13 (m, 1H), 7.12 (t, *J* = 5.7 Hz, 1H), 4.45 – 4.34 (m, 2H), 3.34 (dd, *J* = 8.6, 4.8 Hz, 2H), 2.64 (d, *J* = 3.8 Hz, 3H), 1.88 (dt, *J* = 15.7, 7.8 Hz, 2H), 1.66 (dt, *J* = 14.8, 6.9 Hz, 2H), 1.48 (ddd, *J* = 15.6, 9.1, 6.0 Hz, 2H). ^13^C NMR (101 MHz, CDCl_3_) *δ* 166.9, 149.9, 148.9, 147.9, 145.8, 137.3, 136.8, 131.1, 123.0, 122.4, 120.4, 111.5, 77.2, 54.2, 49.9, 30.0, 29.5, 23.3, 12.0. HRMS (ESI) m/z Calcd. for 18_1_H_22_O_2_N_5_ [M+H]^+^: 340.1768, found 340.1765.

3-Hydroxy-2-methyl-1-(5-(4-(4-(pyridin-4-yl)phenyl)-1*H*-1,2,3-triazol-1-yl)pentyl)pyridin-4(1*H*)-one (**2t**). 4-(4ethynylphenyl)pyridine (32 mg, 0.18 mmol), compound **9d** (35 mg, 0.15 mmol), DIPEA (65 µL, 0.37 mmol), and CuI (21 mg, 0.11 mmol) was reacted in 1:1 DMSO:THF (4 mL) as described for **1b** to obtain **2t** as off-white solid (28 mg, 45%). HPLC retention time 11.0 min. ^1^H NMR (400 MHz, CDCl_3_/10% MeOH-*d*_4_) *δ* 8.52 (dd, *J* = 4.6, 1.5 Hz, 2H), 7.86 (d, *J* = 8.1 Hz, 3H), 7.67 (d, *J* = 8.3 Hz, 2H), 7.52 (dd, *J* = 4.6, 1.6 Hz, 2H), 7.23 (d, *J* = 7.3 Hz, 1H), 6.31 (d, *J* = 7.2 Hz, 1H), 4.38 (t, *J* = 6.8 Hz, 2H), 3.84 (t, *J* = 7.5 Hz, 2H), 2.33 (d, *J* = 6.6 Hz, 3H), 1.94 (dd, *J* = 14.7, 7.4 Hz, 2H), 1.72 (dt, *J* = 15.6, 7.9 Hz, 2H), 1.43 – 1.27 (m, 2H). ^13^C NMR (101 MHz, CDCl_3_) *δ* 169.4, 149.8, 148.0, 147.1, 146.2, 137.5, 136.7, 131.3, 128.8, 127.4, 126.3, 121.4, 120.1, 111.3, 53.6, 49.8, 30.1, 29.6, 23.2, 11.6. HRMS (ESI) m/z Calcd. for C_24_H_26_O_2_N_5_ [M+H]^+^: 416.2081, found 416.2074.

3-Hydroxy-2-methyl-1-(6-(4-(4-(pyridin-4-yl)hexyl)-1*H*-1,2,3-triazol-1-yl)hexyl)pyridin-4(1*H*)-one (**2u**). 4-(4ethynylphenyl)pyridine (22 mg, 0.12 mmol), compound **9e** (30. mg, 0.12 mmol), DIPEA (52 µL, 0.30 mmol), and CuI (17 mg, 0.090 mmol) was reacted in 1:1 DMSO:THF (4 mL) as described for **1b** to obtain **2u** as off-white solid (26 mg, 51%). HPLC retention time 11.5 min. ^1^H NMR (700 MHz, DMSO-*d*_6_) *δ* 8.93 (s, 2H), 8.76 (d, *J* = 7.0 Hz, 1H), 8.37 (d, *J* = 5.3 Hz, 2H), 8.23 (d, *J* = 7.0 Hz, 1H), 8.13 – 8.03 (m, 4H), 7.33 (t, *J* = 7.5 Hz, 1H), 4.41 (t, *J* = 7.0 Hz, 2H), 4.30 (dd, *J* = 19.0, 11.4 Hz, 2H), 2.52 (d, *J* = 6.7 Hz, 3H), 1.89 (dt, *J* = 13.8, 7.0 Hz, 2H), 1.73 (dt, *J* = 15.0, 7.6 Hz, 2H), 1.37 – 1.31 (m, 4H). ^13^C NMR (176 MHz, DMSO-*d*_6_) *δ* 159.0, 154.5, 145.7, 143.4, 142.0, 138.5, 134.3, 134.0, 129.1, 126.5, 123.8, 122.9, 111.3, 56.3, 50.0, 29.8, 25.8, 25.4, 13.0. HRMS (ESI) m/z Calcd. for C_25_H_28_O_2_N_5_ [M+H]^+^: 430.2238, found 430.2230.

1-(6-(4-([1,1^’^-Biphenyl]-4-yl)-1*H*-1,2,3-triazol-1-yl)hexyl)-3-hydroxy-2-methylpyridin-4(1*H*)-one (**2v**). 4-ethynyl-1,1’-biphenyl (26 mg, 0.15 mmol), compound **9e** (35 mg, 0.14 mmol), DIPEA (45 µL, 0.35 mmol), and CuI (20. mg, 0.10 mmol) was reacted in 1:1 DMSO:THF (4 mL) as described for **1b** to obtain **2v** as off-white solid (43 mg, 71%). HPLC retention time 16.8 min. ^1^H NMR (700 MHz, DMSO-*d*_6_) *δ* 8.59 (s, 1H), 7.91 (t, *J* = 8.1 Hz, 3H), 7.71 (dd, *J* = 30.1, 8.0 Hz, 4H), 7.46 (t, *J* = 7.5 Hz, 2H), 7.35 (dd, *J* = 10.7, 4.0 Hz, 1H), 6.78 (t, *J* = 14.1 Hz, 1H), 4.39 (dd, *J* = 15.5, 8.5 Hz, 2H), 4.13 (dd, *J* = 25.4, 18.1 Hz, 2H), 2.38 (d, *J* = 33.9 Hz, 3H), 1.88 (dd, *J* = 10.7, 3.8 Hz, 2H), 1.73 – 1.64 (m, 2H), 1.38 – 1.29 (m, 4H). ^13^C NMR (176 MHz, DMSO-*d*_6_) *δ* 163.3, 146.4, 144.5, 140.1, 139.9, 138.3, 136.4, 130.5, 129.4, 128.0, 127.6, 127.0, 126.1, 121.8, 111.08, 55.0, 50.0, 30.1, 29.8, 25.9, 25.5, 12.5. HRMS (ESI) m/z Calcd. for C_26_H_29_O_2_N_4_ [M+H]^+^: 429.2285, found 429.2277.

3-Hydroxy -1-(6-(4-(6-methoxynaphthalen-2-yl)-1*H*-1,2,3-triazol-1-yl)hexyl)-2-methylpyridin-4(1*H*)-one (**2w**). 2-ethynyl-6-methoxynaphthalene (27 mg, 0.15 mmol), compound **9e** (35 mg, 0.14 mmol), DIPEA (45 µL, 0.35 mmol), and CuI (20. mg, 0.11 mmol) was reacted in 1:1 DMSO:THF (4 mL) as described for **1b** to obtain **2w** as off-white solid (32 mg, 53%). HPLC retention time 15.9 min. ^1^H NMR (700 MHz, DMSO-*d*_6_) *δ* 8.58 (s, 1H), 8.28 (s, 1H), 7.91 (d, *J* = 8.4 Hz, 1H), 7.85 (dd, *J* = 8.5, 5.9 Hz, 2H), 7.55 (dd, *J* = 23.4, 8.2 Hz, 1H), 7.30 (dd, *J* = 19.9, 5.5 Hz, 1H), 7.17 (dd, *J* = 8.9, 2.5 Hz, 1H), 6.12 (dd, *J* = 22.5, 7.2 Hz, 1H), 4.39 (t, *J* = 7.0 Hz, 2H), 3.91 – 3.88 (m, 2H), 3.87 (s, 3H), 2.27 (d, *J* = 7.7 Hz, 3H), 1.95 – 1.82 (m, 2H), 1.68 – 1.55 (m, 2H), 1.36 – 1.25 (m, 4H). ^13^C NMR (176 MHz, DMSO-*d*_6_) *δ* 169.0, 157.9, 147.0, 145.9, 138.1, 134.3, 129.9, 129.1, 127.8, 126.7, 124.6, 123.8, 121.6, 119.5, 110.8, 106.7, 55.7, 53.2, 49.9, 30.5, 29.9, 25.9, 25.6, 11.8. HRMS (ESI) m/z Calcd. for C_25_H_29_O_3_N_4_ [M+H]^+^: 433.2234, found 433.2226.

1,1′-(Butane-1,4-diyl)bis(3-(benzyloxy)-2-methylpyridin-4(1 *H*)-one (**12**). 3-Benzyloxy-2-methyl-4-pyrone **7** (101 mg, 0.47 mmol), 1,4-diaminobutane **19** (14 mg, 0.16 mmol), and sodium hydroxide (3.2 mg, 0.08 mmol) were suspended in 2:1 MeOH-H_2_O (6 mL) mixture and the mixture was heated in a sealed tube at 105 °C for 72 h. The mixture was then cooled to room temperature and the product was extracted with DCM (50 mL) and then the organic layer was washed with water (2 x 10 mL) and brine (10 mL), dried over anhydrous Na_2_SO_4_, filtered and concentrated *in* *vacuo*. The crude was purified by preparative chromatography (eluent 15% MeOH in CHCl_3_ containing 1% Et_3_N) to give the title compound **12** (45 mg, 19%) as white solid. ^1^H NMR (400 MHz, MeOH-*d*_4_) *δ* 7.55 (dd, *J* = 7.4, 2.1 Hz, 1H), 7.26 (dt, *J* = 5.3, 2.4 Hz, 2H), 7.19 (dt, *J* = 5.6, 2.2 Hz, 3H), 6.35 (dd, *J* = 7.4, 2.1 Hz, 1H), 4.97 (d, *J* = 2.1 Hz, 2H), 3.84 (m, 2H), 2.03 (s, 3H), 1.52 (m, 2H). HRMS (ESI) m/z Calcd. for C_30_H_33_O_4_N_2_ [M+H]^+^: 485.2435, found 485.2423.

1,1′-(Pentane-1,5-diyl)bis(3-(benzyloxy)-2-methylpyridin-4(1 *H*)-one (**13**). 3-Benzyloxy-2-methyl-4-pyrone **7** (188 mg, 0.87 mmol), 1,5-diaminopentane **20** (0.04 mL, 0.29 mmol), and sodium hydroxide (5.8 mg, 0.15 mmol) in methanol-H_2_O (2:1, 3 mL) mixture were subjected to the same reaction condition as described for the synthesis of **12**, afforded **13** (28 mg, 19%) as white solid after purification by preparative chromatography (eluent 10% MeOH in CHCl_3_ containing 1% NH_4_OH soln.). ^1^H NMR (400 MHz, CDCl_3_) *δ* 7.36 (dq, *J* = 4.5, 2.5 Hz, 2H), 7.33 – 7.25 (m, 3H), 7.12 (d, *J* = 7.5 Hz, 1H), 6.37 (d, *J* = 7.5 Hz, 1H), 5.19 (s, 2H), 3.69 (t, *J* = 7.4 Hz, 2H), 2.05 (s, 3H), 1.68 – 1.53 (m, 2H), 1.21 (d, *J* = 7.8 Hz, 2H). HRMS (ESI) m/z Calcd. for C_31_H_35_O_4_N_2_ [M+H]^+^: 499.2591, found 499.2581.

1,1′-(Hexane-1,6-diyl)bis(3-(benzyloxy)-2-methylpyridin-4(1 *H*)-one (**14**). 3-Benzyloxy-2-methyl-4-pyrone **7** (115 mg, 0.53 mmol), 1,6-diaminohexane **21** (20 mg, 0.18 mmol), and sodium hydroxide (3.6 mg, 0.09 mmol) ) in methanol-H_2_O (2:1, 3 mL) mixture were subjected to the same reaction condition as described for the synthesis of **12**, afforded **14** (68 mg, 74%) as white solid after purification by preparative chromatography (eluent 10% MeOH in DCM containing 1% NH_4_OH soln.). ^1^H NMR (400 MHz, CDCl_3_) *δ* 7.38 (dd, *J* = 7.7, 1.9 Hz, 2H), 7.32 – 7.25 (m, 3H), 7.12 (d, *J* = 7.6 Hz, 1H), 6.39 (d, *J* = 7.5 Hz, 1H), 5.21 (s, 2H), 3.77 – 3.65 (m, 2H), 2.06 (s, 3H), 1.61 (m, 2H), 1.35 – 1.17 (m, 2H). HRMS (ESI) m/z Calcd. for C_32_H_37_O_4_N_2_ [M+H]^+^: 513.2748, found 513.2736.

1,1′-(Heptane-1,7-diyl)bis(3-(benzyloxy)-2-methylpyridin-4(1 *H*)-one (**15**). 3-Benzyloxy-2-methyl-4-pyrone **7** (136 mg, 0.63 mmol), 1,7-diaminoheptane **22** (27 mg, 0.21 mmol), and sodium hydroxide (4.15 mg, 0.10 mmol) in methanol-H_2_O (2:1, 3 mL) mixture were subjected to the same reaction condition as described for the synthesis of **12**, afforded **15** (47 mg, 43%) as white solid after purification by preparative chromatography (eluent 10% MeOH in DCM containing 0.5% NH_4_OH soln.). ^1^H NMR (400 MHz, CDCl_3_) *δ* 7.42 – 7.36 (m, 2H), 7.33 – 7.25 (m, 3H), 7.13 (d, *J* = 7.5 Hz, 1H), 6.39 (d, *J* = 7.5 Hz, 1H), 5.21 (s, 2H), 3.70 (t, *J* = 7.4 Hz, 2H), 2.09 – 2.03 (m, 2H), 1.24 (m, 3H). HRMS (ESI) m/z Calcd. for C_33_H_39_O_4_N_2_ [M+H]^+^: 527.2904, found 527.2892.

1,1′-((1*r*, 4*r*)-Cyclohexane-1,4-diyl)bis(3-(benzyloxy)-2-methylpyridin-4(1 *H*)-one) (**16**). 3-Benzyloxy-2-methyl-4-pyrone **7** (139 mg, 0.64 mmol), *trans*-1,4-diaminocyclohexane **23** (38 mg, 0.32 mmol), and sodium hydroxide (6.40 mg, 0.16 mmol) in methanol-H_2_O (2:1, 3 mL) mixture were subjected to the same reaction condition as described for the synthesis of **12**, afforded **16** (24 mg, 14%) as white solid after purification by preparative chromatography (eluent 10% MeOH in DCM containing 0.5% NH_4_OH soln.). ^1^H NMR (400 MHz, CDCl_3_) *δ* 7.37 (dq, *J* = 6.7, 2.5 Hz, 2H), 7.35 – 7.26 (m, 4H), 6.45 (dd, *J* = 7.7, 2.1 Hz, 1H), 5.18 (d, *J* = 2.2 Hz, 2H), 3.97 (s, 1H), 2.14 (s, 3H), 2.06 (d, *J* = 8.2 Hz, 2H), 1.91 – 1.76 (m, 2H). HRMS (ESI) m/z Calcd. for C_32_H_35_O_4_N_2_ [M+H]^+^: 511.2591, found 511.2579.

1,1′-(1,4-Phenylenebis(methylene))bis(3-(benzyloxy)-2-methylpyridin-4(1 *H*)-one) (**17**). 3-Benzyloxy-2-methyl-4-pyrone **7** (115 mg, 0.53 mmol), *p-*xylenediamine **24** (24 mg, 0.18 mmol), and sodium hydroxide (3.6 mg, 0.09 mmol) in methanol-H_2_O (2:1, 3 mL) mixture were subjected to the same reaction condition as described for the synthesis of **12**, afforded **17** (29 mg, 30%) as white solid after purification by preparative chromatography (eluent 10% MeOH in DCM containing 0.5% NH_4_OH soln.). ^1^H NMR (400 MHz, CDCl_3_) *δ* 7.37 (dd, *J* = 7.5, 2.0 Hz, 2H), 7.30 – 7.17 (m, 4H), 6.83 (d, *J* = 1.1 Hz, 2H), 6.51 – 6.42 (m, 1H), 5.25 (s, 2H), 4.93 (s, 2H), 1.94 (s, 3H). HRMS (ESI) m/z Calcd. for C_34_H_33_O_4_N_2_ [M+H]^+^: 533.2435, found 533.2422.

1,1′-(Methylenebis(cyclohexane-4,1-diyl))bis(3-(benzyloxy)-2-methylpyridin-4(1 *H*)-one) (**18**). 3-Benzyloxy-2-methyl-4-pyrone **7** (106 mg, 0.49 mmol), 4,4′-methylenebis(cyclohexylamine) **25** (109 mg, 0.49 mmol), and sodium hydroxide (20 mg, 0.49 mmol) in methanol-H_2_O (2:1, 3 mL) mixture were subjected to the same reaction condition as described for the synthesis of **12**, afforded **18** (37 mg, 12%) as white solid after purification by preparative chromatography (eluent 10% MeOH in DCM containing 0.5% NH_4_OH soln.). ^1^H NMR (400 MHz, CDCl_3_) *δ* 7.43 – 7.36 (m, 2H), 7.36 – 7.26 (m, 4H), 6.44 (dd, *J* = 7.8, 5.4 Hz, 1H), 5.20 (s, 2H), 3.81 (s, 1H), 2.11 (s, 3H), 1.89 (d, *J* = 11.9 Hz, 3H), 1.81 – 1.50 (m, 7H). HRMS (ESI) m/z Calcd. for C_39_H_47_O_4_N_2_ [M+H]^+^: 607.3530, found 607.3519.

1,1′-(Butane-1,4-diyl)bis(3-hydroxy-2-methylpyridin-4(1 *H*)-one (**3a**). Compound **12** (45 mg, 0.09 mmol) was suspended in 3 mL of conc. HCl solution and the suspension was stirred at room temperature for 3 h. The solution became homogenous eventually and TLC indicated the complete disappearance of the starting material. The excess HCl solution was removed under vacuum and the residue was lyophilized to get title compound **3a** (3.5 mg, 12%) as off-white solid. HPLC retention time 7.2 min, purity 94%. RT ^1^H NMR (500 MHz, MeOH-*d*_4_) δ 8.10 (d, *J* = 7.0 Hz, 1H), 7.02 (d, *J* = 6.9 Hz, 1H), 4.41 – 4.29 (m, 2H), 2.56 (s, 3H), 1.87 (p, *J* = 3.4 Hz, 2H). ^13^C NMR (126 MHz, MeOH-*d*_4_) *δ* 159.6, 143.2, 139.1, 111.8, 57.0, 28.0, 12.8. HRMS (ESI) m/z Calcd. for C_16_H_21_O_4_N_2_ [M+H]^+^: 305.1496, found 304.1494.

1,1′-(Pentane-1,5-diyl)bis(3-hydroxy-2-methylpyridin-4(1 *H*)-one (**3b**). Following the same reaction protocol as described for the synthesis of **3a**, conc. HCl (4 mL) treatment of compound **13** (28 mg, 0.06 mmol) gave the title compound **3b** (12 mg, 66%) as off-white solid. HPLC retention time 9.8 min, purity 94% . ^1^H NMR (700 MHz, MeOH-*d*_4_) *δ* 8.10 (d, *J* = 6.9 Hz, 1H), 7.02 (dd, *J* = 6.9, 1.9 Hz, 1H), 4.29 (t, *J* = 7.8 Hz, 2H), 2.54 (s, 3H), 1.88 – 1.72 (m, 2H), 1.51 – 1.26 (m, 1H). ^13^C NMR (176 MHz, MeOH-*d*_4_) *δ* 159.5, 145.1, 143.2, 139.2, 111.7, 57.5, 30.8, 23.9, 12.8. HRMS (ESI) m/z Calcd. for C_17_H_23_O_4_N_2_ [M+H]^+^: 319.1652, found 319.1650.

1,1′-(Hexane-1,6-diyl)bis(3-hydroxy-2-methylpyridin-4(1 *H*)-one (**3c**). Following the same reaction protocol as described for the synthesis of **3a**, conc. HCl (3 mL) treatment of compound **14** (68 mg, 0.13 mmol) gave the title compound **3c** (14 mg, 31%) as off-white solid. HPLC retention time 10.4 min, purity 93%. RT ^1^H NMR (700 MHz, MeOH-*d*_4_) *δ* 8.00 (s, 1H), 6.94 (d, *J* = 6.9 Hz, 1H), 4.22 (dd, *J* = 8.7, 6.8 Hz, 2H), 2.50 (s, 3H), 1.74 (q, *J* = 7.3 Hz, 2H), 1.40 – 1.31 (m, 2H). ^13^C NMR (176 MHz, MeOH-*d*_4_) *δ* 162.5, 139.0, 130.3, 129.6, 111.7, 57.9, 31.1, 26.8, 12.7. HRMS (ESI) m/z Calcd. for C_18_H_25_O_4_N_2_ [M+H]^+^: 333.1809, found 333.1807.

1,1′-(Heptane-1,7-diyl)bis(3-hydroxy-2-methylpyridin-4(1 *H*)-one (**3d**). Following the same reaction protocol as described for the synthesis of **3a**, conc. HCl (2 mL) treatment of compound **15** (47 mg, 0.09 mmol) gave the title compound **3d** (26 mg, 83%) as off-white solid. HPLC retention time 11.1 min, purity 89%. ^1^H NMR (500 MHz, MeOH-*d*_4_) *δ* 7.97 (d, *J* = 6.9 Hz, 1H), 7.04 (d, *J* = 6.9 Hz, 1H), 4.23 (t, *J* = 7.7 Hz, 2H), 2.50 (s, 3H), 1.72 (dq, *J* = 14.6, 6.8 Hz, 2H), 1.36 – 1.18 (m, 3H). ^13^C NMR (126 MHz, MeOH-*d*_4_) *δ* 159.3, 144.5, 142.9, 139.1, 111.7, 57.8, 30.9, 29.27, 26.7, 12.9. HRMS (ESI) m/z Calcd. for C_19_H_27_O_4_N_2_ [M+H]^+^: 347.1965, found 347.1964.

1,1′-((1*r*, 4*r*)- Cyclohexane-1,4-diyl)bis(3-hydroxy-2-methylpyridin-4(1 *H*)-one) (**3e**). Following the same reaction protocol as described for the synthesis of **3a**, conc. HCl (2 mL) treatment of compound **15** (24 mg, 0.05 mmol) gave the title compound **3e** (8.32 mg, 52%) as off-white solid. HPLC retention time 14.7 min, purity 91%. ^1^H NMR (500 MHz, MeOH-*d*_4_) *δ* 8.26 (d, *J* = 7.2 Hz, 1H), 7.14 (d, *J* = 7.1 Hz, 1H), 2.68 (s, 3H), 2.32 – 2.12 (m, 4H). ^13^C NMR (126 MHz, MeOH-*d*_4_) *δ* 160.2, 144.9, 142.8, 135.40, 112.1, 32.0. HRMS (ESI) m/z Calcd. for C_18_H_23_O_4_N_2_ [M+H]^+^: 331.1652, found 331.1642.

1,1′-(1,4-Phenylenebis(methylene))bis(3-hydroxy-2-methylpyridin-4(1 *H*)-one) (**3f**). Following the same reaction protocol as described for the synthesis of **3a**, conc. HCl (2 mL) treatment (*overnight instead of 3 h*) of compound **15** (29 mg, 0.05 mmol) gave the title compound **3f** (13 mg, 68%) as off-white solid. HPLC retention time 15.1 min. purity 93%. ^1^H NMR (500 MHz, MeOH-*d*_4_) *δ* 8.16 (d, *J* = 7.0 Hz, 1H), 7.30 – 7.11 (m, 2H), 7.07 (d, *J* = 6.9 Hz, 1H), 5.55 (s, 2H), 2.38 (s, 3H). ^13^C NMR (126 MHz, MeOH-*d*_4_) *δ* 160.5, 145.6, 143.4, 140.0, 136.1, 129.0, 111.8, 60.4, 13.1. HRMS (ESI) m/z Calcd. for C_20_H_21_O_4_N_2_ [M+H]^+^: 353.1496, found 353.1493.

1,1′-(Methylenebis(cyclohexane-4,1-diyl))bis(3-hydroxy-2-methylpyridin-4(1 *H*)-one) (**3g**). Following the same reaction protocol as described for the synthesis of **3a**, conc. HCl (4 mL) treatment (*overnight instead of 3 h*) of compound **15** (37 mg, 0.06 mmol) gave the title compound **3g** (15 mg, 57%) as off-white solid. HPLC retention time 13.3 min, purity 99%. ^1^H NMR (500 MHz, MeOH-*d*_4_) *δ* 8.40 – 8.15 (m, 1H), 7.07 (d, *J* = 5.8 Hz, 1H), 4.46 (d, *J* = 12.7 Hz, 1H), 2.59 (d, *J* = 2.3 Hz, 3H), 2.10 – 1.62 (m, 7H), 1.60 – 1.39 (m, 1H), 1.17 (d, *J* = 10.3 Hz, 2H). ^13^C NMR (126 MHz, MeOH-*d*_4_) *δ* 159.2, 144.7, 111.9, 65.3, 34.5, 33.7, 33.1, 30.2, 28.7, 12.6. HRMS (ESI) m/z Calcd. for C_25_H_35_O_4_N_2_ [M+H]^+^: 427.2591, found 427.2587.

**Supplementary Experimental**

**KDM Cell-free Assay.** This experiment was done through a contractual agreement with BPS Bioscience, San Diego, CA. All of the enzymatic reactions were conducted in duplicate at room temperature for 60 min in a 10 µL mixture containing assay buffer, histone H3 peptide substrate, demethylase enzyme, and the test compound. These 10 µL reactions were carried out in wells of 384-well Optiplate (PerkinElmer). The serial dilution of the compounds was first performed in 100% DMSO with the highest concentration at 2.5mM. Each intermediate compound dilution (in 100% DMSO) was diluted 30x fold into assay buffer for 3.3x conc (DMSO). Enzyme only and blank only wells have a final DMSO concentration of 1%. From this intermediate step, 3 µL of compound was added to 4 µL of demethylase enzyme and the dilution was incubated for 30 min at room temperature. After this incubation, 3 µL of peptide substrate was added. The final DMSO concentration in each experiment was 1%. After enzymatic reactions, 5 µL of anti-Mouse Acceptor beads (PerkinElmer, diluted 1:500 with 1x detection buffer) or 5 µL of anti-Rabbit Acceptor beads (PerkinElmer, diluted 1:500 with 1x detection buffer) and 5 µL of primary antibody (BPS, diluted 1:200 with 1x detection buffer) were added to the reaction mix. After brief shaking, plate was incubated for 30 min. Finally, 10 µL of AlphaScreen Streptavidin-conjugated donor beads (Perkin, diluted 1:125 with 1x detection buffer) were added. In 30 min, the samples were measured in AlphaScreen microplate reader (EnSpire Alpha 2390 Multilabel Reader, PerkinElmer).

Data Analysis

The AlphaScreen intensity data were analyzed and compared. In the absence of the compound, the intensity in each data set was defined as 100% activity. In the absence of enzyme, the intensity in each data set was defined as 0% activity. The values of % activity versus a series of compound concentrations were then plotted using non-linear regression analysis of Sigmoidal dose-response curve generated with the equation Y=B+(T-B)/1+10^((LogEC50-X)×Hill Slope)^, where Y=percent activity, B=minimum percent activity, T=maximum percent activity, X= logarithm of compound and Hill Slope=slope factor or Hill coefficient. The IC_50_ value was determined by the concentration causing a half-maximal percent activity.

**Western Blot Analysis for Histone H3 Methylation.** The MCF-7 cells cultured in non-phenol red DMEM with 10% charcoal stripped FBS (Atlanta Biologicals, GA). Prior to treatment with the appropriated agents, the cells (10^6^ cells per well) were plated in a 6-well plate (Techno Plastic Products AG, Trasadingen, Switzerland). After 24 hours incubation at 37 ˚C under a 5% CO_2_ atmosphere, the initial media was aspirated, and the cells were treated with the compound in triplicate with a final concentration of 1% DMSO. Subsequent to treatment, the cells were incubated under the same condition for 72 hours and lysed in the RIPA lysis buffer (10 mM Tris-Cl (pH 8.0), 1 mM EDTA, 1% Triton X-100, 0.1% sodium deoxycholate, 0.1% SDS, 140 mM NaCl, and 1 mM PMSF). Cell lysates were centrifuged at 14,000 rpm at 4^0^C for 10 minutes, and the supernatant was transferred to a new tube, mixed with SDS loading dye (100 mM Tris-Cl (pH 6.8), 4% (w/v) sodium dodecyl sulfate, 0.2% bromophenol blue, 200 mM dithiothreitol), followed by heating at 100^0^C for 5 minutes. Samples were analyzed for the expression levels of H3K4me3 and H3K27me3 by Western Blotting using an anti-H3K4me3 antibody (Millipore, 07-473) and an anti-H3K27me3 antibody (Millipore, 07-449), respectively. Western blotting of cell lysates with an anti-β-ACTIN antibody (Sigma-Aldrich, A5316) was included as a loading control. Cell lysates in SDS loading dye as described above were loaded onto 15% Acrylamide Gels for SDS Polyacrylamide Gel Electrophoresis (SDS-PAGE), followed by transferring the proteins to nitrocellulose membranes (Bio-Rad, Cat# 162-0115) using a BioRad Trans-Blot® Electrophoretic Transfer Cell according to the manufacturer’s manual. The membranes were subsequently incubated for one hour with blocking buffer (Odyssey® Blocking Buffer (PBS), Cat# 927-40000), followed by incubation with the primary antibodies for one hour at room temperature. The membranes were washed and incubated for one hour in room temperature in blocking buffer with secondary antibodies and washed. The images of the Western blots were scanned by LI-COR's Odyssey^®^ (LI-COR) and the signal intensities were quantified using Image Studio^™^ software (LI-COR).

**Cell Viability Assay.** All cell lines were purchased form ATCC (Manassas, VA), and they were routinely cultured in different media recommended by the supplier. MCF-7 cells were cultured in non-phenol red DMEM (Corning, Manassas, VA) fortified with 10% charcoal treated fetal bovine serum (FBS; Atlanta Biologicals, GA) and 0.2 mM glutamine (Corning, Manassas, VA), whereas MDA-MB-231, DU-145, and Vero were cultured in DMEM with 10% FBS. Prior to addition of the ligand, all cell lines were plated onto a 96 well plate (Techno Plastic Products AG, Trasadingen, Switzerland) using non-phenol red DMEM with cell density of 4500 cells/100 µL. After 24 h incubation in 37 ˚C under 5% CO_2_, cells were treated with serially diluted ligands with final DMSO concentration of 1% and incubated for additional 72 h. MTS (CellTiter 96 Aqueous One Solution Cell Proliferation Assays, Promega, Madison, WI) was added to each well 2.5 h prior to recording the absorbance at 490 nm.

**Culture of Mouse Embryonic Stem Cell Lines**

Mouse embryonic stem cells were adapted to be grown feeder free on gelatin coated plates in DMEM supplemented with 4.5 g/L glucose, 15% FBS, L-glutamate, sodium pyruvate, HEPES buffer, NEAA, 2-mercaptoethanol, LIF, and penicillin/streptomycin (ES Media) at 37°C supplemented with 5% CO_2_. ES media was aspirated and replaced daily.

The *CiA:Oct4* recruitment system in mouse embryonic stem cells contains Gal4 and Zinc finger DNA binding arrays and a downstream nuclear eGFP gene in place of a single *Oct4* allele as previously described.^30^ The *CiA:Oct4* N118/N163 cell line containing viral integrations of N118 and N163 plasmids (N118- nLV EF-1α-Gal-FKBPx1-HA-PGK-Blast, N163- nLV EF-1α-HP1α (CS)-Frbx2(Frb+FrbWobb)-V5-PGK-Puro) was used for inhibition of HP1-mediated heterochromatin studies. All plasmids are freely available on addgene.

**HP1-Recruitment Assay Dose Curve.** Day 0, *CiA:Oct4* N118/163 cells were grown in ES media and seeded at a density of 10,000 cells per well in 100 μL media (100,000 cells mL^-1^) on gelatin coated 96 well plates. Day 1, culture media was aspirated and replaced with 100 µL ES media containing +/- 6 nM rapamycin. 10 µM, 5 µM, 2.5 µM, 1 µM, 500 nM, and 250 nM concentrations of compound or DMSO were added using a TTP Labtech Mosquito HTS liquid handler. Days 2, fresh ES media +/- rapamycin and compound were added as in Day 1. Day 3, cells were washed with PBS and trypsinized using 0.25% trypsin-EDTA. Trypsin was quenched with serum. Cells were resuspended by pipetting in preparation for flow cytometry analysis.

**Flow Cytometry and Analysis**. Flow cytometry data was acquired using the Intellicyt iQue Screener and analyzed with FlowJo software. Cell populations were gated based on forward and side scatter height. Single cell populations were gated using forward scatter area by forward scatter height. Autofluorescent cells were excluded and the remaining cells were gated into GFP (-) and GFP (+) populations based on DMSO control samples. Histograms demonstrate representative samples while dose curve scatter plots contain all biological replicates (n=6).

**^1^H and ^13^C Spectra of 1a – f, 2a – w, and 3a – g**


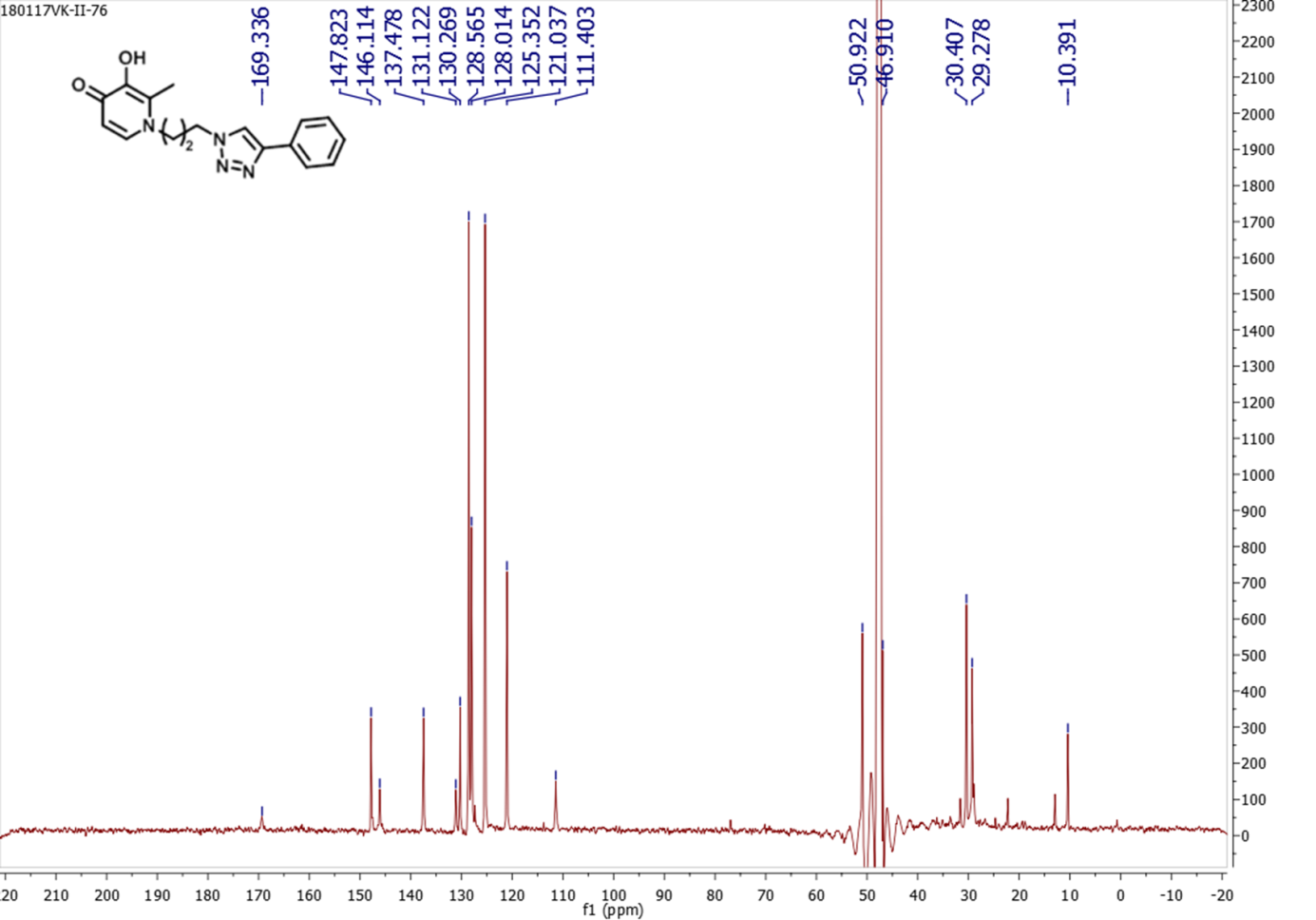


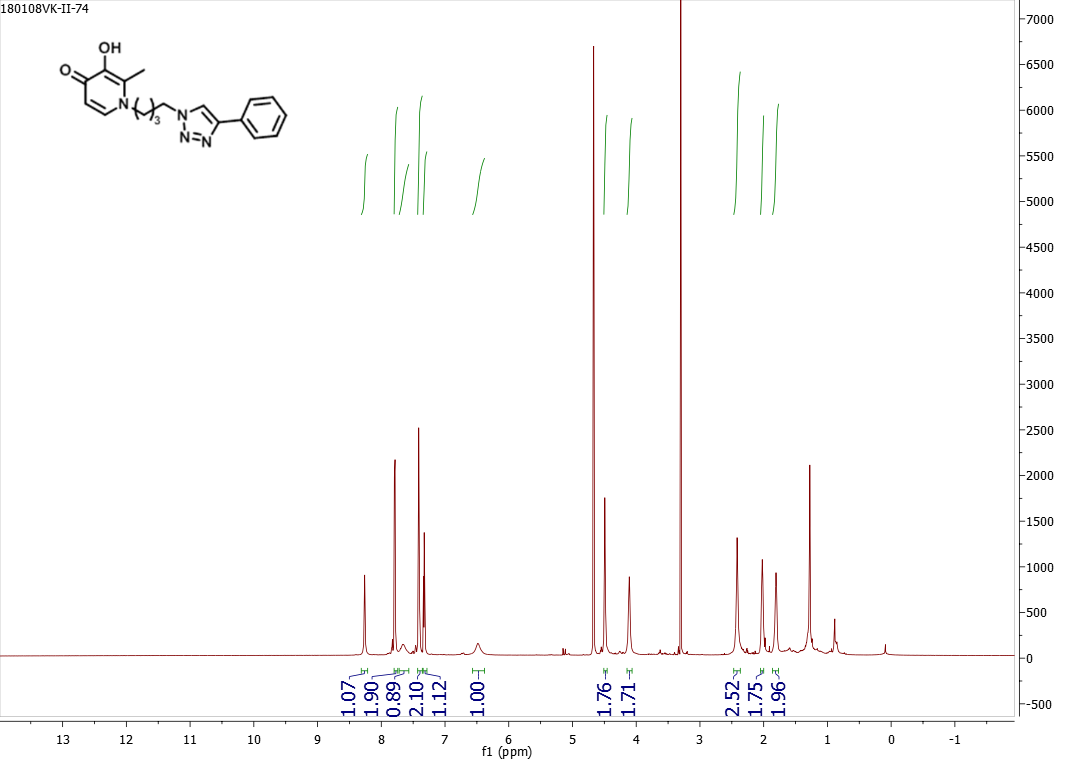


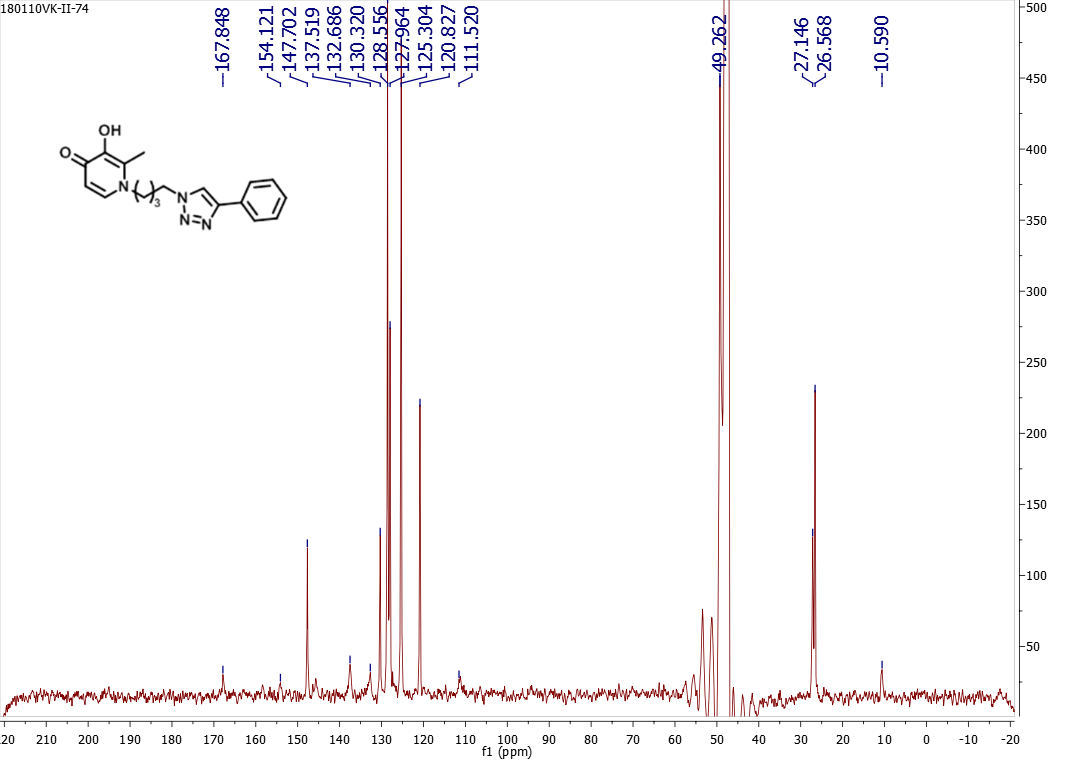


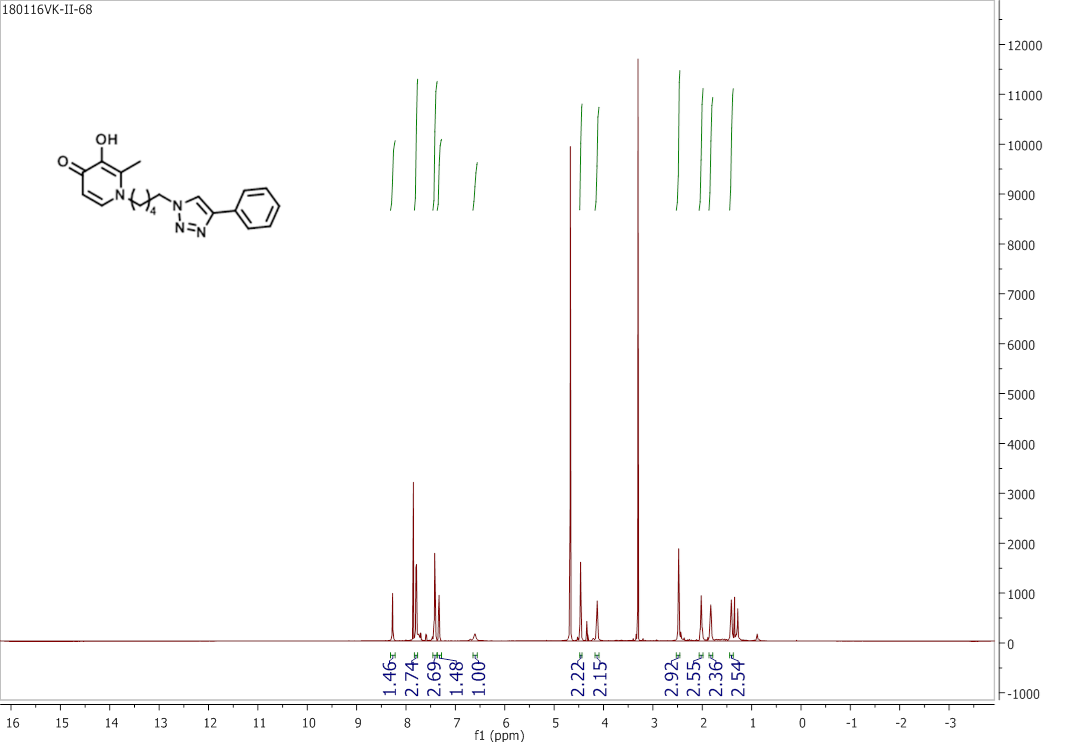


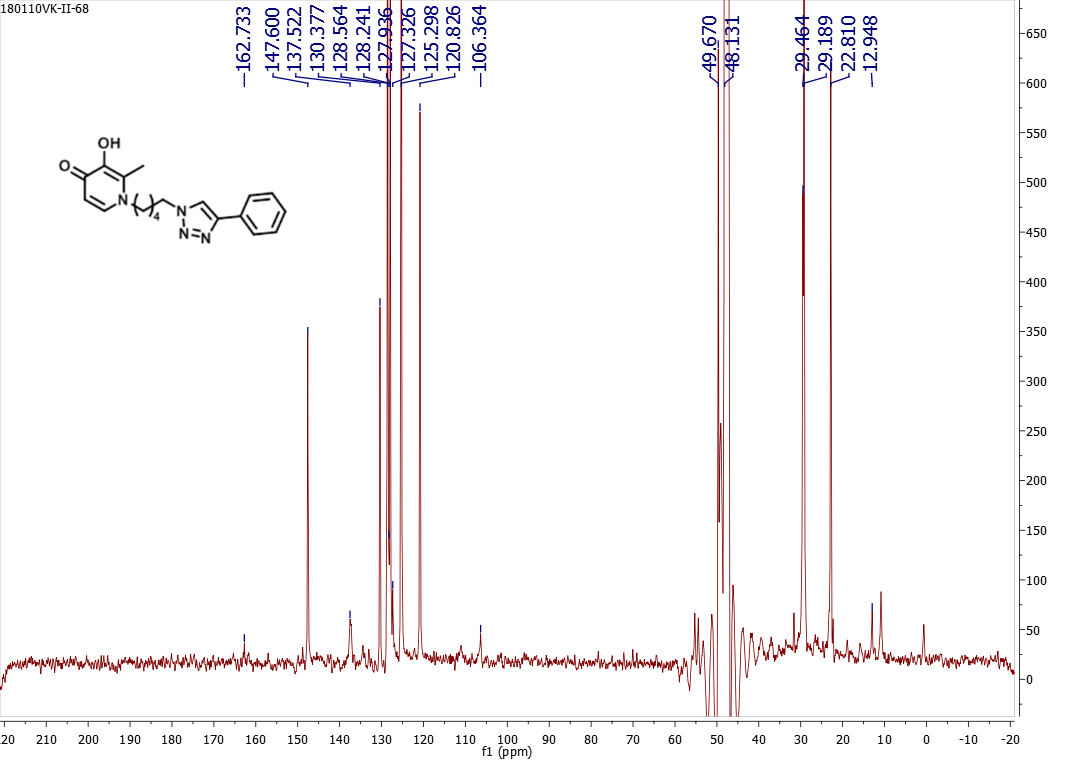


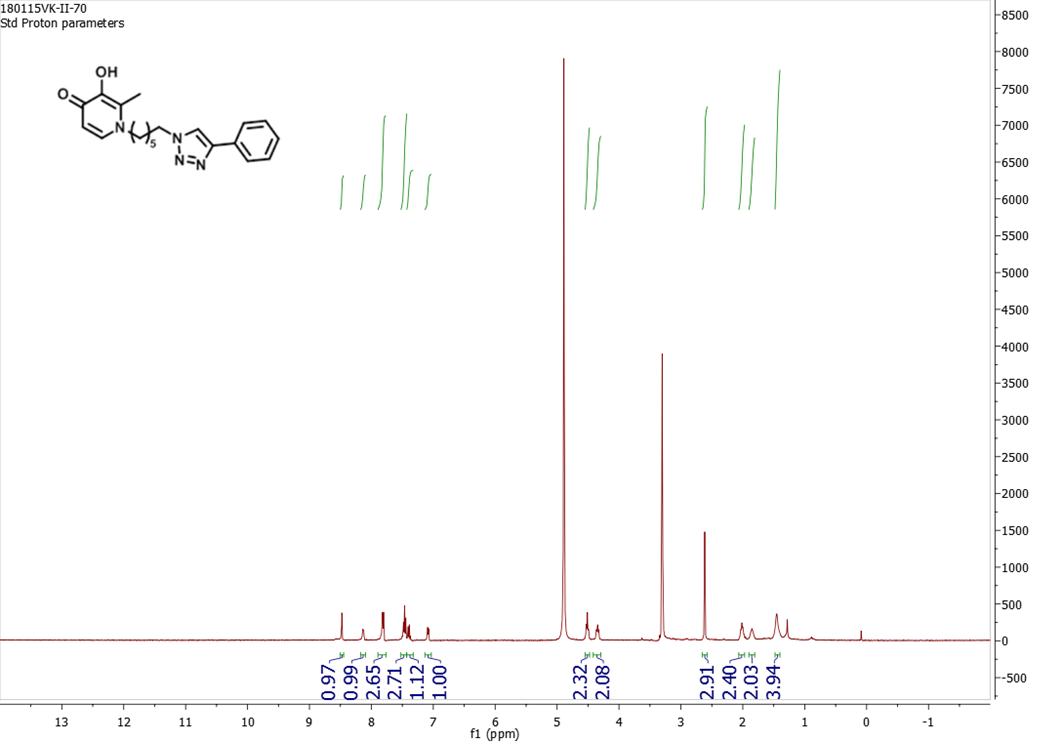


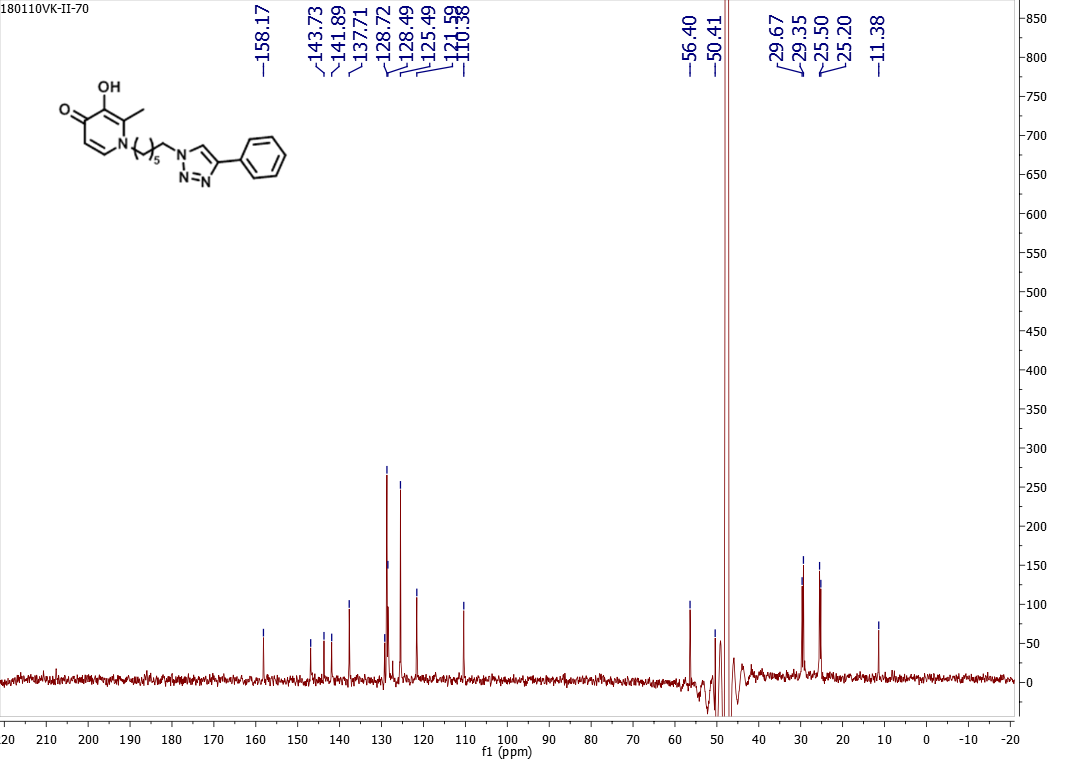


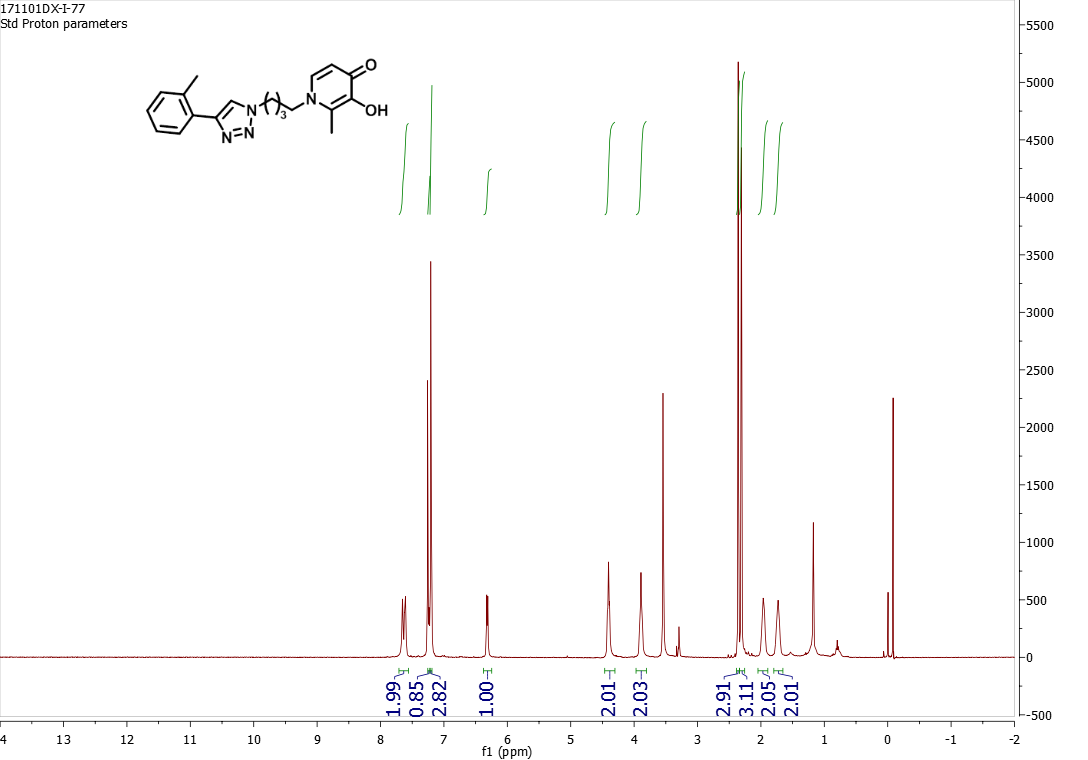


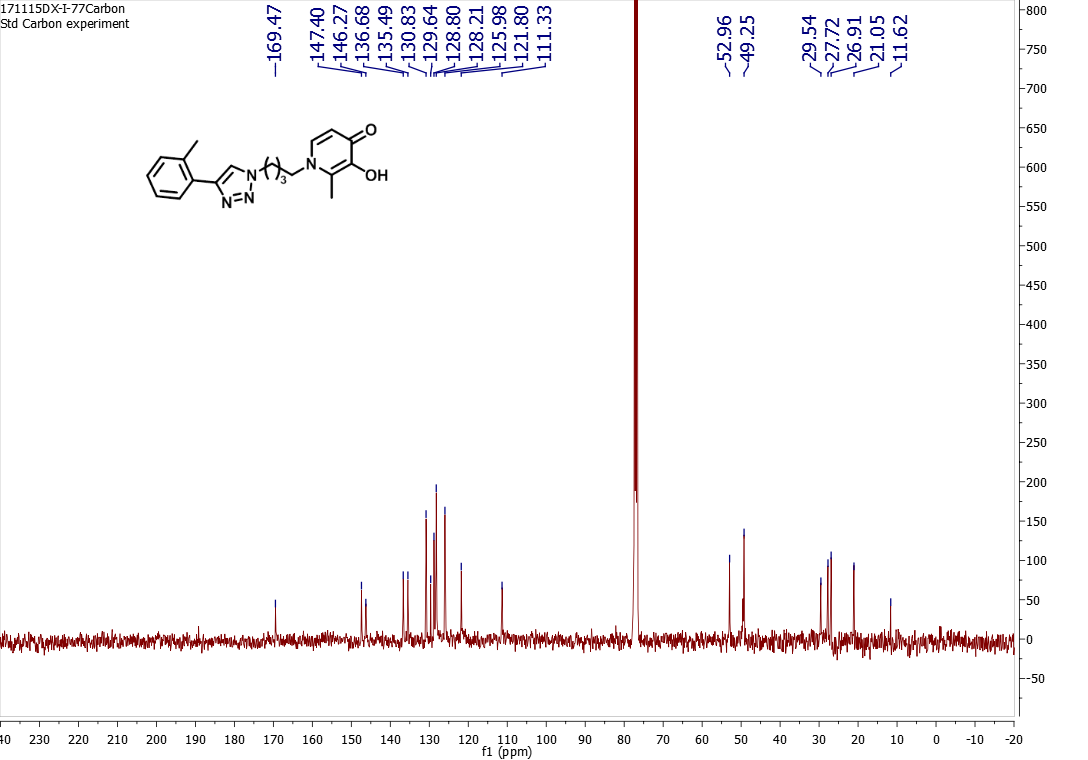


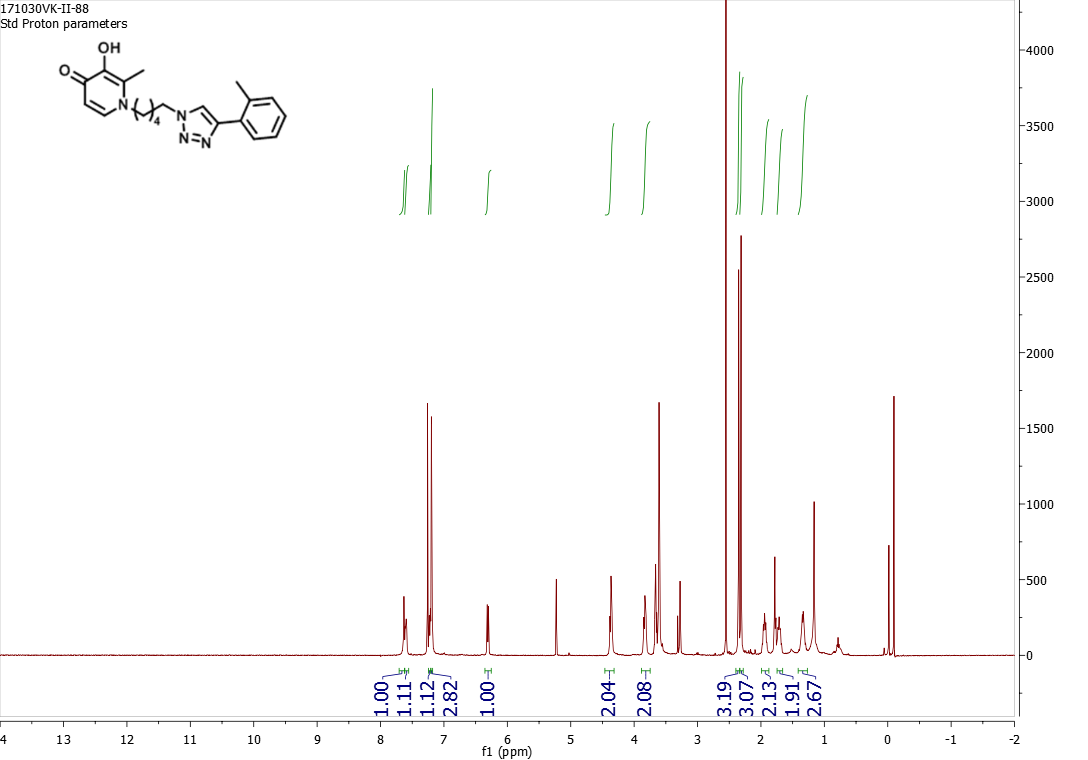


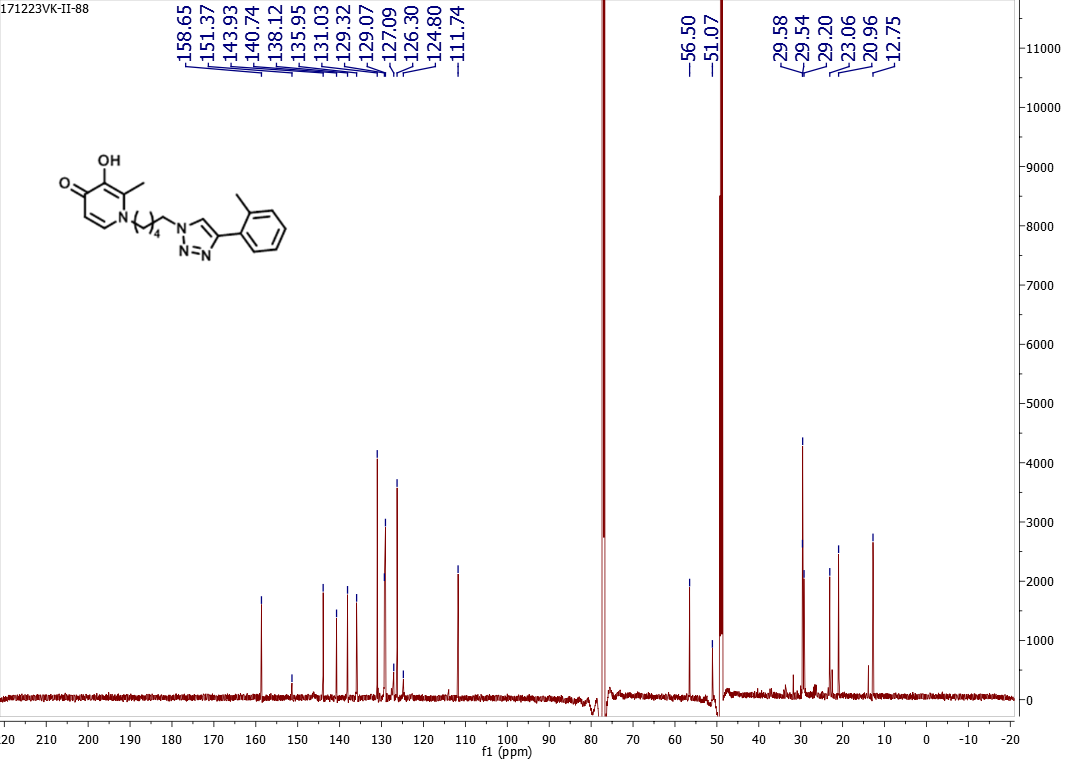


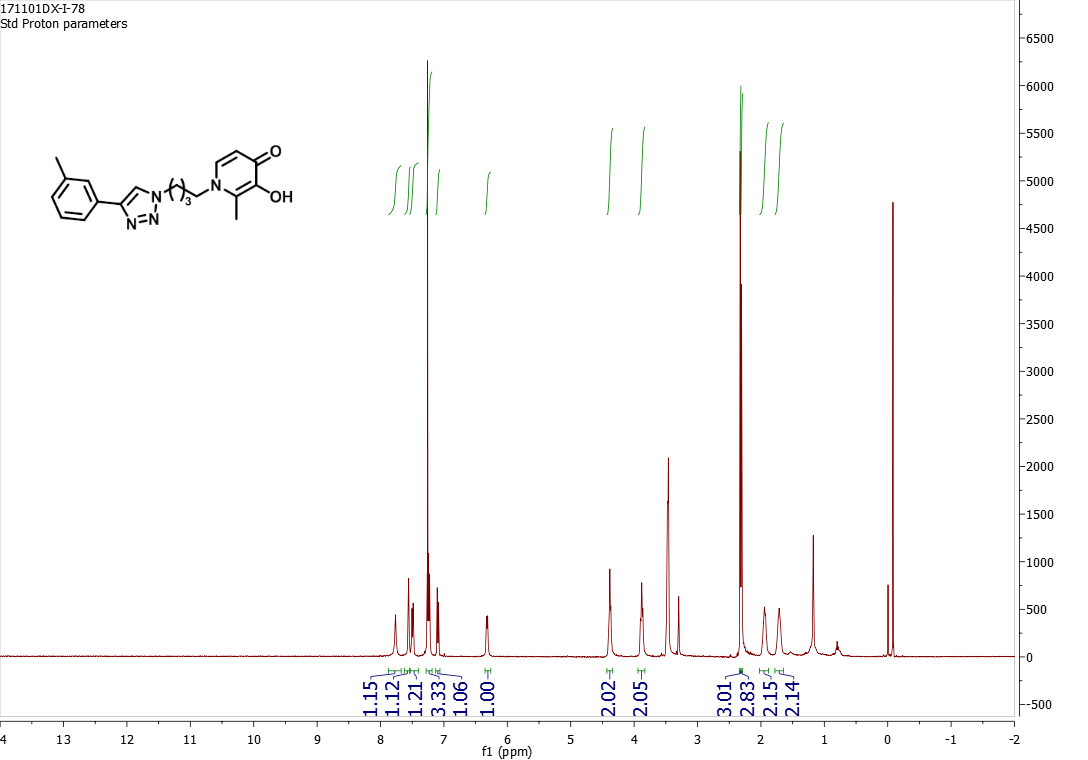


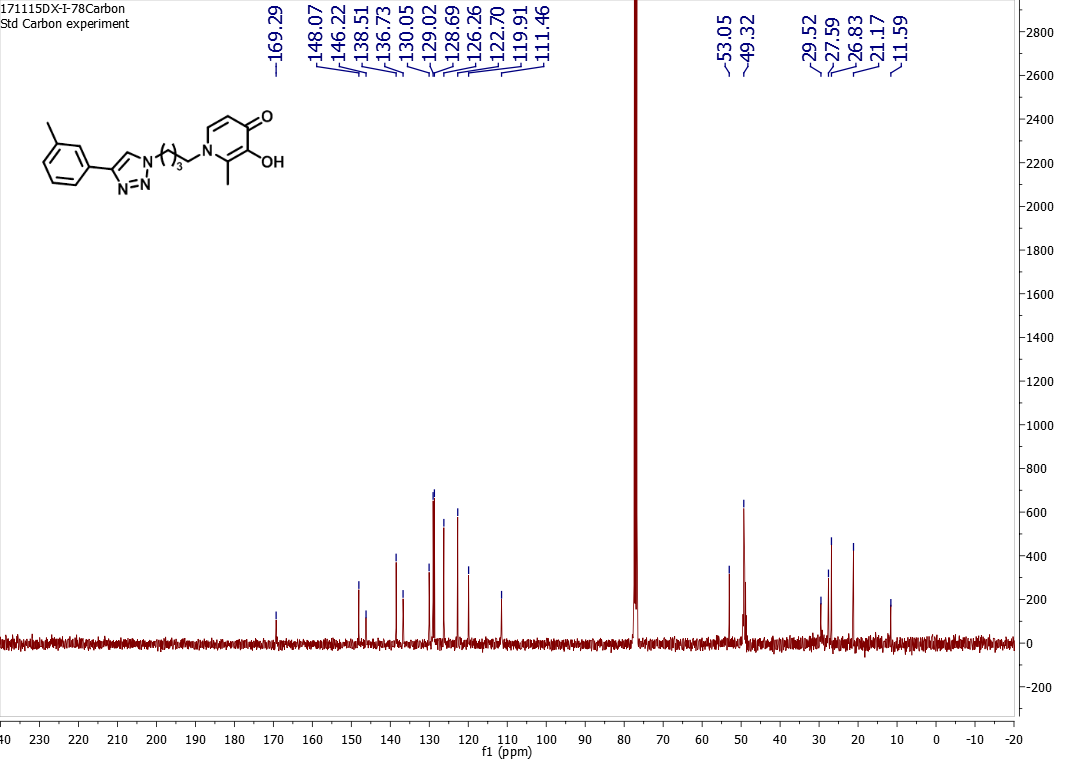


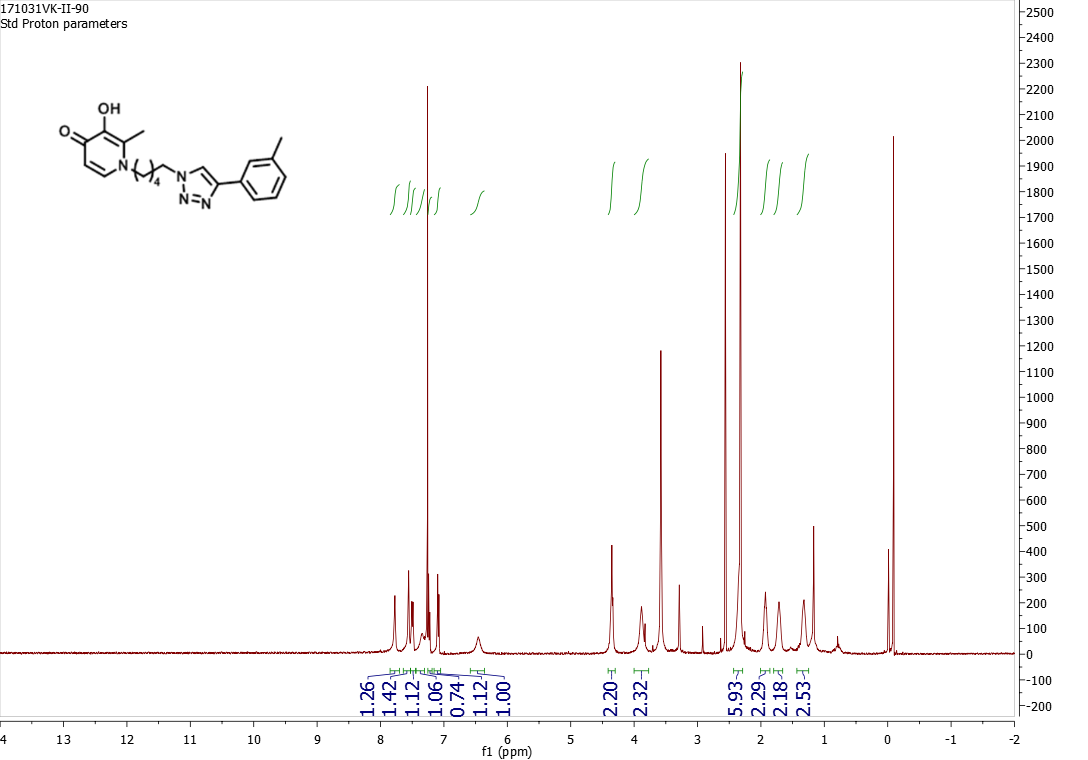


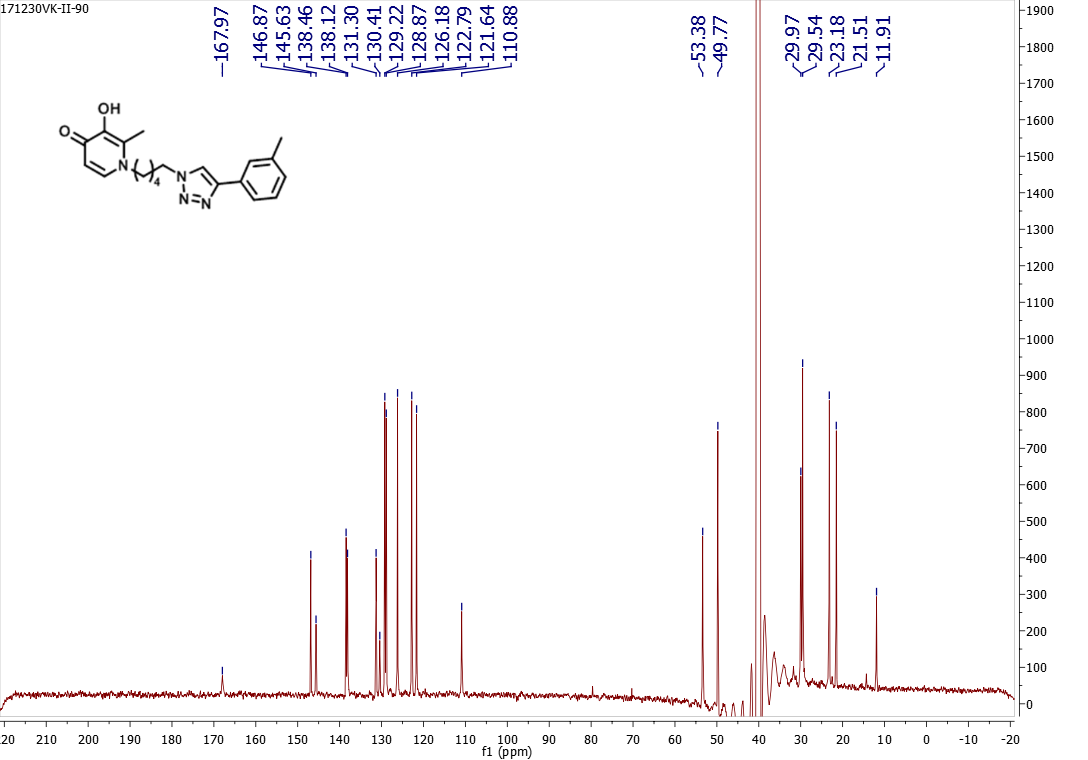


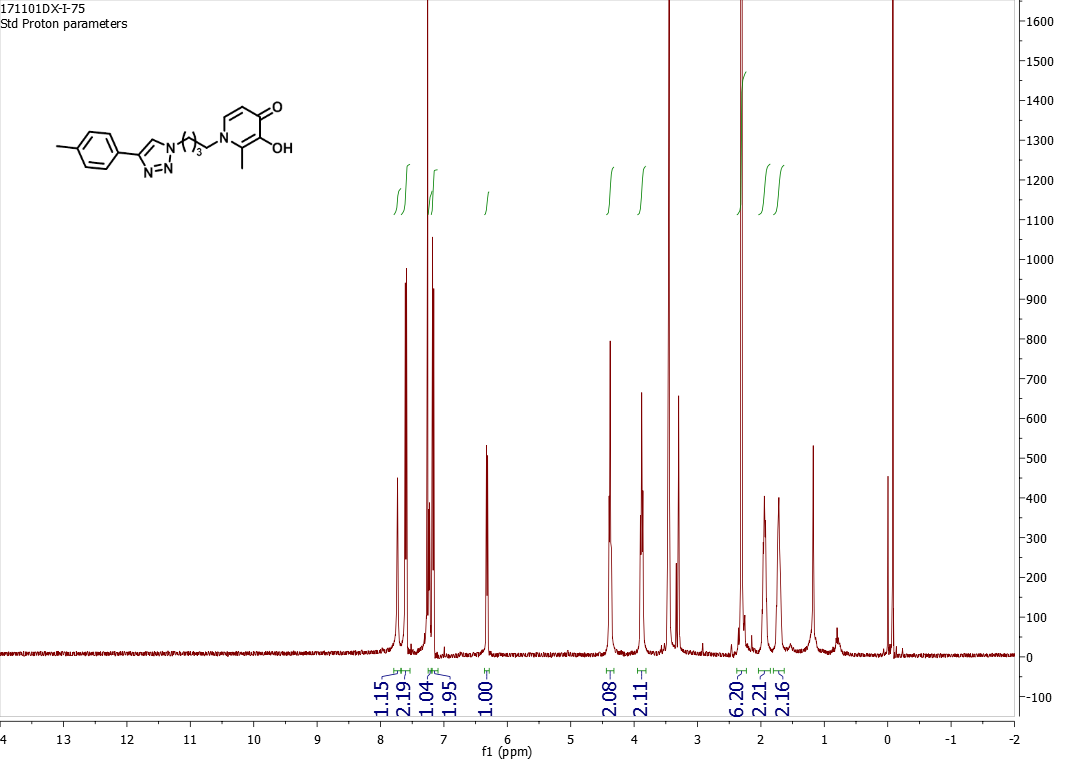


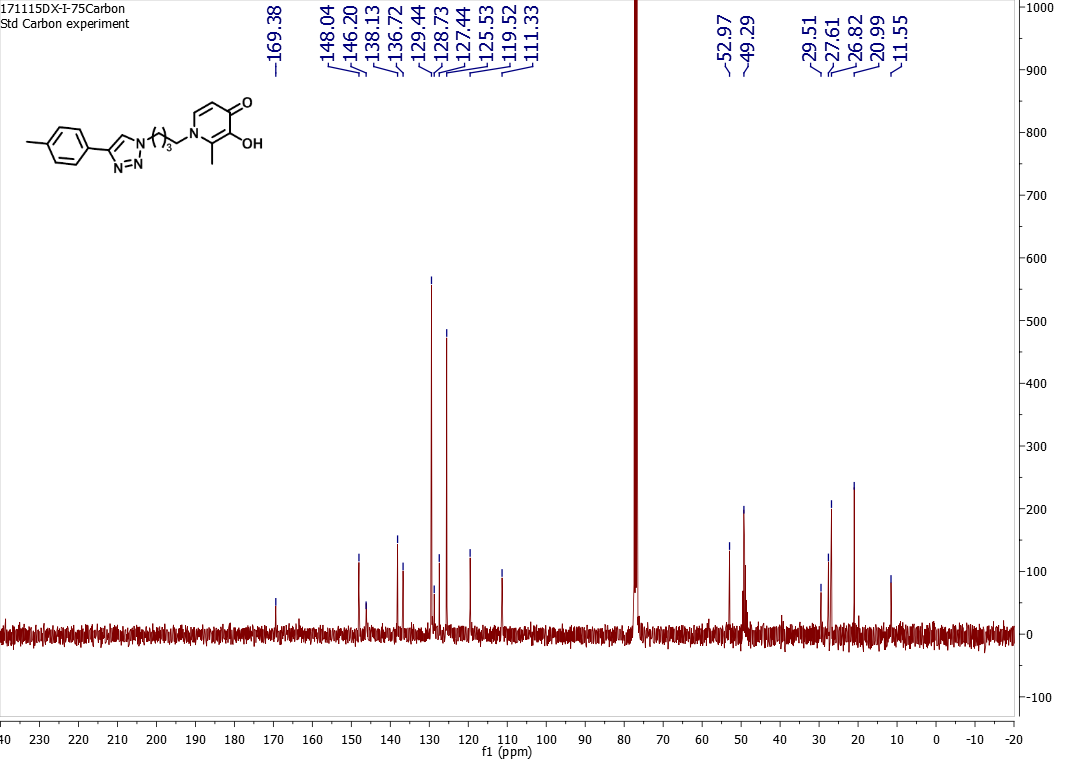


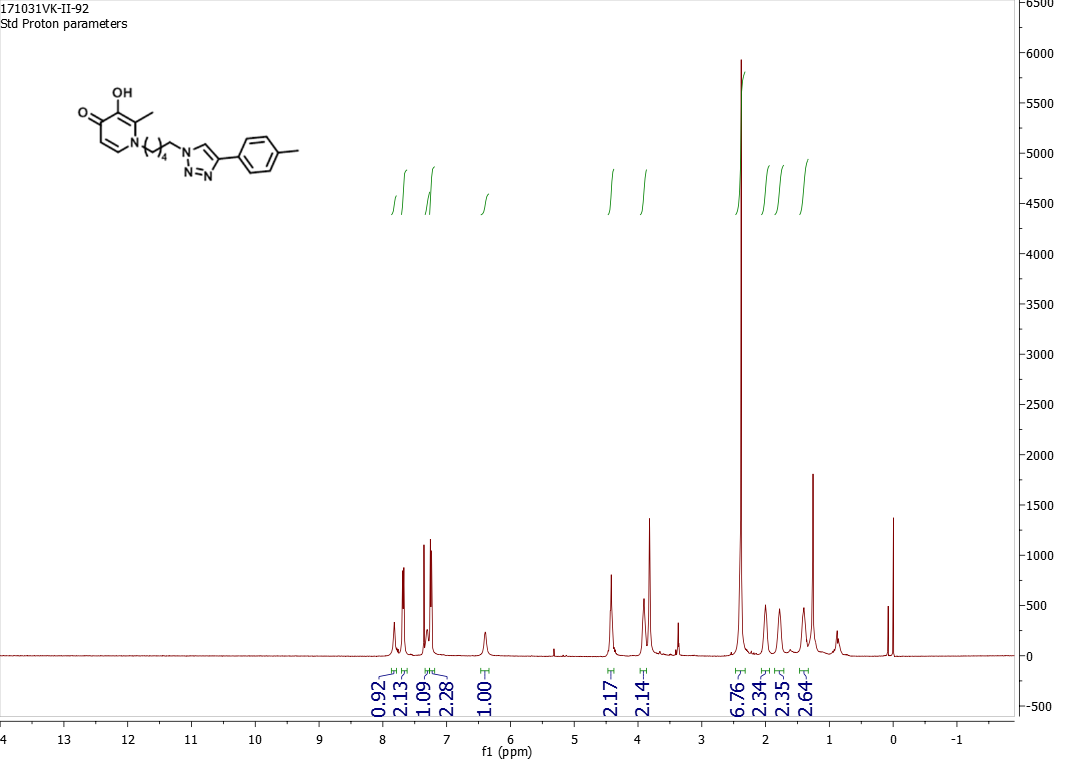


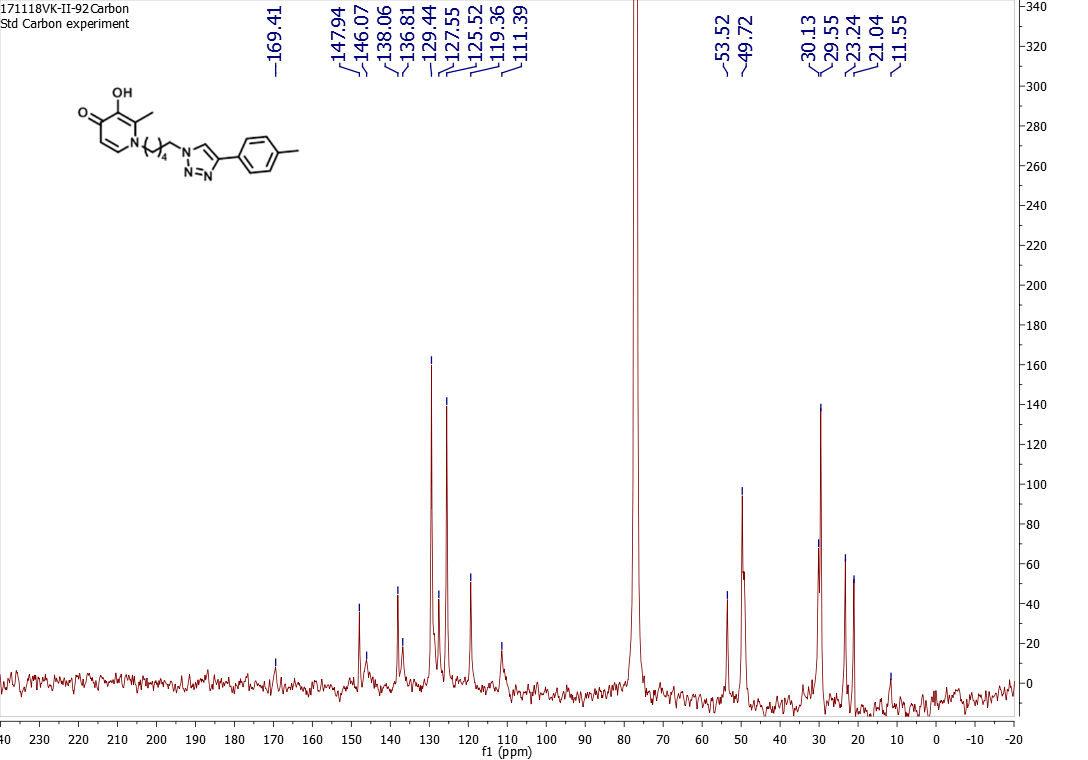


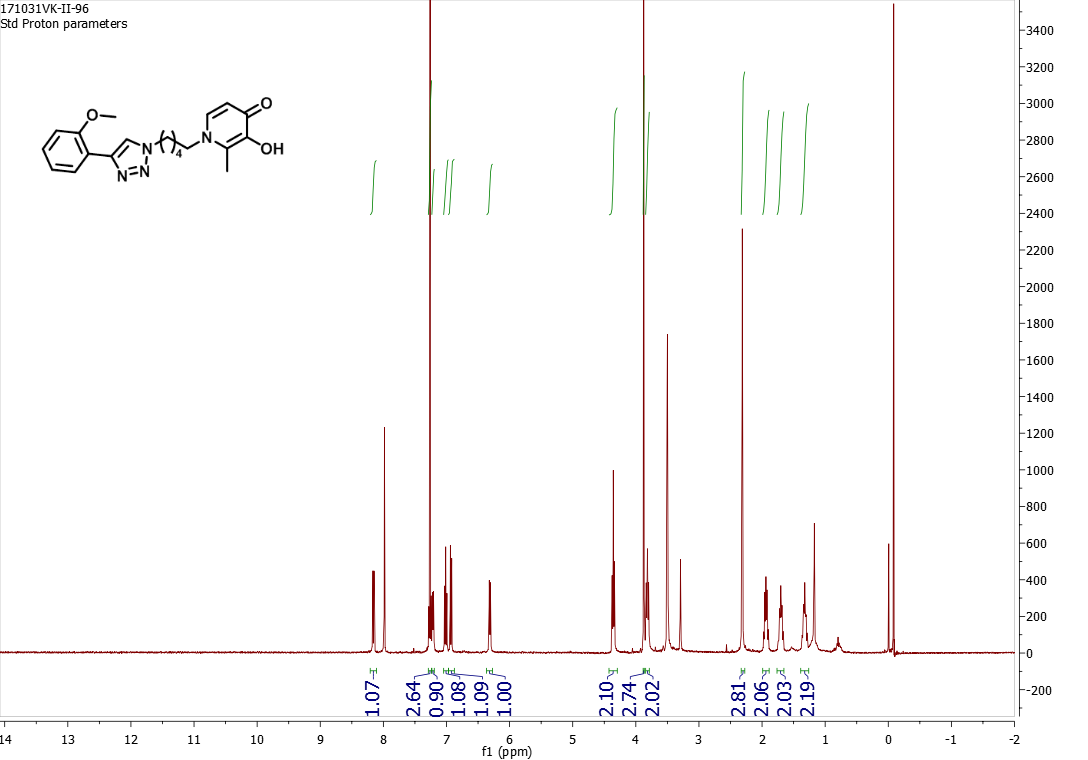


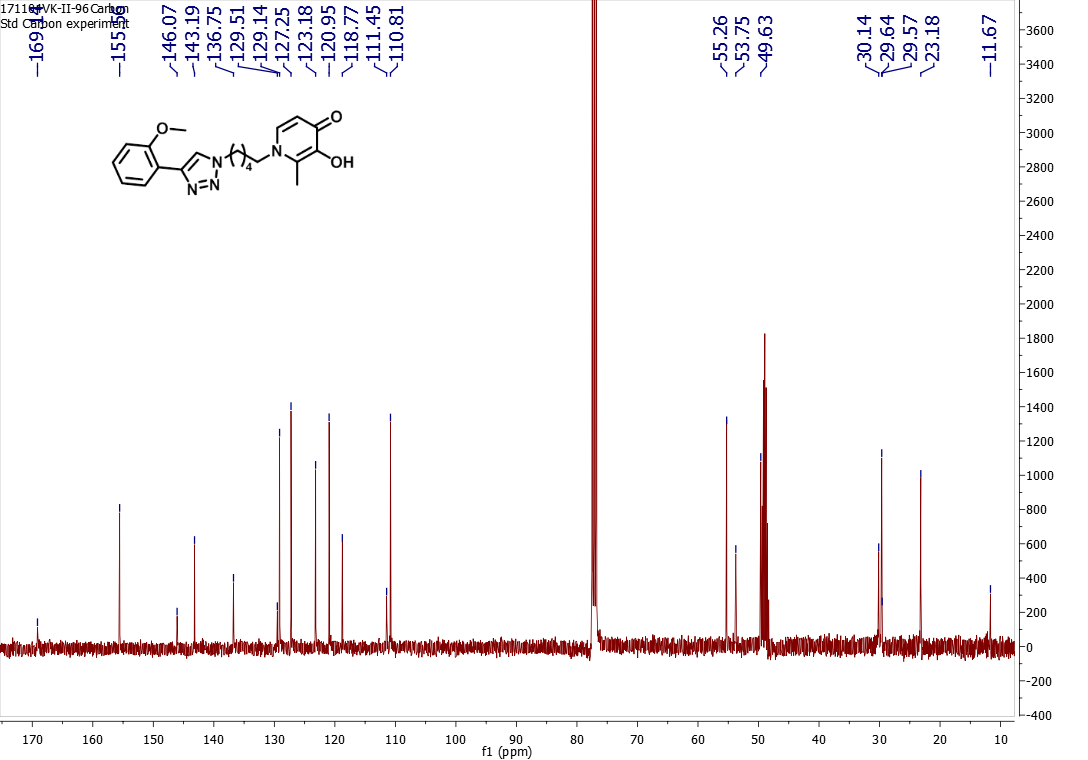


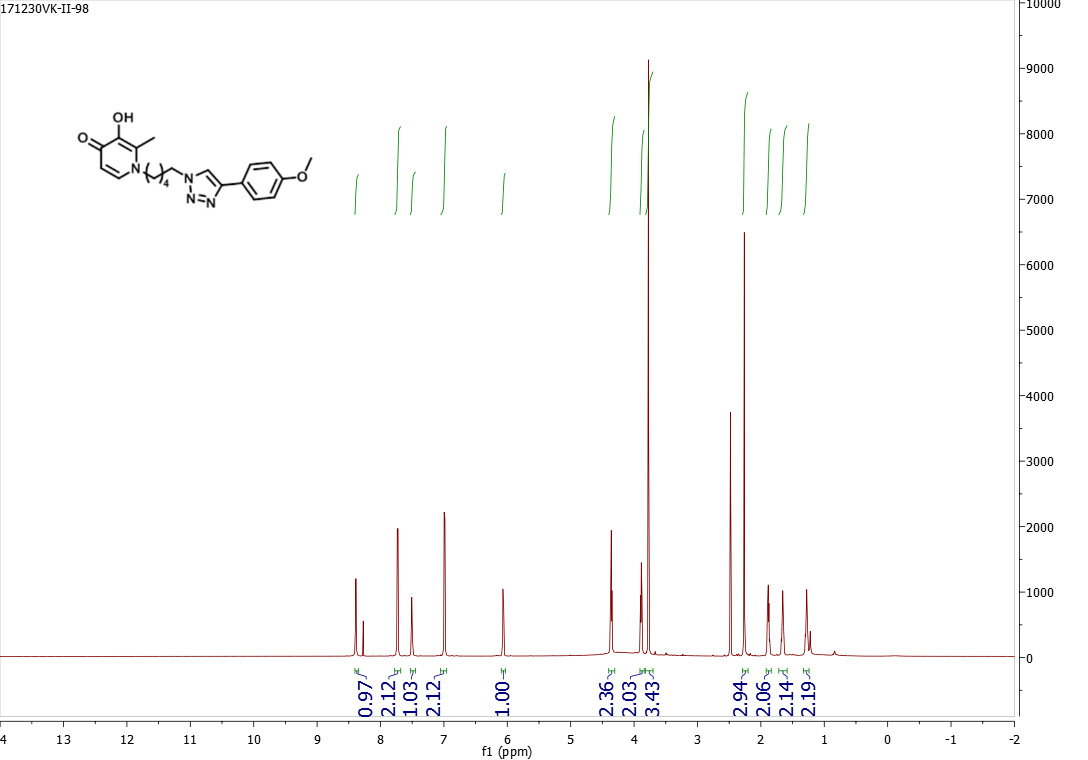


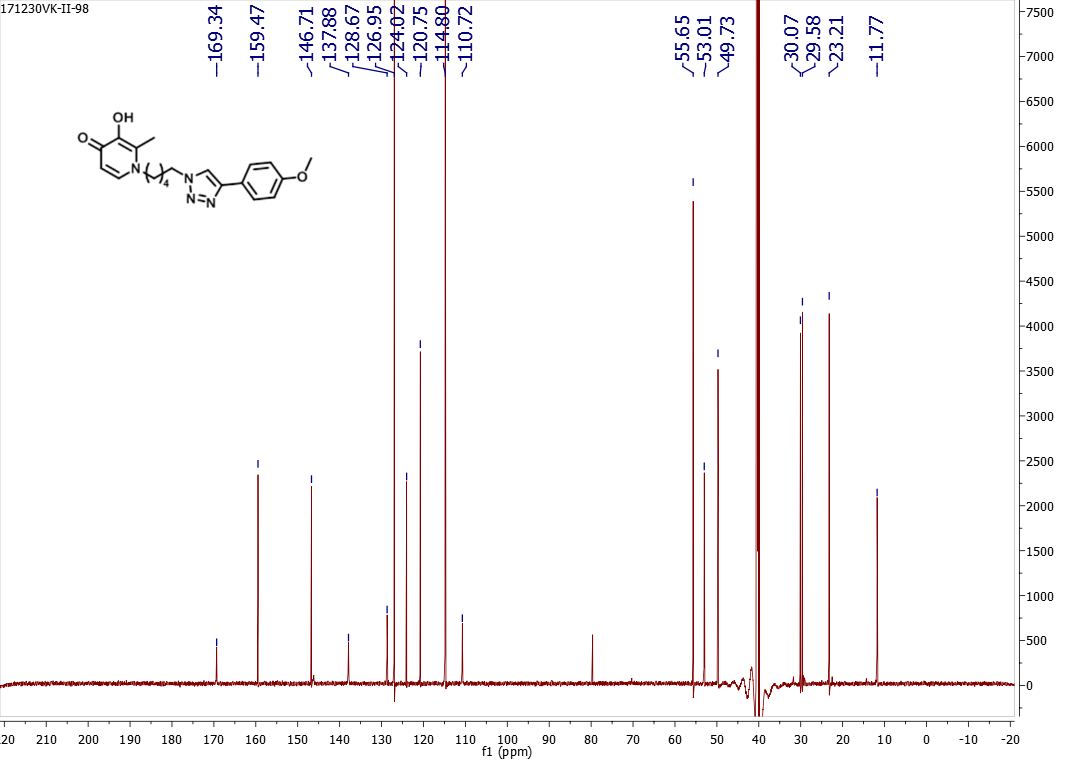


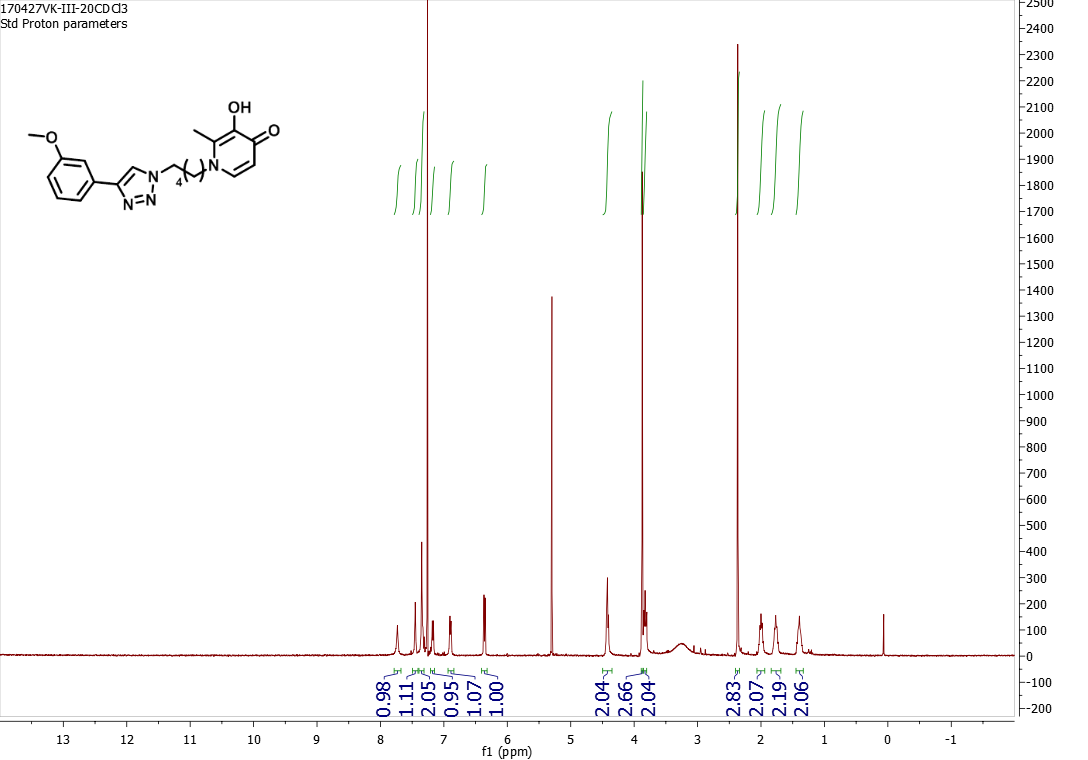


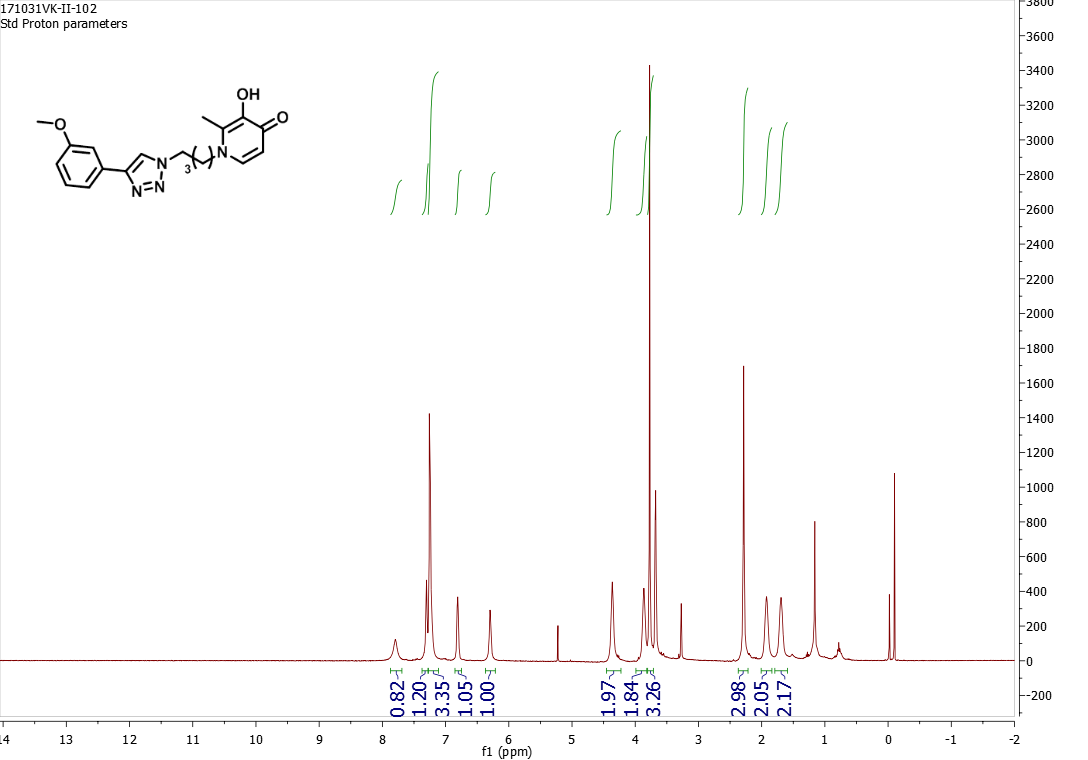


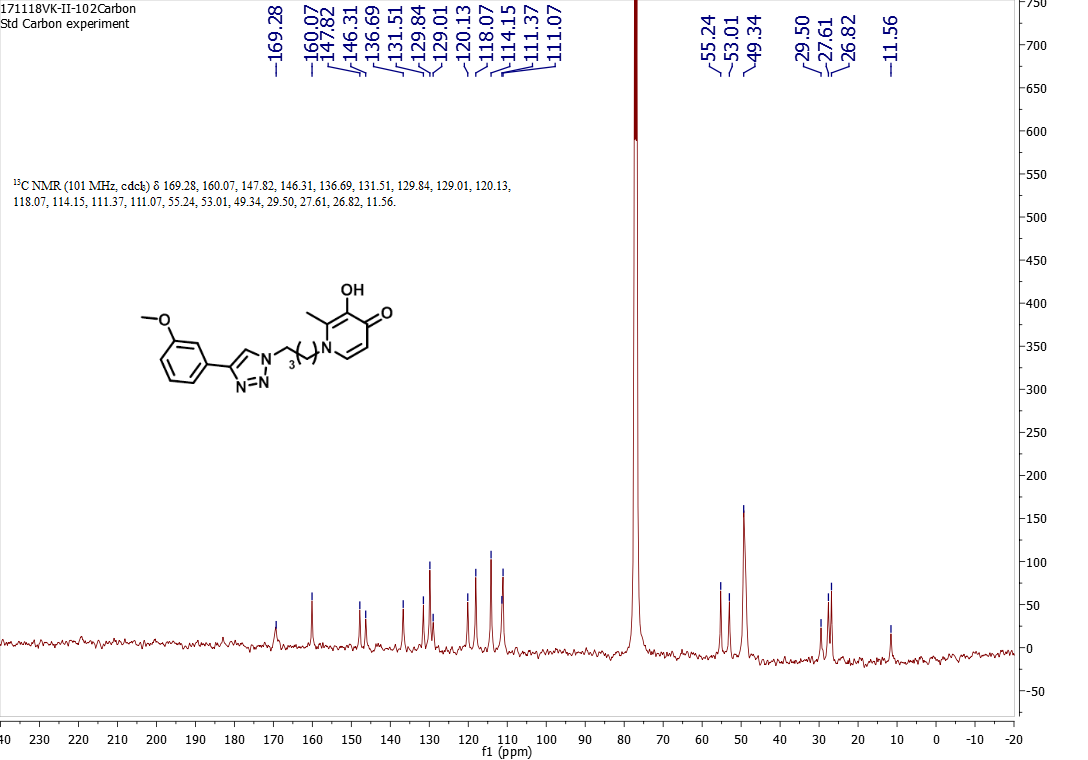


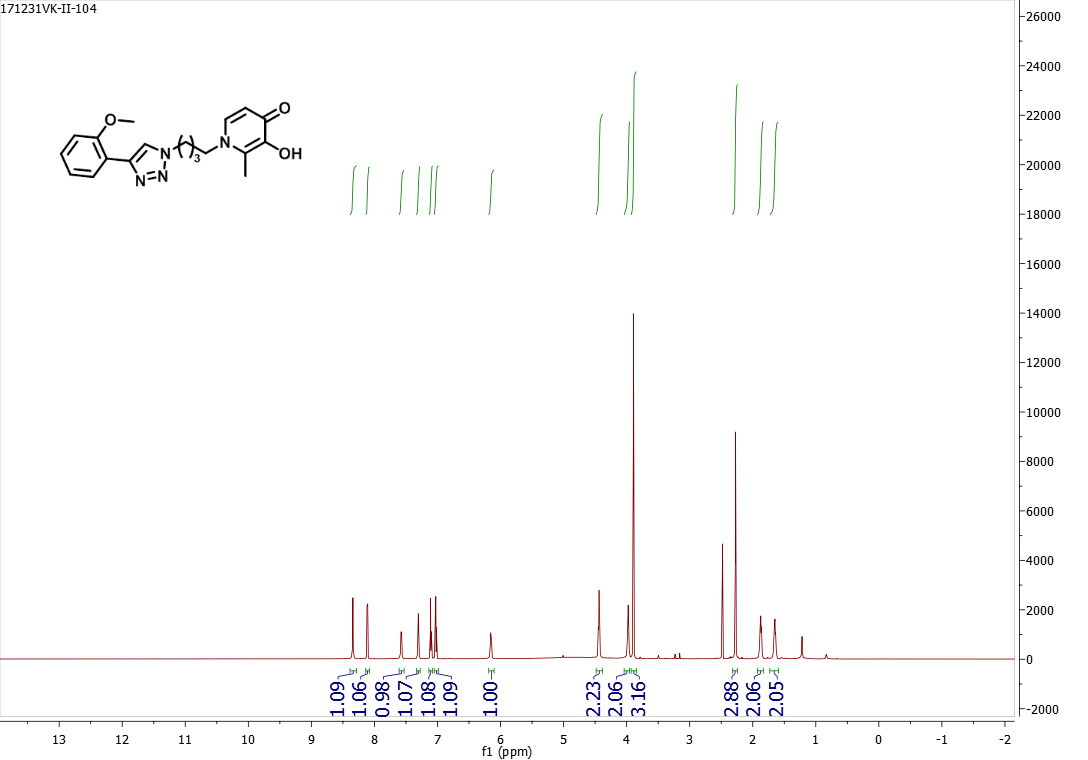


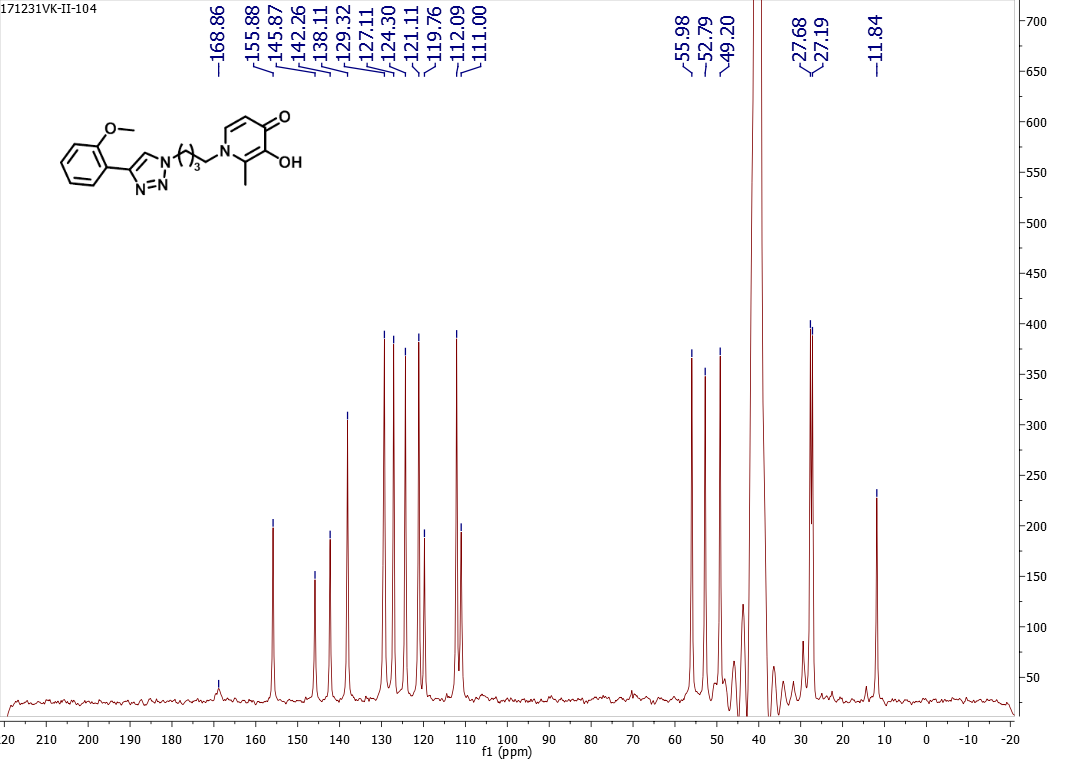


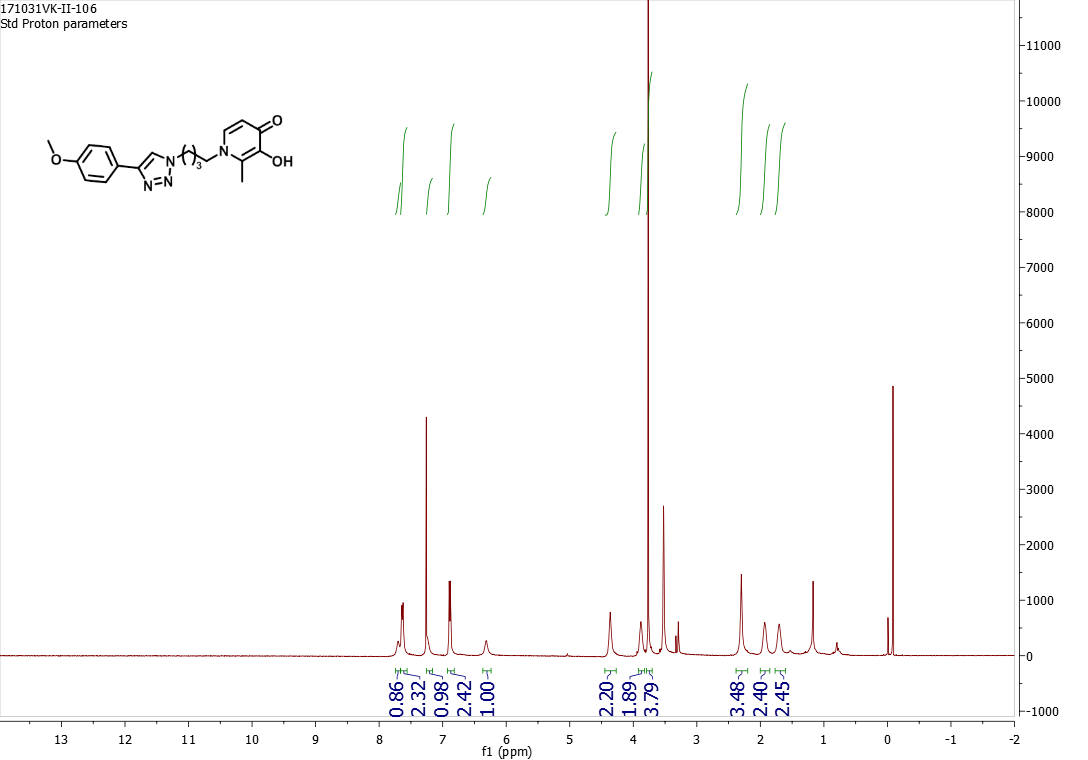


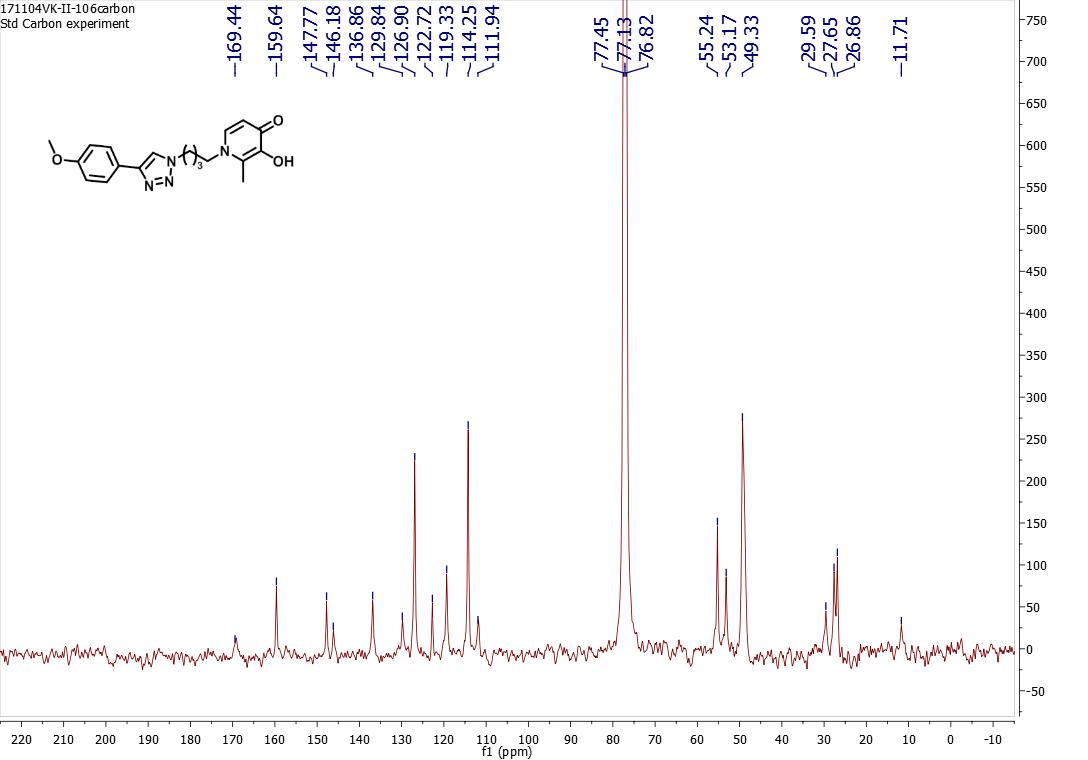


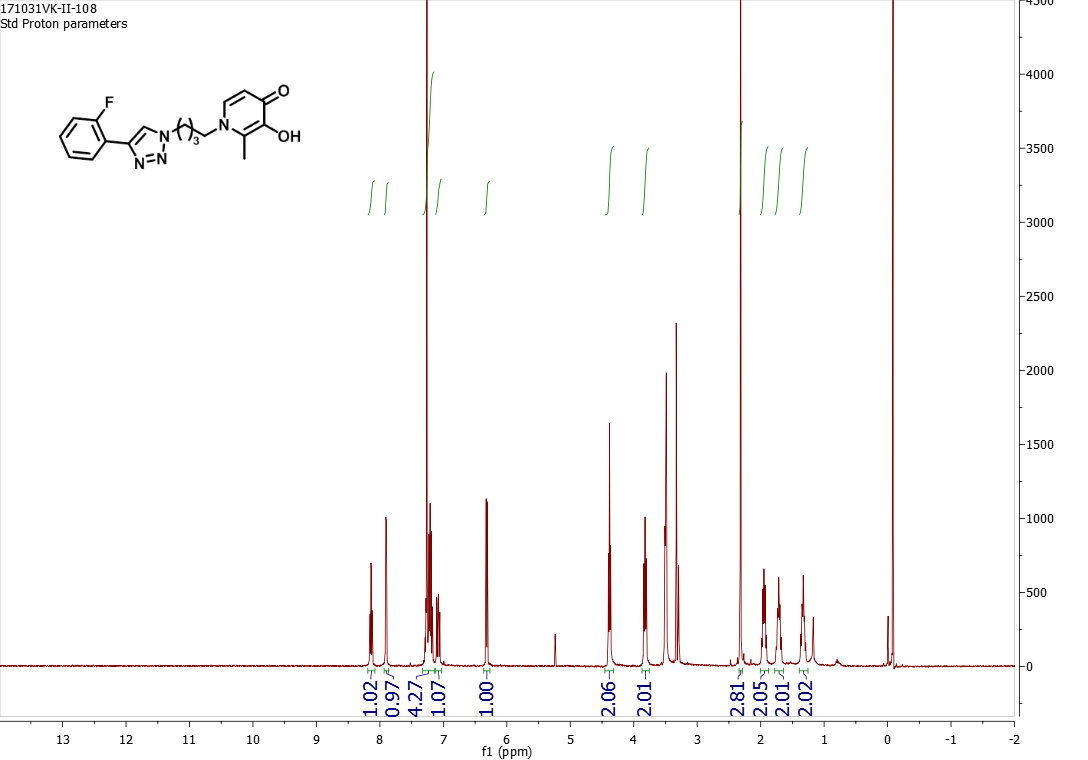


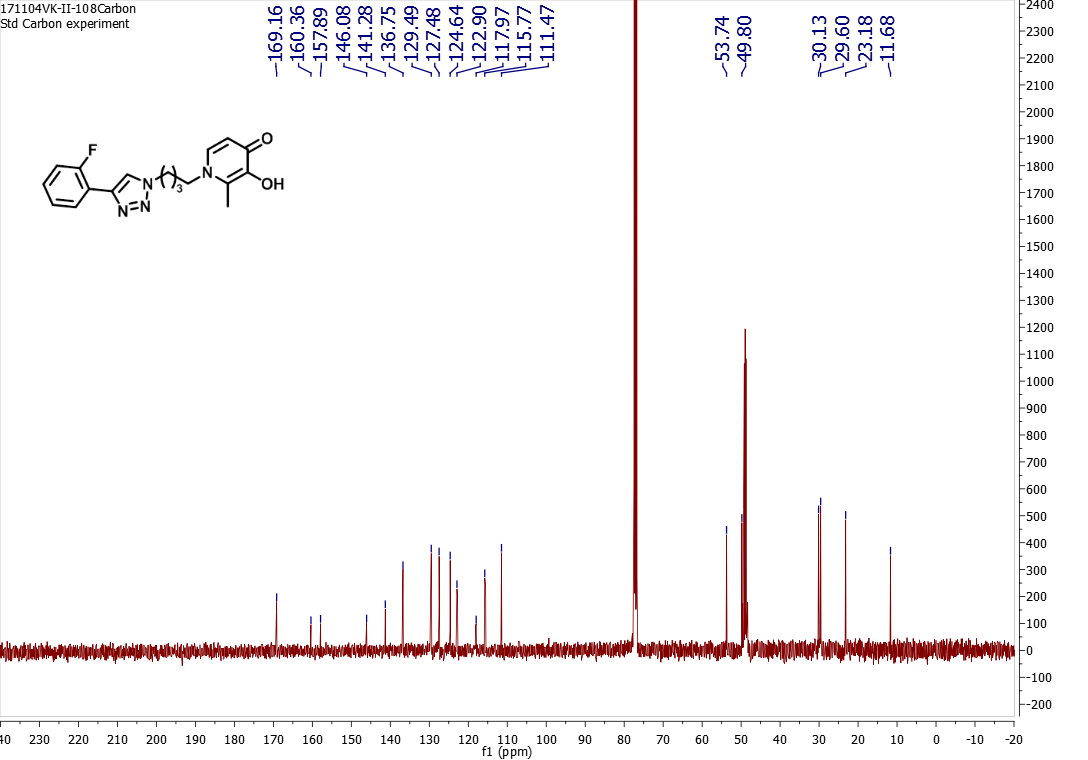


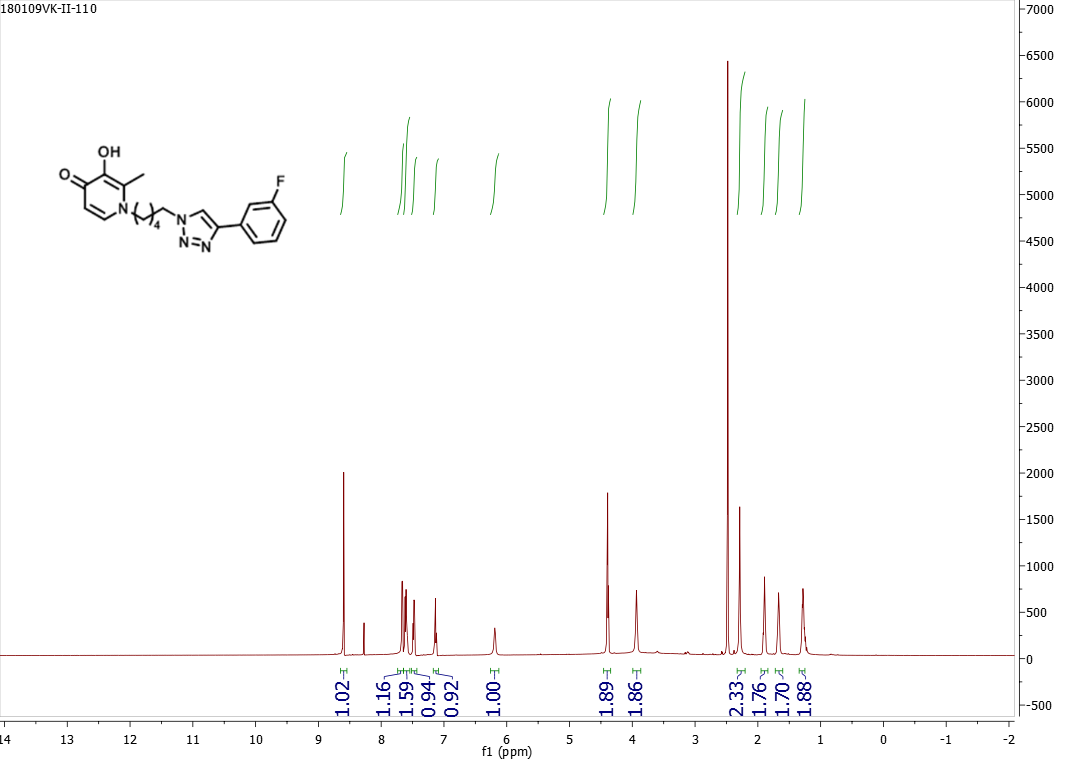


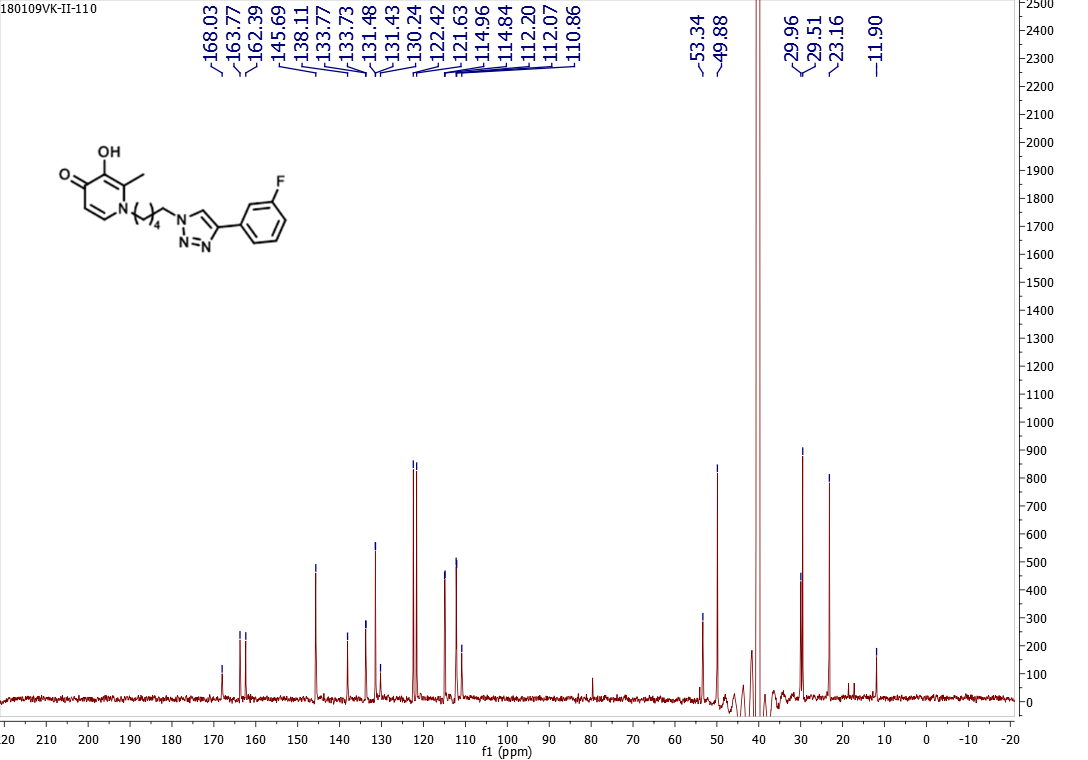


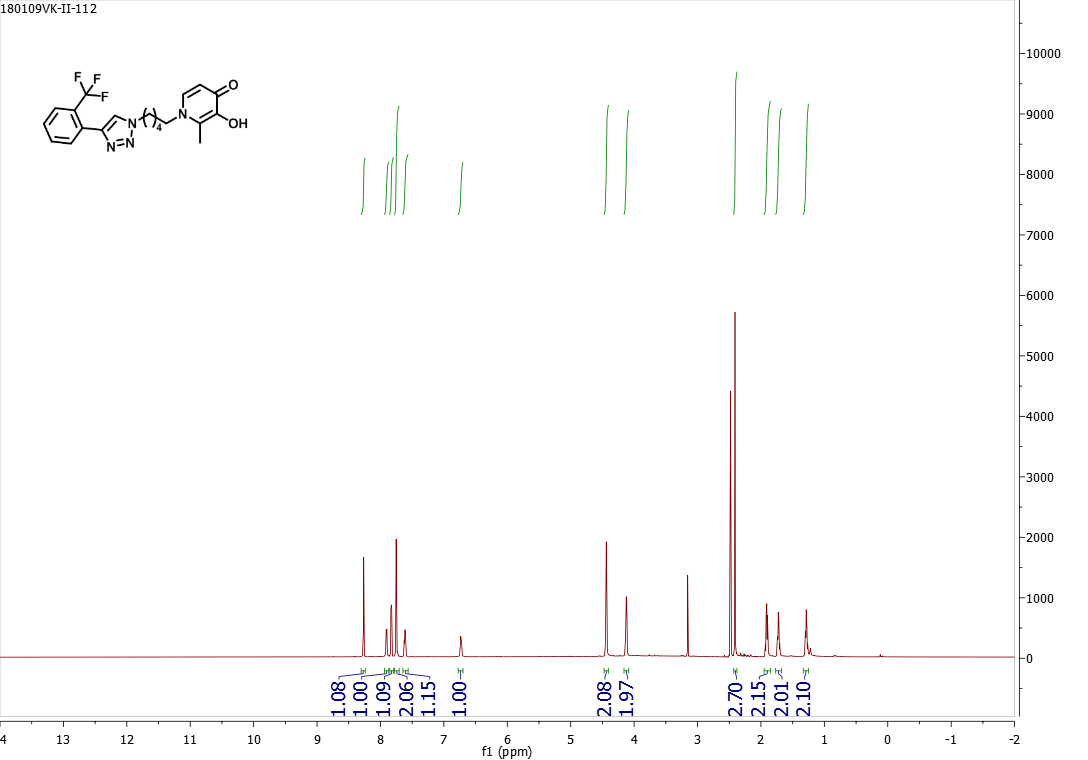


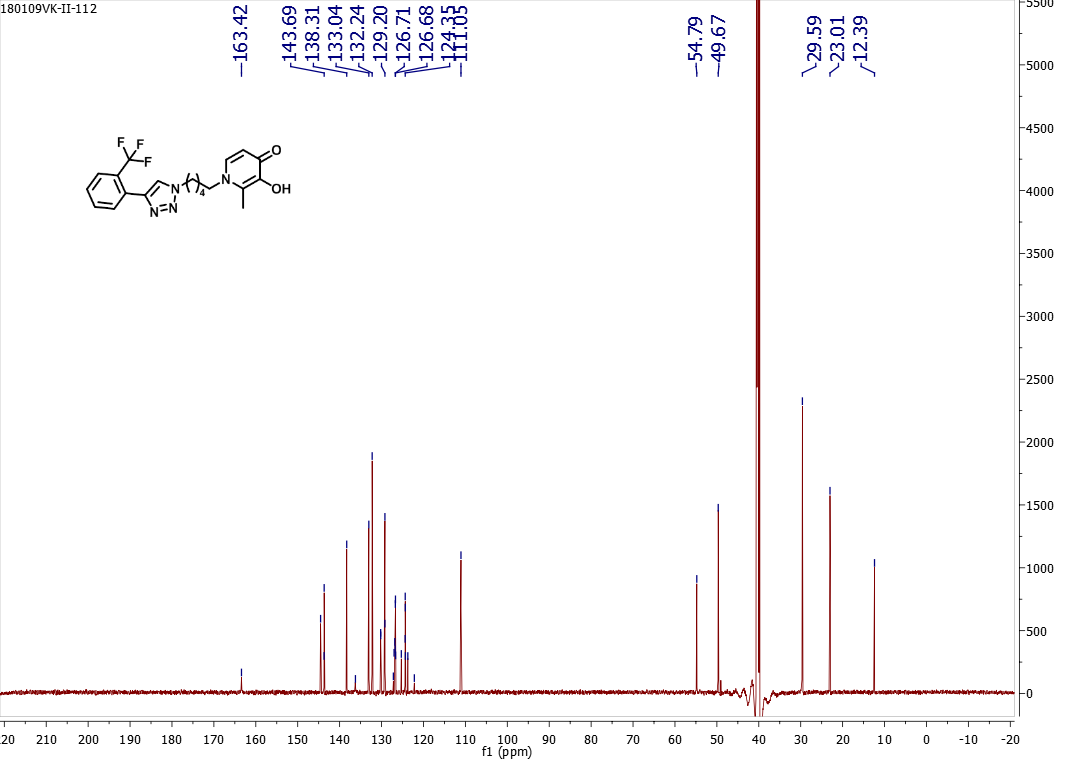


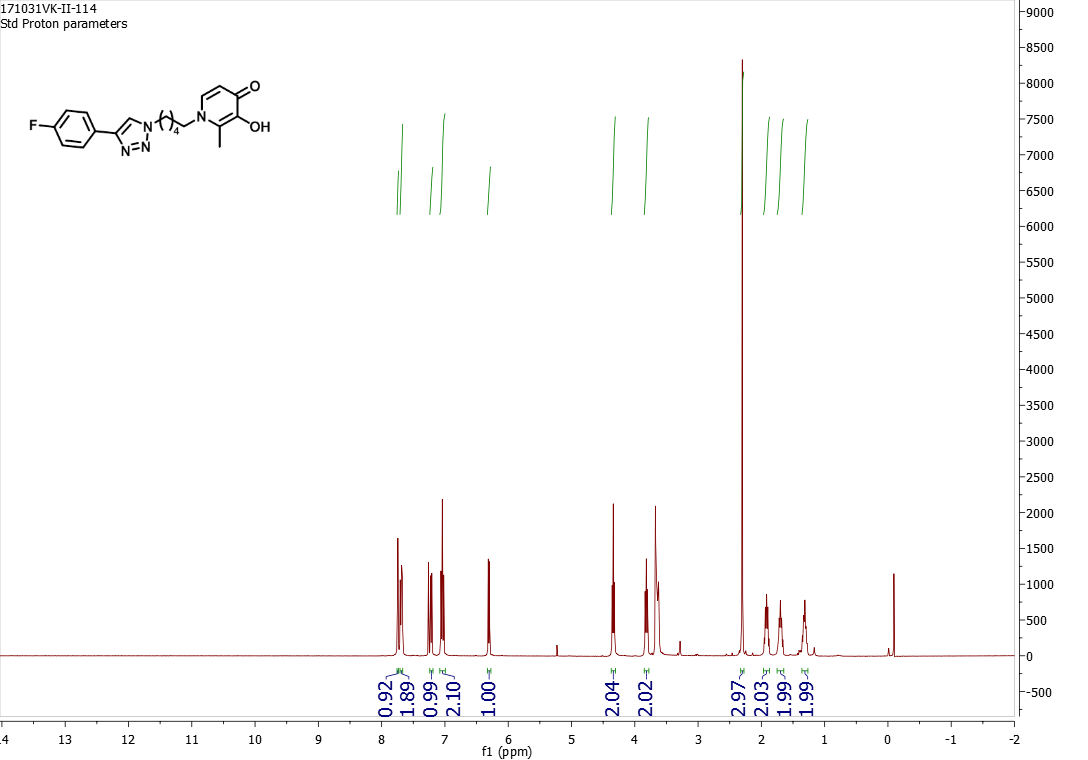


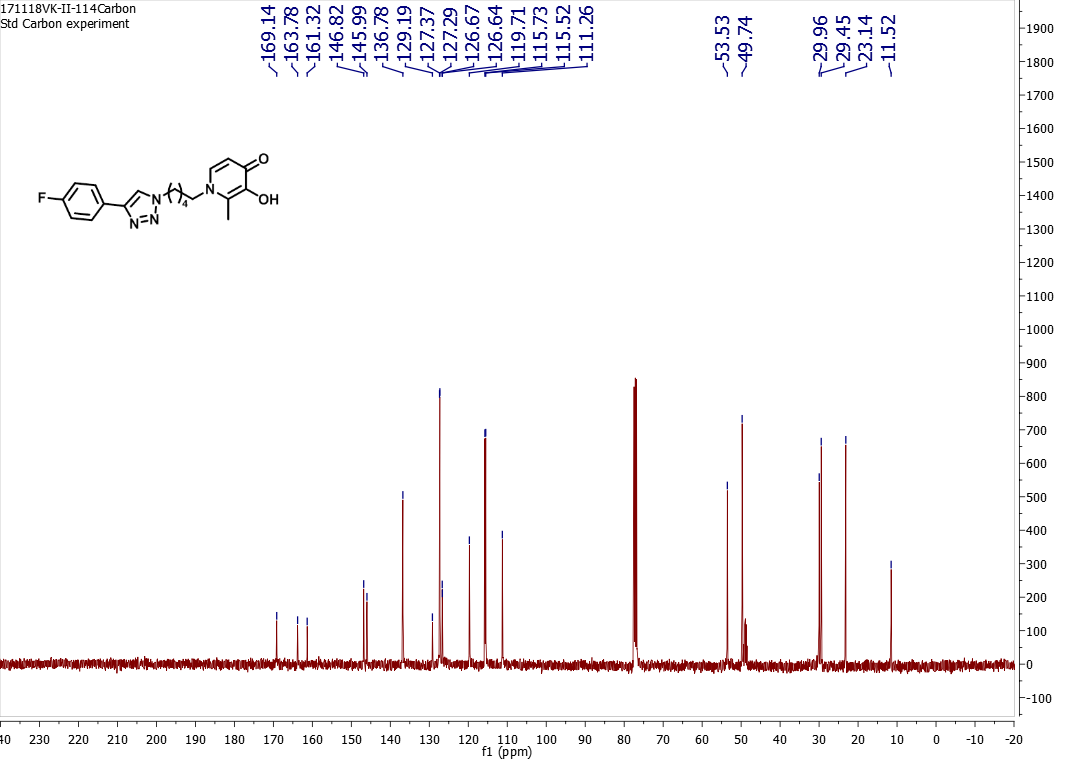


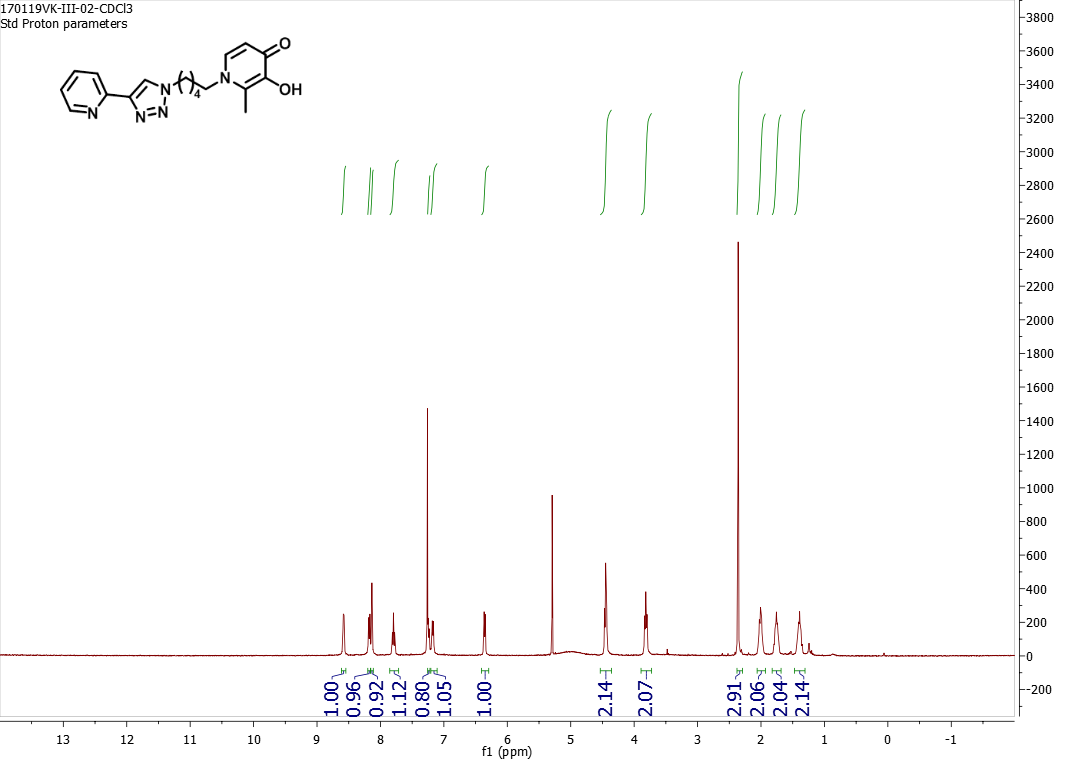


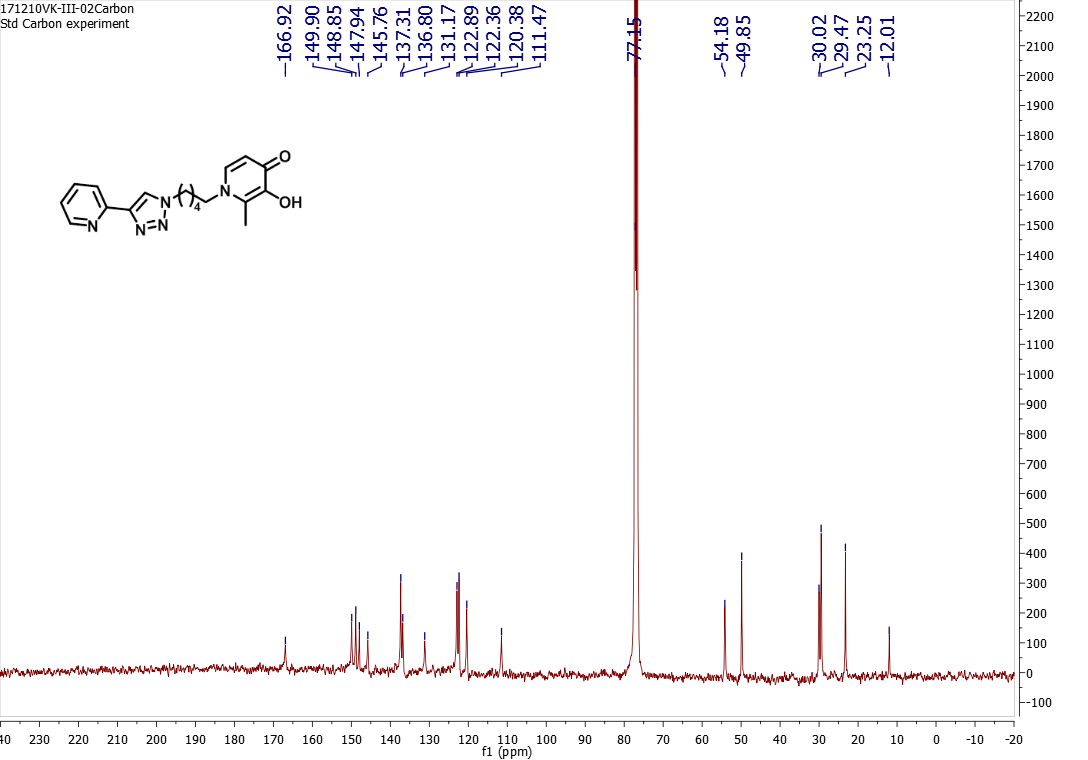


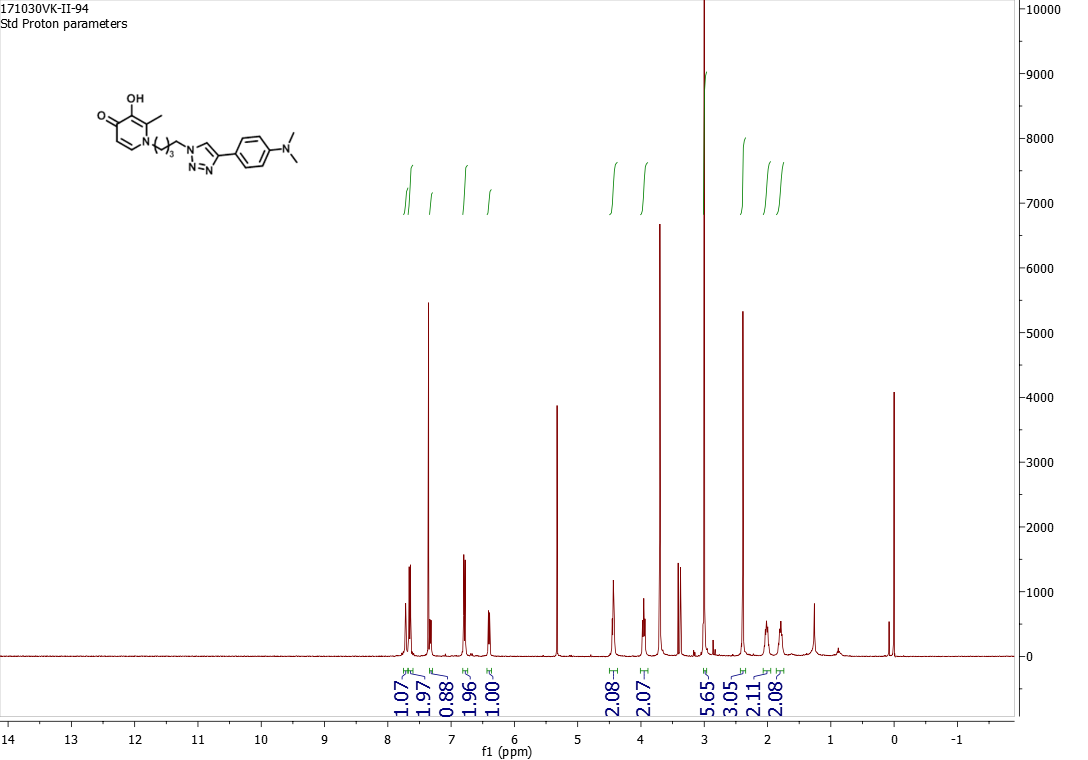


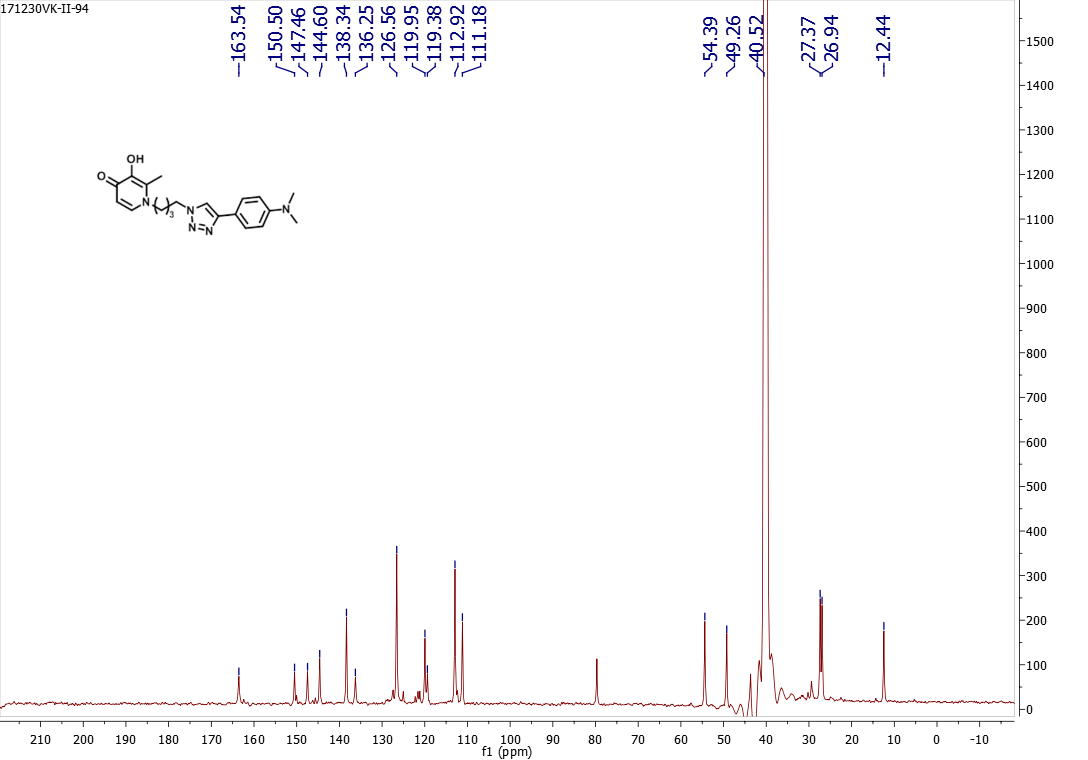


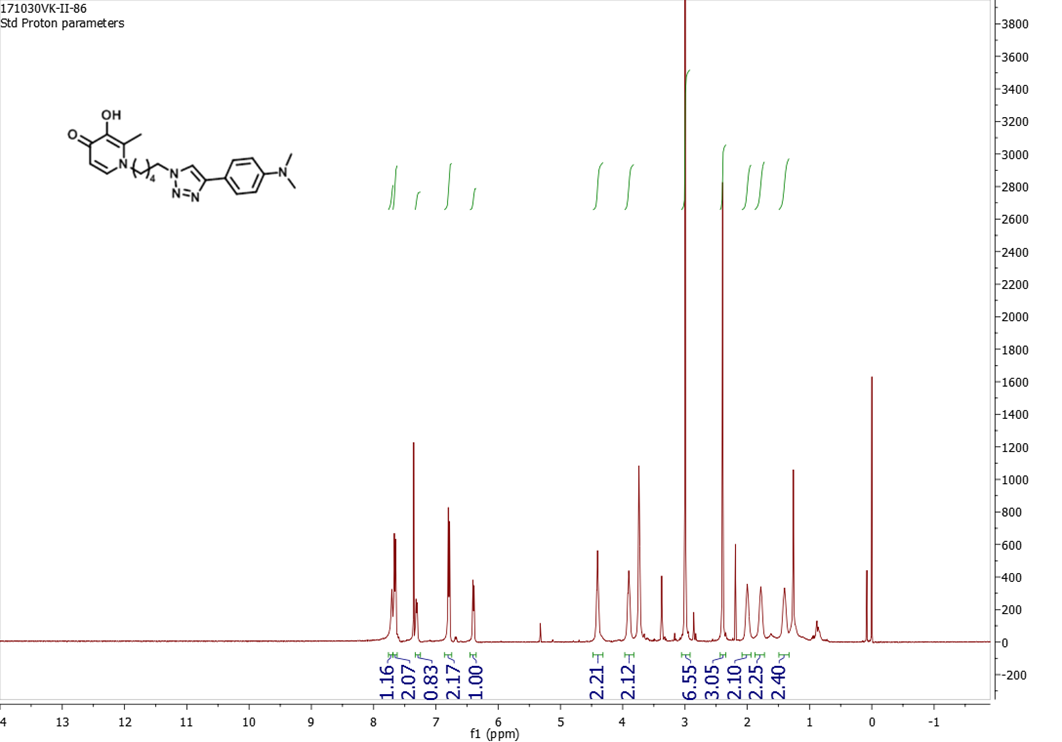


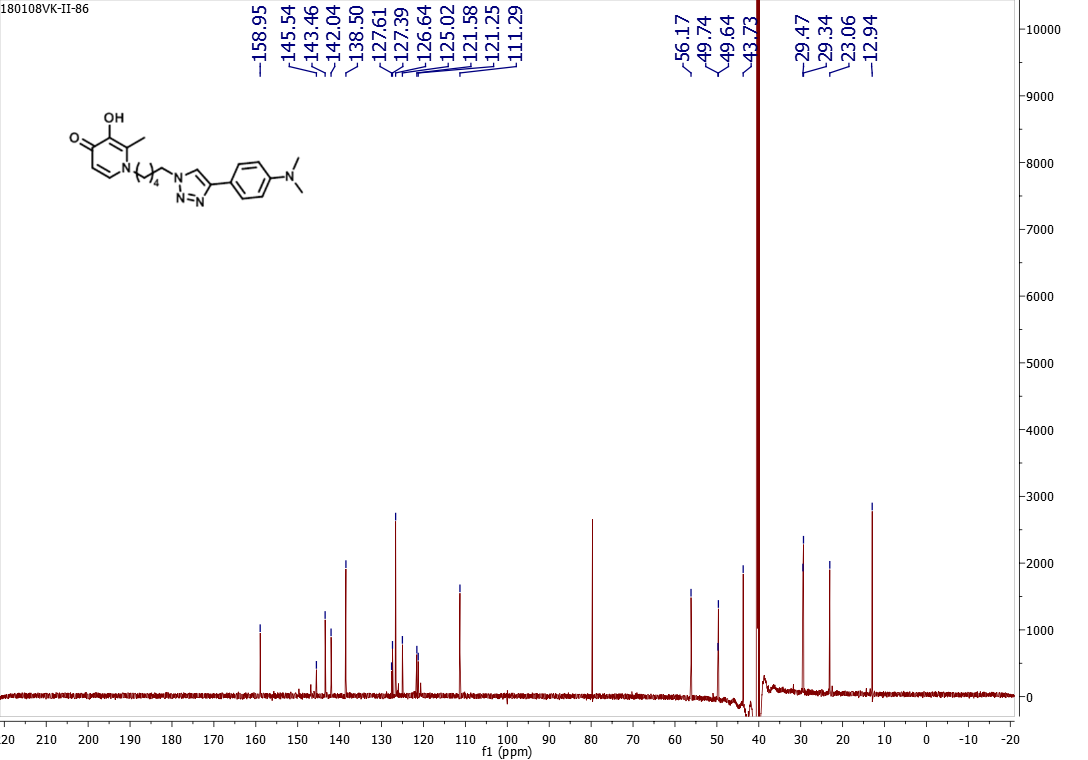


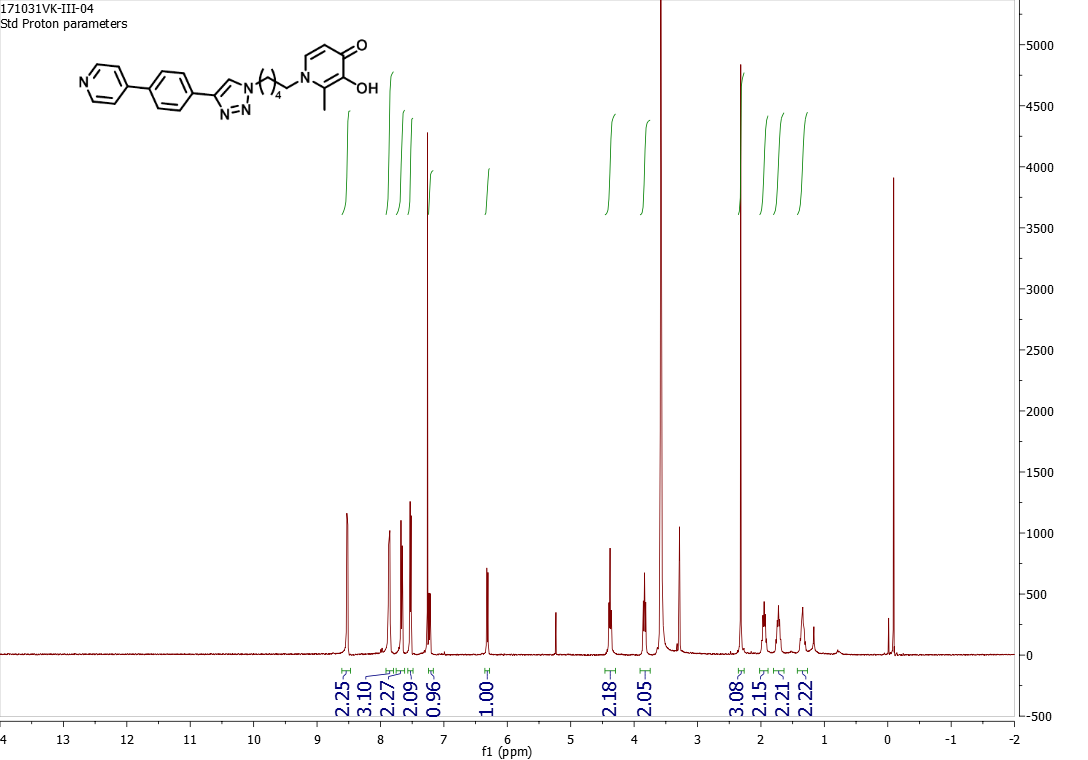


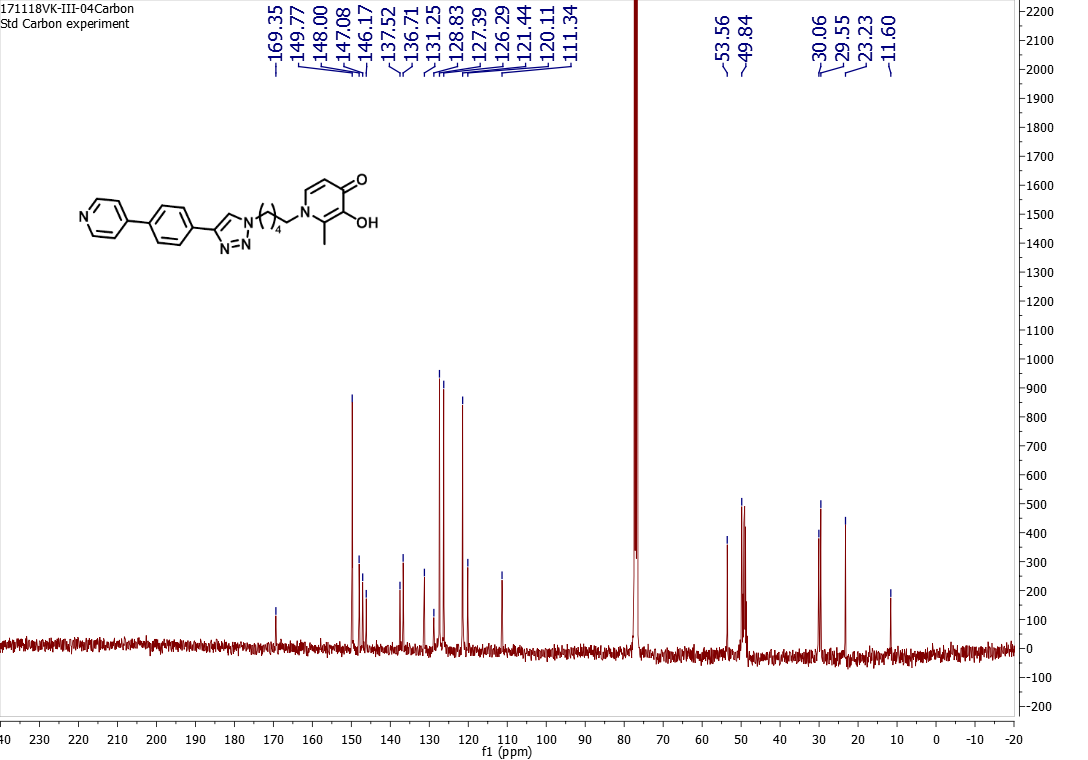


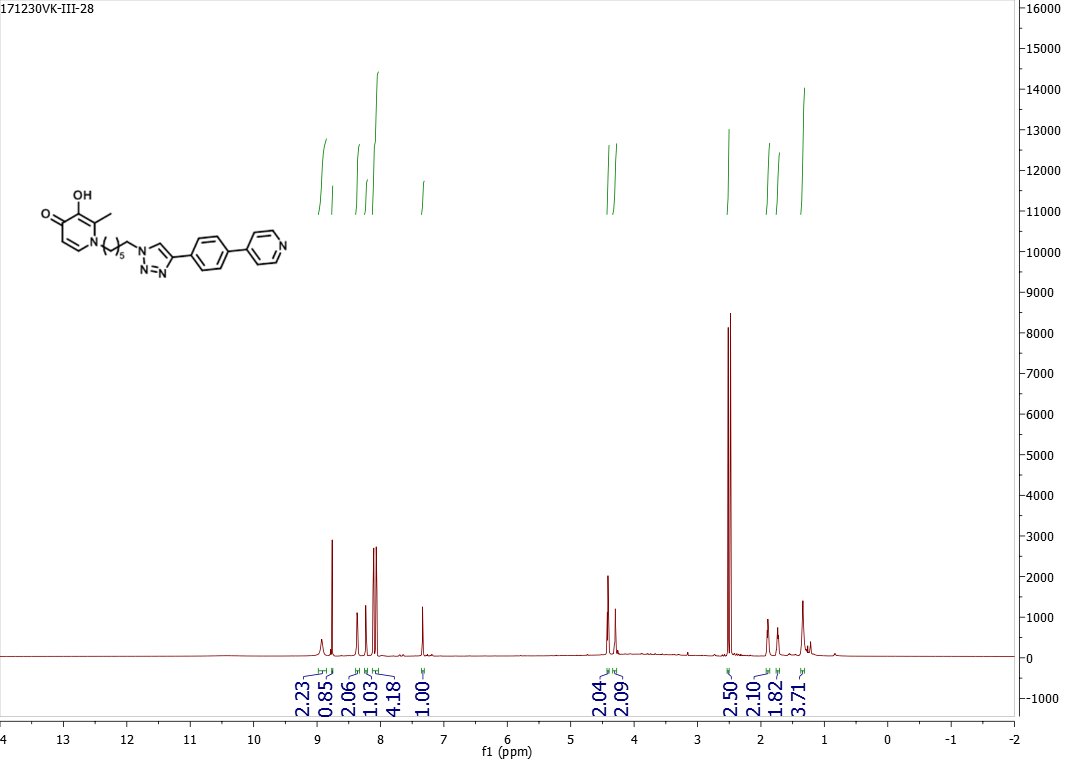


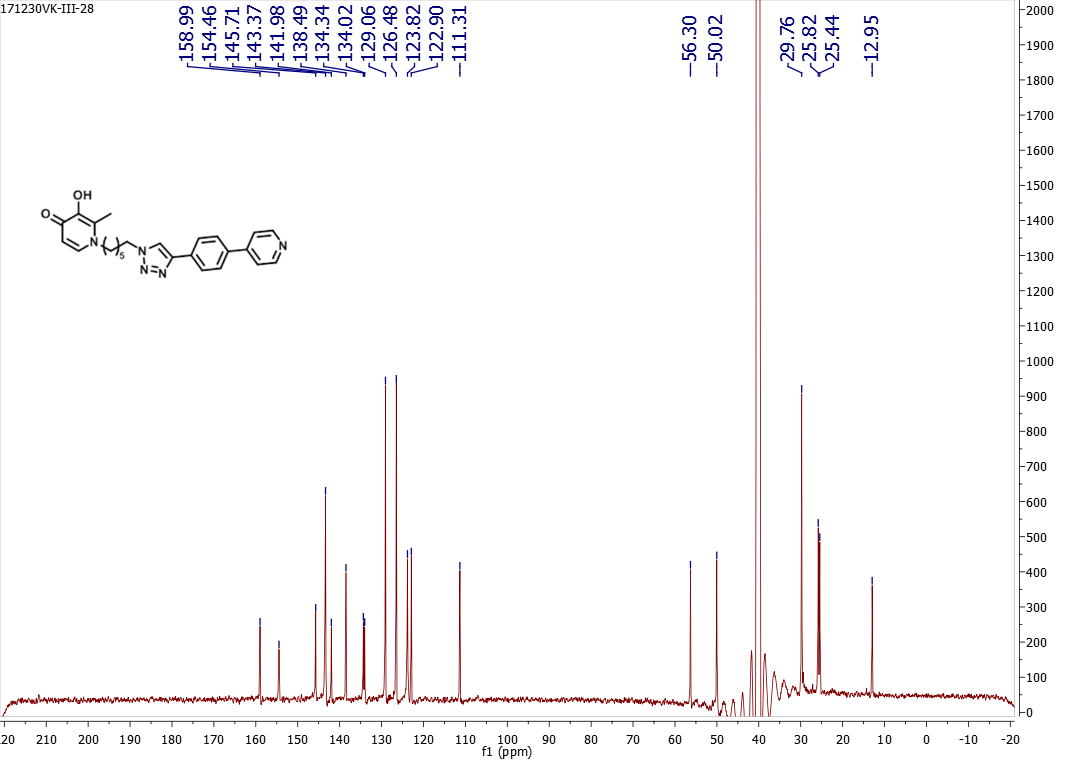


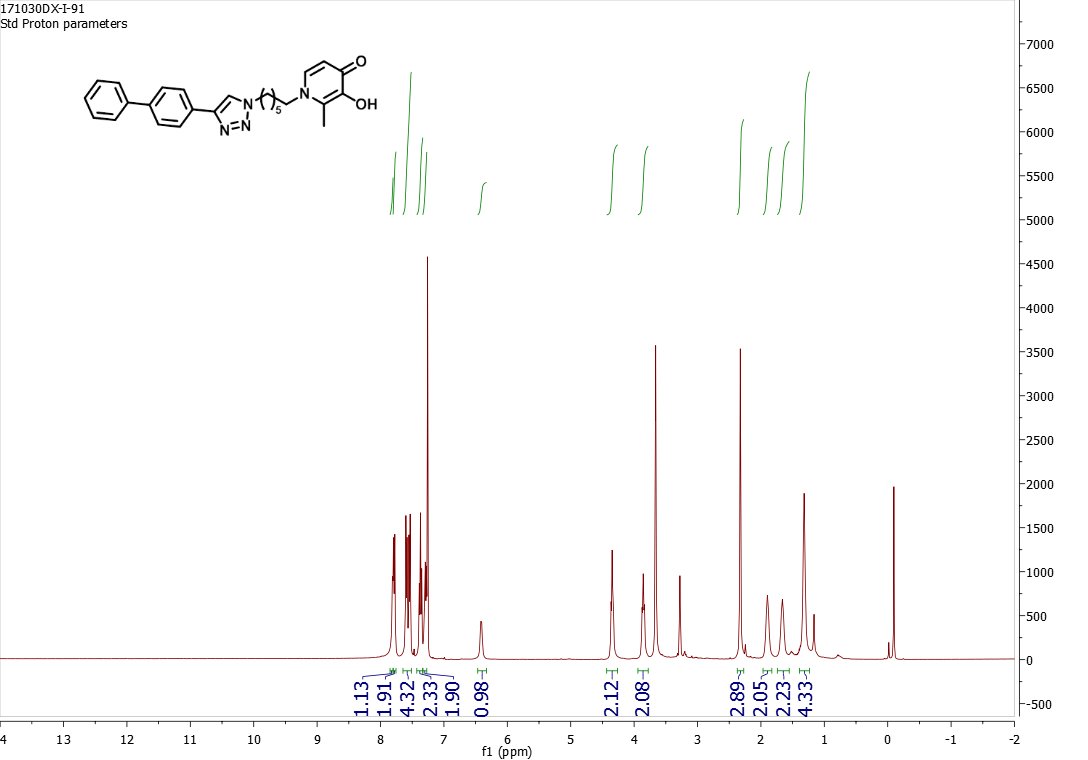


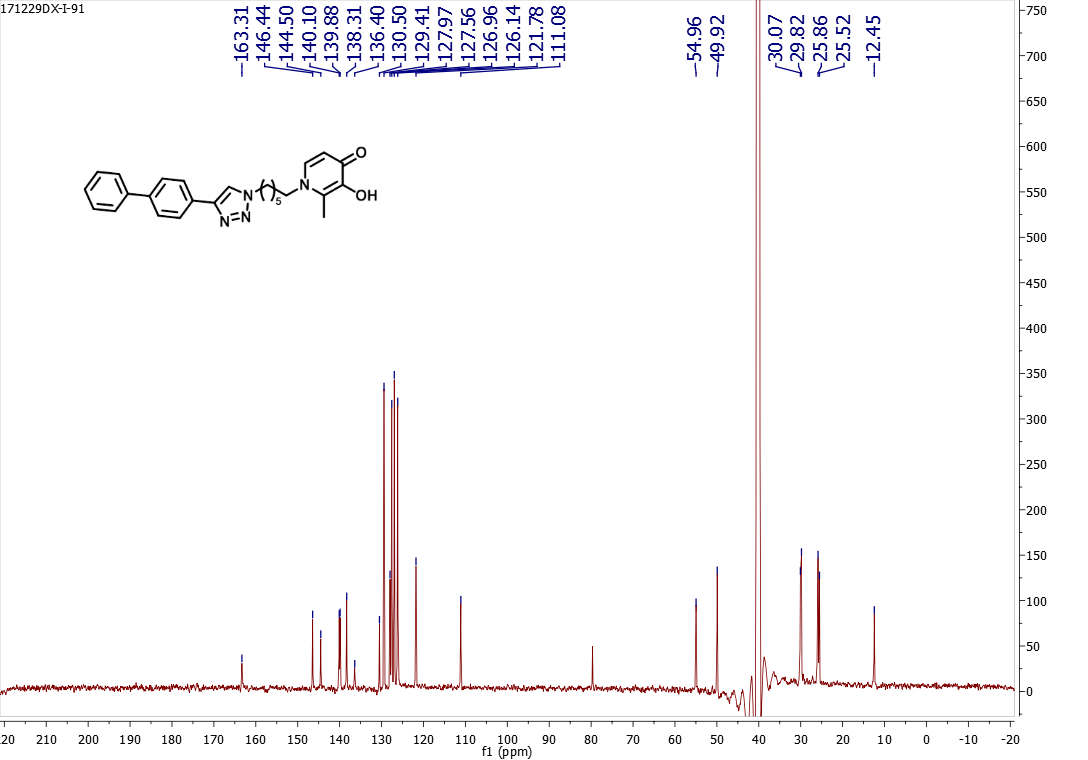


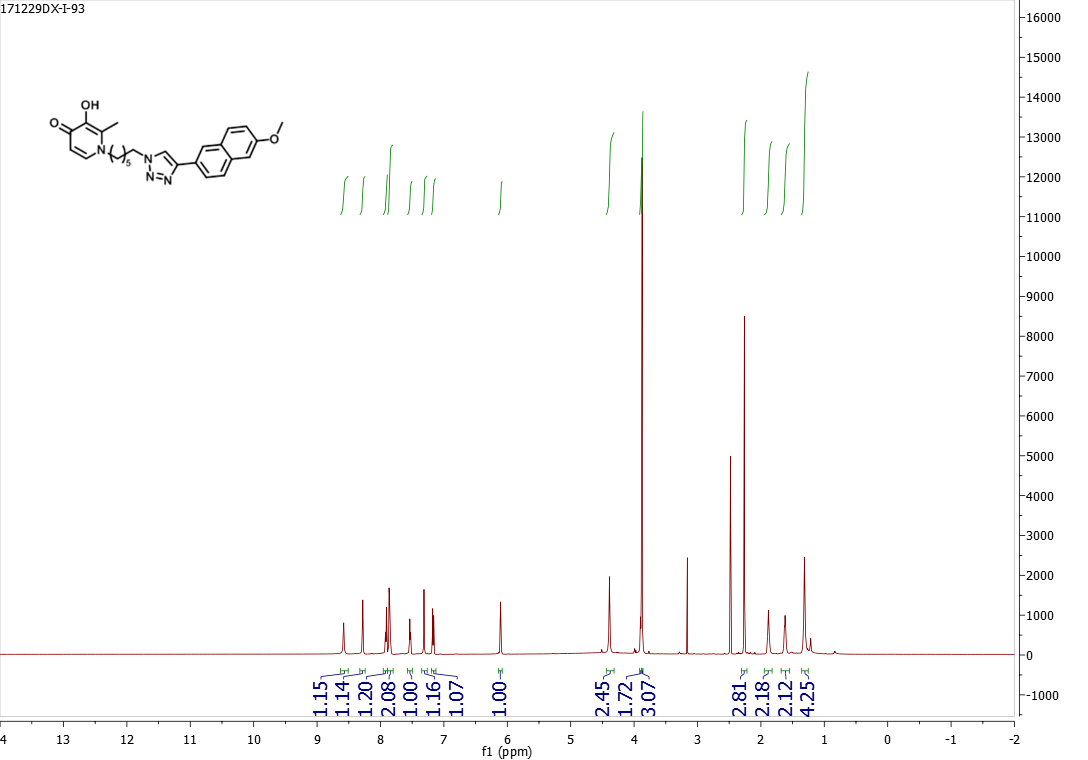


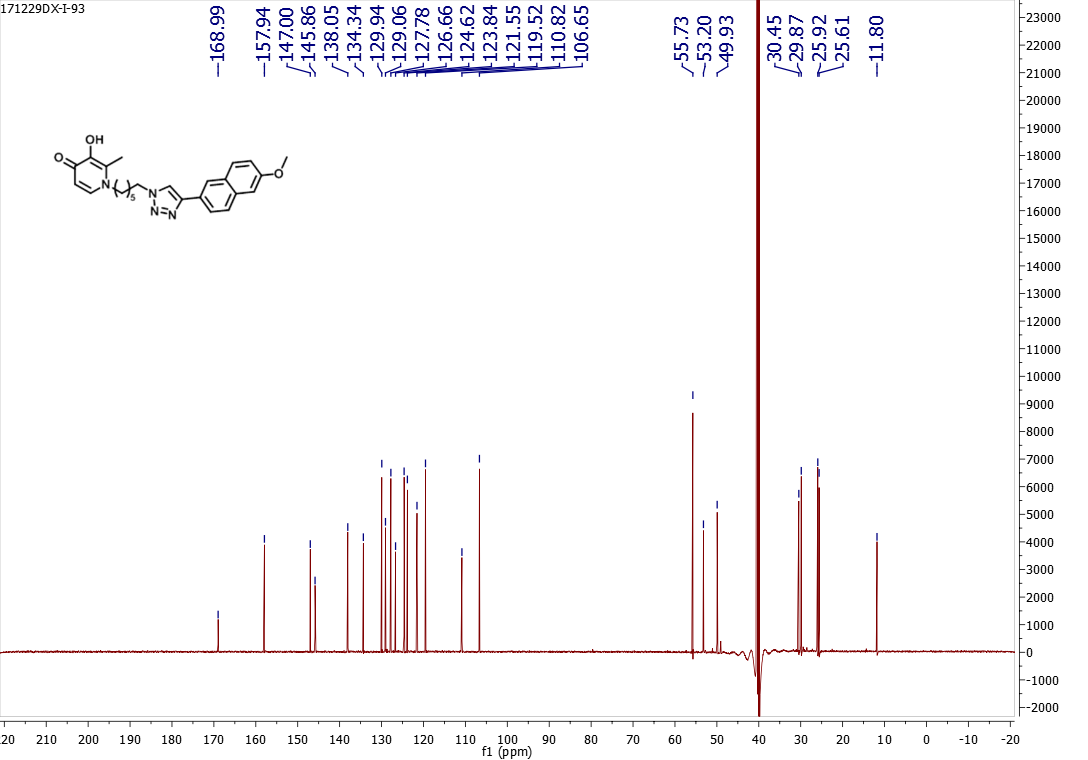

Supplement: Supplementary file 1 — Supplementary Information [file 41598_2019_39214_MOESM1_ESM.docx]
